# How Cool is That? The Effects of Menthol Mouth Rinsing on Exercise Capacity and Performance: A Systematic Review and Meta-analysis

**DOI:** 10.1186/s40798-024-00679-8

**Published:** 2024-02-21

**Authors:** Erica H. Gavel, Gabriel Barreto, Kierstyn V. Hawke, Trent Stellingwerff, Lewis J. James, Bryan Saunders, Heather M. Logan-Sprenger

**Affiliations:** 1 Faculty of Science, Ontario Tech University, Oshawa, ON Canada; 2 Applied Physiology and Nutrition Research Group, Center of Lifestyle Medicine, Faculdade de Medicina FMUSP, Universidade de São Paulo, São Paulo, Brazil; 3 Faculty of Health Science, Ontario Tech University, Oshawa, ON Canada; 4 Canadian Sport Institute - Pacific, Victoria, BC Canada; 5 Exercise Science, Physical and Health Education, University of Victoria, Victoria, BC Canada; 6 National Centre for Sport and Exercise Medicine, School of Sport, Exercise and Health Sciences, Loughborough University, Loughborough, UK; 7 Institute of Orthopaedics and Traumatology, Faculty of Medicine FMUSP, University of São Paulo, São Paulo, Brazil

**Keywords:** Menthol, Mouth rinsing, Exercise, Capacity, Performance

## Abstract

**Background:**

Menthol (MEN) mouth rinsing (MR) has gained considerable interest in the athletic population for exercise performance; however, the overall magnitude of effect is unknown.

**Objective:**

The aim of this systematic review and meta-analysis was to determine the efficacy of menthol MEN MR and the impact it has on exercise capacity and performance.

**Methods:**

Three databases were searched with articles screened according to the inclusion/exclusion criteria. Three-level meta-analyses were used to investigate the overall efficacy of MEN MR and the impact it has on exercise capacity and performance. Meta-regressions were then performed with 1) mean *V*O2_peak_, 2) MEN swilling duration; 3) the MEN concentration of MR solution, 4) the number of executed swills throughout a single experiment, 5) the use of flavoured sweetened, non-caloric, or non-flavoured neutral solutions as controls, 6) mean environmental temperature at the time of exercise tests, and 7) exercise type as fixed factors to evaluate their influence on the effects of MEN MR.

**Results:**

Ten MEN MR studies included sufficient information pertaining to MEN MR and exercise performance and capacity. MR with MEN resulted in no significant change in capacity and performance (SMD = 0.12; 95% CI − 0.08, 0.31; *p* = 0.23, *n* = 1, tau^2^1 < 0.0001, tau^2^2 =  < 0.0001, *I*^2^ = 0%). No significant influence was detected in meta-regressions for *V*O2_peak_, (estimate: 0.03; *df* = 8; 95% CI − 0.03, 0.09; *p* = 0.27), swilling duration (5 vs. 10 s: 0.00; *df* = 16; 95% CI − 0.41, 0.41; *p* = 1.0), MEN concentration (low [0.01%] vs. high [0.1%]: − 0.08; *df* = 15; 95% CI − 0.49, 0.32; *p* = 0.67), number of swills (estimate: 0.02; *df* = 13; 95% CI − 0.05, 0.09; *p* = 0.56), the use of flavoured sweetener or non-caloric as control (non-flavoured vs. flavoured: 0.12; *df* = 16; 95% CI − 0.30, 0.55; *p* = 0.55) or mean room temperature during exercise tests (estimate: 0.01; *df* = 16; 95% CI − 0.02, 0.04; *p* = 0.62).

**Conclusion:**

MEN MR did not significantly improve overall exercise capacity and performance, though those involved in endurance exercise may see benefits.

**Supplementary Information:**

The online version contains supplementary material available at 10.1186/s40798-024-00679-8.

## Key Points

- Menthol mouth rinsing may benefit endurance exercise.
- Physical fitness does not seem to be related to the efficacy of menthol mouth rinsing.

**Supplementary Information:**

The online version contains supplementary material available at 10.1186/s40798-024-00679-8.

## Background

Menthol (MEN), a chemical structure which presents itself as both a flavour and fragrance [1], has historically been found in a variety of products, with the most recent being a mouth rinse (MR), potentially used to enhance exercise capacity and performance [2]. Evidence has demonstrated that MEN MR may exert ergogenic effects in hot environments [3], endurance exercise [4], cycling and running [5, 6], and in females and males [4, 7]. The primary hypothesized mechanism by which MEN improves exercise performance is through activation of the transient receptor potential membrane 8 ion channel (TRPM8), found in the primary afferent sensory neurons whose cell bodies are located in the dorsal root and trigeminal ganglia, and its effect on the central nervous system [8]. More specifically, activation of TRPM8 is known to increase activity in the reward centres of the brain and is related to an increase in dopaminergic activity [9]. Regardless of the aforementioned evidence suggesting that MEN has the capacity to improve endurance exercise outcomes, the magnitude of the beneficial effect varies between studies [10, 11], with the majority of studies demonstrating capacity and performance improvement in the range of ~ 0.5–6% [3, 4, 7, 10, 12–15] and some studies showing no effect [11, 16].

Differing results may be explained by differences in the type of protocol (e.g. exercise duration), exercise mode, fasting state [12, 15, 16], MR swilling duration [3, 7, 17], concentration [2], type of placebo comparator [6, 11], or environment [11]; however, no direct comparisons have yet been explored. As such, results appear to provide rather diverse outcomes. For example, Mundel and Jones [12] reported time to exhaustion at 65% watt max was increased by 7.9% when a 0.01% MEN was swilled for 10 s every 10 min. Similarly, Flood et al. [10] reported a 7.1% increase in time to exhaustion in a 16/20 rating of perceived exertion (RPE) clamped protocol when 0.01% MEN was swilled for 5 s 1.5 min before the start of the trial, and every 10 min until completion. In contrast, Parton et al. [4] used the same protocol as Flood et al. [10] and showed a mean improvement of 4.6% when MEN was swilled for 10 s at the same concentration, but this was not significantly different from placebo. With that, it is hard to determine the discrepancy in outcomes without accounting for other variables such as environmental temperature, general fitness levels, and exercise mode.

Considering the applicability and popularity of supplementation during competition [18], it is essential one understands variables associated with MEN MR and whether these fit the goal and context of the environment. While previous reviews and consensus statements have been completed in this area, it is hard to evaluate the efficacy of MEN MR as these were amalgamated with topical use and have the chance of being biased [2, [19]. As such, the aim of this meta-analysis was to take an objective approach and answer the following questions: (1) determine whether MEN MR significantly improves performance and capacity, and (2) subsequently determine if volume of peak oxygen consumed (*V*O2_peak_), swilling duration, concentration, number of swills, type of comparator, mean environmental temperature, and exercise type (endurance vs. power/strength) influence how one may respond to MEN MR. We hypothesized that menthol would significantly improve capacity and performance—particularly during endurance exercise, *V*O2_peak_ will not be correlated with the efficacy of MEN MR, and swilling duration, concentration, number of swills, and mean environmental temperature will be positively correlated with improvements in performance and/or capacity and MEN MR. Lastly, we hypothesized that type of comparator will influence the efficacy of MEN MR.

## Methods

### Study Eligibility

The study protocol was designed in accordance with Preferred Reporting Items for Systematic Reviews and Meta-Analyses (PRISMA) guidelines [20] and the inclusion criteria defined according to PICOS (Population, Intervention, Comparator, Outcomes, and Study design) criteria. Only original human studies, English-language, and peer-reviewed journal articles were included. While the initial purpose of this meta-analysis was to analyse carbohydrate, caffeine, and MEN MR, we decided that it would be more advantageous to publish all meta-analyses separately. As such, the literature was screened to identify all studies investigating the effect of only MEN MR and exercise capacity and performance. Data extraction and meta-analysis were subsequently based only on studies that used MEN MR. The population included were able-bodied, recreationally active, trained, and/or elite human participants. No study included sedentary or inactive participants. The intervention required MEN MR prior to or during an exercise task, with a placebo, water, or control (no MR) as the comparator. For the outcomes, studies must have evaluated exercise capacity or performance in a crossover study design.

### Search Strategy

**Fig. 1:**
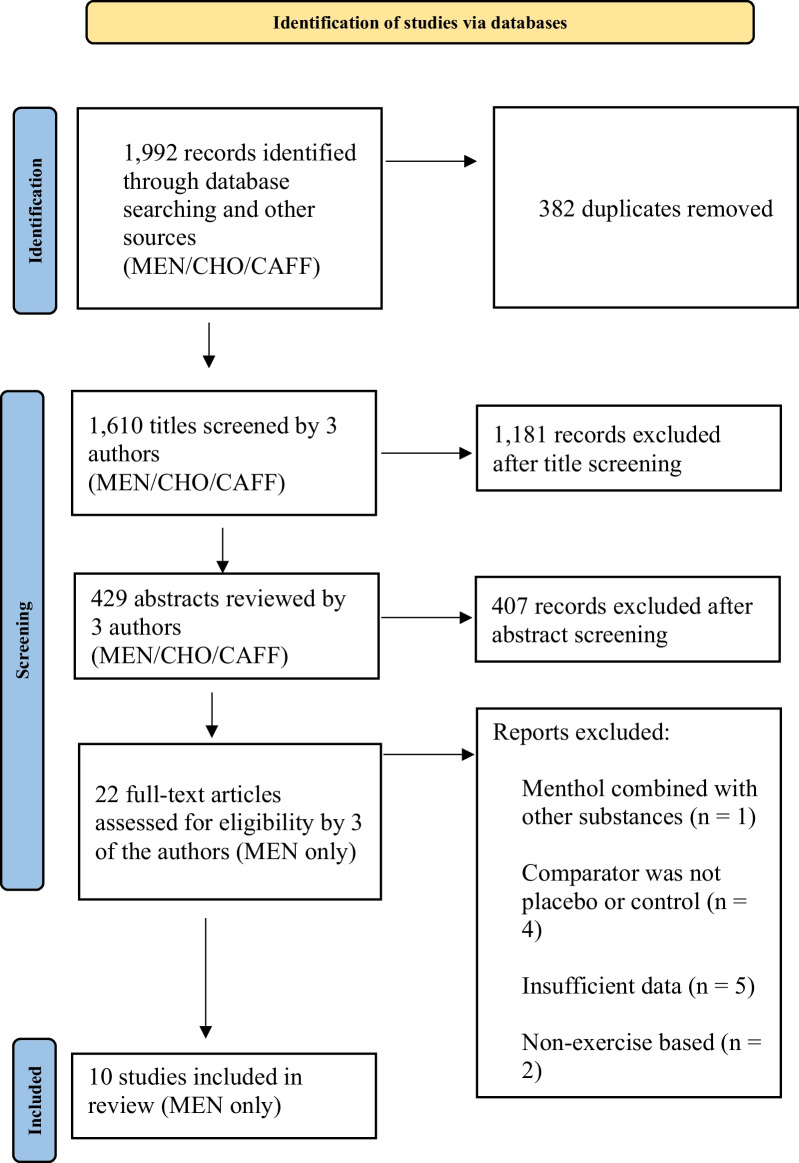
Flowchart of the search strategy and study selection

An electronic search of the literature was undertaken using three databases (SPORTDiscus, Medline, and Web of Science) to identify all articles relevant for the meta-analyses. The initial search was performed 15 June 2021 (Medline, *n* = 746; SPORTDiscus, *n* = 177; Web of Science, *n* = 1081), with the final search performed at the end of July 2023 to identify all indexed articles up to that point, Fig. 1. The final search was conducted through PubMed and included the same search terms as the original databases with the date range of 15 June 2021 to 31 July 2023, resulting in the addition of one further article to the current meta-analyses. The first-order search terms used were: men, OR women, OR male, OR female, OR athlet*, OR participant*, OR subject*, and were used in conjunction with the second-order search terms, mouth rins*, OR mouth wash*, OR mouthwash*, OR oral rins*, OR oral wash*, OR swill*, and the third-order search terms, exercis*, OR performance, OR physical activit*, OR fitness, OR ‘time trial’, OR ‘time to exhaustion’, OR sprint, OR run*, OR cycl*, OR ‘time to fatigue’, OR endur*. Duplicates were removed before a three-phase strategy was performed independently by three different researchers (EG, KH, HLS). Stage one assessed the eligibility of the title, whereas stage two assessed the abstract. Studies with uncertain suitability were included in phase two, whereby the final decision was made upon evaluation of the abstract in stage two. Stage three consisted of full-text review. Reference lists of review articles that met these criteria were also screened. Any differences regarding opinion were resolved through discussion (EG, KH, HLS).

### Data Extraction and Variable Categorization

Data extraction was conducted by EG, KH, and HLS using a standardized extraction sheet. When data were available only in figures, mean and standard deviation (SD) values were obtained with the Rstudio package ‘digitize’ tool [11, 14, 15] by GB. Individual data from Crosby et al. [16] were obtained via email. Information that was extracted included the following: authors and year of publication, population characteristics (sex, hours per week of training, and *V̇*O2_peak_), exercise task, exercise protocol, environmental conditions (degrees Celsius (°C), % relative humidity (RH)), MR protocol (concentration, frequency, total # of MRs, swilling duration, and comparator), exercise time mean and standard deviation, and level of significance (*p-*values).

### Risk of Bias Assessment

The studies included in this meta-analysis were assessed for risk of bias according to the Cochrane Collaboration’s recommendations for systematic reviews and meta-analyses which include [21]: (a) Random sequence generation; (b) Allocation concealment; (c) Blinding of participants and personnel; (d) Blinding of outcome assessment; e) Incomplete outcome data; and (f) Selective reporting. These aspects were categorized as ‘unclear risk’, ‘low risk’, or ‘high risk’ of bias. Two researchers (EG and KH) independently assessed the articles’ risk of bias. Any disagreements were resolved through discussion. Risk of bias was assessed with a revised tool in randomized controlled trials (Cochrane Risk of Bias 2 [ROB 2] tool) [22]. Risk of bias was judged to be ‘high’ if at least one domain was high risk or more than three domains had ‘some concerns’, judged to be ‘some concerns’ if at least one domain had ‘some concerns’, and judged to be ‘low’ if all domains were considered low risk.

### Quality of Evidence

Outcomes were rated according to the Grading of Recommendations, Assessment, Development, and Evaluations (GRADE) Framework [23]. Certainty of evidence could be considered as ‘very low’, ‘low’, ‘moderate’, or ‘high’ depending on the number of downgrades attributed to each of the five topics, (1) risk of bias, (2) imprecision, (3) inconsistency, (4) indirectness, and (5) publication bias. *Risk of bias* was rated based on the outline in section ‘Risk of bias assessment’. *Impression* was deemed to be present if outcomes were calculated from only a few studies with small sample sizes, or if decision making would differ when the lower and upper confidence limits were considered the real effect. *Publication bias* was determined by assessing funnel plots. *Indirectness* was deemed if the study did not use a placebo or control as comparator, whereas *inconsistency* was determined according to the heterogeneity measures (*I*^*2*^ or tau^2^) [23].

### Statistical Analysis

All analyses were performed with the Rstudio Software (Rstudio 1.4.1103, PBC, USA). Extracted means and SDs were converted to standardized mean differences (SMDs: Hedges’ g), and standard errors (SEs) with the *esc_mean_sd* function from the *esc* package. Pooled SD was calculated using only the SD from the control condition since homogeneity of variances was considered for all studies. For outcomes in which time to completion was the most important variable, means were multiplied by − 1 for conversion into SMDs and SEs. Whenever more than one datapoint was available for the same exercise test (i.e. repeated measure or time splits) [16, 24], SMDs and variances were combined as described by [25]. Since a correlation between timepoints could not be obtained, an r value of 0.7 was always assumed. A SE was then obtained by calculating the square root of the calculated variances. With the obtained SMDs, a three-level random effects meta-analysis was performed (*metagen* function from the *meta* package), so that a combined variance could be calculated and considered within studies that reported more than one exercise test (i.e. more than one outcome). Variance estimates (tau and tau-squared) were calculated with the restricted maximum-likelihood estimator (REML), and its confidence intervals (CI) were estimated with the profile-likelihood method. A subjective analysis of the funnel plot was performed for the risk of imbalances from the effects of single studies. Egger’s regressions were performed to verify if small studies with large effect sizes could have influenced the results of this meta-analysis [26]. Heterogeneity between studies was assessed with *I*^2^ statistics, with values ≤ 50% indicating low heterogeneity, 50–75% moderate heterogeneity and > 75% high heterogeneity. Hedges’ g values were categorized as small (≤ 0.2), medium (0.2–0.5), large (0.5–0.8), and very large (> 0.8) [27].

Meta-regressions were then performed (*metareg* function of the *meta* package) with 1) mean *V̇*O2_peak_ values representing the participant’s general fitness level (continuous), 2) MEN swilling duration (binomial, 5 or 10 s); 3) the MEN concentration of MR solution (binomial, 0.1 or 0.01%), 4) the number of executed swills throughout a single experiment (continuous), 5) the use of flavoured sweetened, non-caloric, or non-flavoured neutral solutions as controls (binomial, flavoured or non-flavoured), 6) mean environmental temperature at the time of exercise tests (continuous), and 7) exercise type (binomial, endurance vs. power/strength) as fixed factors to evaluate their influence on the effects of MEN MR. Estimates for the effect sizes of each of the two exercise types were estimated with the *predict* function. An additional exploratory meta-regression was performed with sex as fixed factor, which accounted for the presence of females in the observed samples (binomial, yes—included females vs. no—did not include). Statistical significance was set at p < 0.05.

## Results

### Study Search and Characteristics

Outcomes in which the control condition was considered to cause any bias (i.e. capsaicin [10]) and in those in which the MEN MR intervention was combined with other interventions (i.e. MEN combined with crushed ice [14] or a water spray [28]) were excluded from all analyses. The final number of ten studies, which included 22 outcomes comprising 153 individuals, consisting of 115 males and 38 females, were incorporated in the final analysis, as given in Table 1.

**Table 1:** Studies included in the systematic review and meta-analysis

Study	# Of participants	# Of training hours per week	*V̇*O2_peak_ (mL·kg·^−1^ min^−1^)	Modality	Exercise and outcome description	Environmental conditions	MR concentration	MR frequency	Total # of MR	Swilling duration	MR comparison	Difference	Significant performance or capacity effect
Mundel and Jones [12]	9 M	NR	54 (5)	Cycle	65% of W_max_ to exhaustion	34 (1) °C, 27 (4) % RH	0.01%	Every 10 min	Variable	10 s	Orange-flavoured placebo	7.9%	*p* = 0.043
Stevens et al. [13]	11 M	> 4 h	NR	Run	5-km time trial	32.6 (0.2) °C, 45.8 (5.7) %RH	0.01%	At the 0.2 km mark of every 1 km	5	5 s	Control—no mouth rinse	2.7%	*p* < 0.01
Flood et al. [10]	8 M	> 5 h	55.4 (6)	Cycle	RPE 16/20 to exhaustion	35.0 (0.8) °C, 47.8 (2.3) %RH	0.01%	1.5 min prior and every 10 min during (− 1:30, 8:30, 18:30 min)	3	5 s	Apple favoured non-calorific artificial sweetener (sucralose)	7.1%	*p* = 0.049
Gibson et al. [11]	11 M, 3 F	NR	46.2 (12.9)	Cycle	40-min intermittent sprint protocol: W_peak_ and work done	40.2 (0.6) °C, 42 (2) %RH	0.01%	Before and every 10 min throughout	4	5 s	Water	0%	*p* = 0.981
Gibson et al. [11]	11 M, 3 F	NR	46.2 (12.9)	Cycle	40-min intermittent sprint protocol: W_peak_ and work done	40.2 (0.6) °C, 42 (2) %RH	0.01%	Before and every 10 min throughout	4	5 s	0.3 sham-carbohydrate orange flavoured	1.4%	*p* = 0.981
Jeffries et al. [3]	11 M	NR	52.4 (5.3)	Cycle	70% W_max_ with self-selected cadence to exhaustion	35 (0.2) °C, 40 (0.5) %RH	0.01%	MR at 85% of time to exhaustion baseline	1	5 s	Raspberry-flavoured non-calorific	4.4%	*p* = 0.036
Jeffries et al. [3]	11 M	NR	52.4 (5.3)	Cycle	70% W_max_ with self-selected cadence to exhaustion	35 (0.2) °C, 40 (0.5) %RH	1.25 g/kg	85% of time to exhaustion baseline	1	5 s	Cold water with ice	− 1.6%	*p* > 0.05
Saldaris et al. [14]	12 M	NR	61.3 (2.6)	Run	3 × 30 min at 65% VO_2peak_ and TTE run at 100% VO_2 pea_	35.3 (0.3) °C, 59.2 (2.5) %RH	0.1%	Prior to trial, 15 and 30 min point of each run block, and prior to the TTE run	10	5 s	Control—no mouth rinse	34.4%	*p* = 0.020
Best et al. [15]	10 M, 9 F	> 2 h for + 3 months prior	NR	Dynamometer	Isometric mid-thigh pulls mean force (max strength/power) 3 × 3-s max effort separated by 3-min rest	22 (1) °C, NR	0.1%	60 s prior to each exercise	9	10 s	Control—no mouth rinse	0.5%	*p* = 0.002
Best et al. [15]	10 M, 9 F	> 2 h for + 3 months prior	NR	Legs	Vertical jump height 3 × 3-s max effort separated by 3-min rest	22 (1) °C, NR	0.1%	60 s prior to each exercise	9	10 s	Control—no mouth rinse	0.5%	*p* = 0.013
Best et al. [15]	10 M, 9 F	> 2 h for + 3 months prior	NR	Cycle	6-s sprint average power 3 × 3-s max effort separated by 3-min rest	22 (1) °C, NR	0.1%	60 s prior to each exercise	9	10 s	Control—no mouth rinse	0.5%	*p* = 0.120
Gavel et al. [7]	9 F	14.3 (2.9)	50.8 (6)	Cycle	30-km time trial	30 (0.6) °C, 70 (1) %RH	0.01%	Every 5 km	5	5 s	Non-caloric berry-flavoured sweetener (sucralose)	2.2%	*p* = 0.034
Parton et al. [4]	11 M	< 5	53.9 (6.9)	Cycle	RPE 16/20 to exhaustion	34.9 (0.5) °C, 40.6 (2.2) %RH	0.01%	30 s prior to and at 10-min intervals (− 0:30, 9:30, 19:30 min)	Variable	10 s	Apple flavoured non-calorific artificial sweetener (sucralose)	6.5%	*p* = 0.039
Parton et al. [4]	11 F	< 5	43.5 (2.9)	Cycle	RPE 16/20 to exhaustion	34.9 (0.5) °C, 40.6 (2.2) %RH	0.01%	30 s prior to and at 10-min intervals (− 0:30, 9:30, 19:30 min)	Variable	10 s	Apple flavoured non-calorific artificial sweetener (sucralose)	2.2%	*p* = 0.475
Crosby et al. [16]	6 M, 6 M	3–7	NR	Cycle	3-min all out	33 (3) °C, 46 (5) %RH	0.1%	66% (2 min) of time trial	1	5 s	Cold water (< 4 °C)	NR	*p* = 0.540
Crosby et al. [16]	6 M, 6 M	3–7	NR	Cycle	3-min all out	33 (3) °C, 46 (5) %RH	0.1%	66% (2 min) of time trial	1	5 s	Peppermint	NR	*p* = 0.540
Jerram et al. [24]	27 M	> 10 training sessions per week	NR	15-a-side rugby-specific conditioning blocks	Distance per minute	26.8 (1.6)°C, 58.2 (3.4) %RH	0.1%	Warm-up (55 min), 3-min rugby-specific conditioning block, 1-min rest, 3-min rugby-specific conditioning block, 1-min rest, 30 min of further training	3	5 s	Water	0.5%	*p* = 0.257

Data are presented as mean (SD). M, males; F, females; h, hour; NR, not reported; W_max_, maximal watts; W_peak_, peak watts; TTE, time to exhaustion; s, seconds; %, per cent; min, minute; °C, degrees Celsius; %RH, percentage of relative humidity; VO_2peak_, peak oxygen consumption; MR, mouth rinse; RPE, rating of perceived exertion

### Meta-analysis

**Fig. 2:**
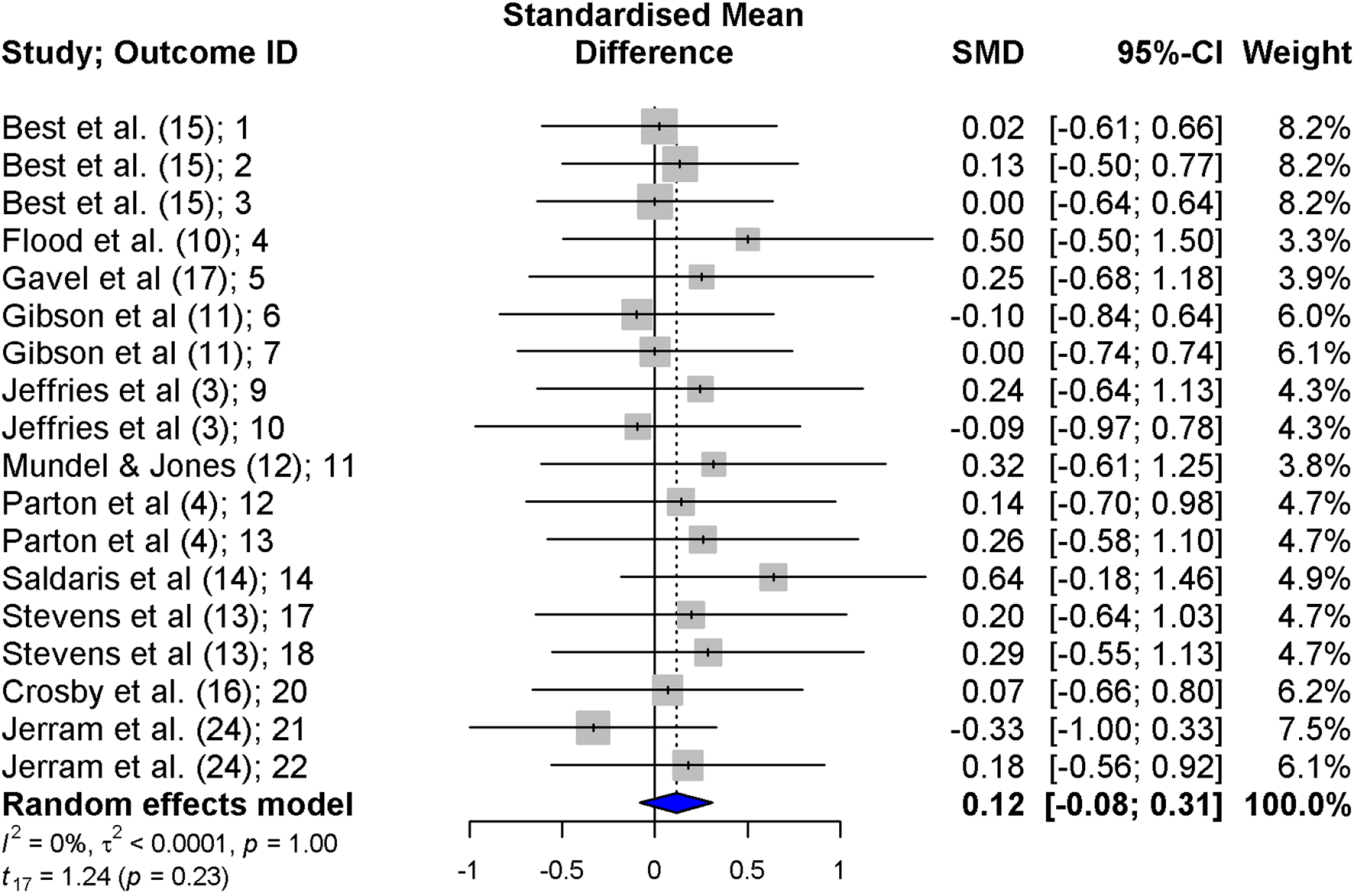
Forest plot of menthol mouth rinsing studies included in this meta-analysis. Positive values indicate a capacity and performance enhancement of MEN MR versus control condition. Titles on the left side refer to ‘Author (reference number); unique ID assigned to each included outcome’. The reason for missing IDs (i.e. 8, 15, 16, and 19) is the exclusion of some of the outcomes in a phase posterior to unique ID assignment

**Fig. 3:**
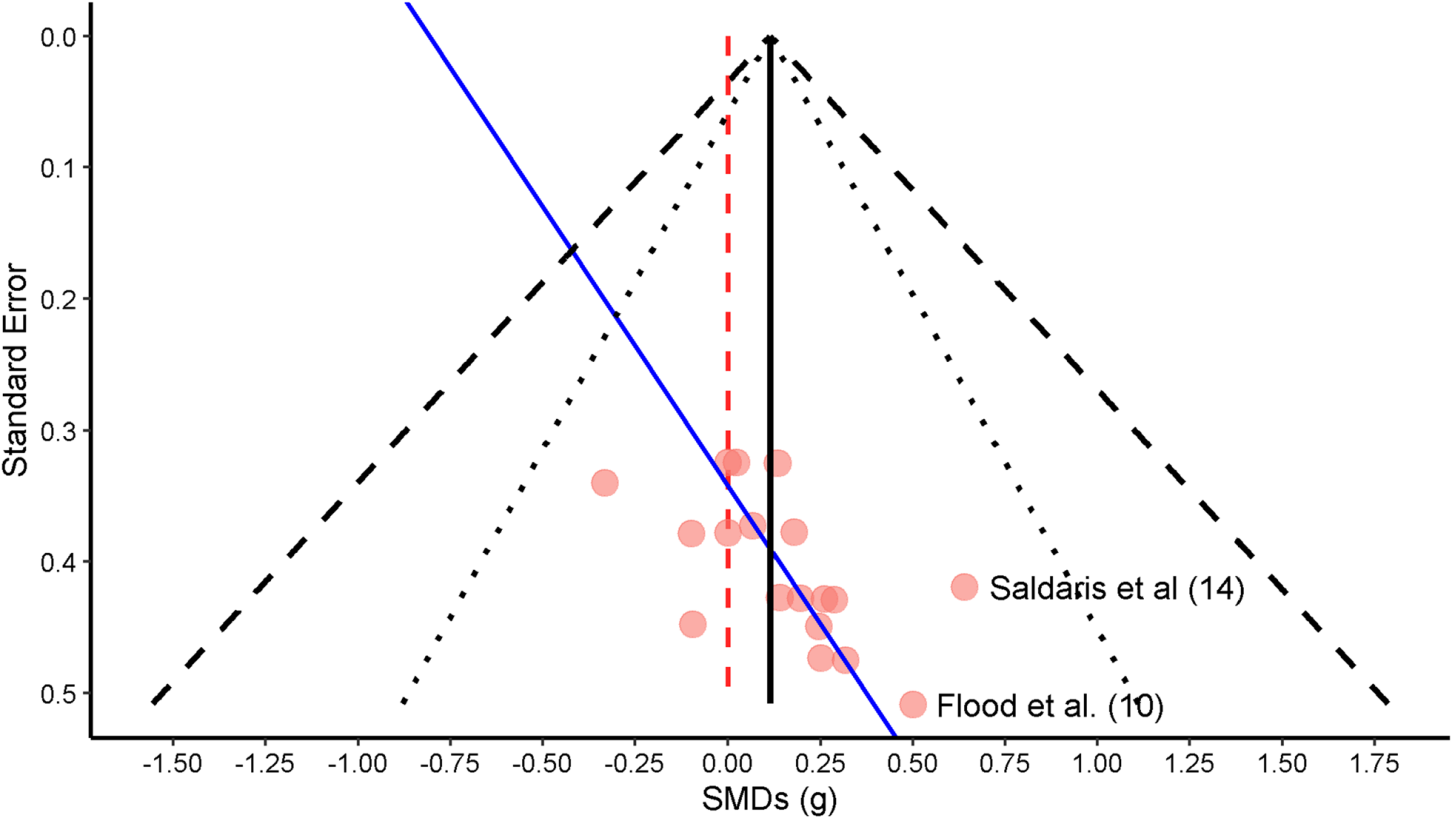
Funnel plot of menthol mouth rinsing studies included in this meta-analysis. Both highlighted studies were considered to potentially influence the effect sizes. Pink dots represent individual effect sizes. The central black line represents the observed main estimate of this meta-analysis, while the black dotted and dashed lines represent the 95 and 99% confidence intervals, respectively. The blue line represents the Egger’s regression line illustrating the influence and the direction of the effect of smaller studies (i.e. with higher standard error values) on the obtained effect sizes

MR with MEN resulted in no significant change in capacity and performance (SMD = 0.12; 95% CI − 0.08, 0.31; *p* = 0.23, n = 1, tau^2^1 < 0.0001, tau^2^2 =  < 0.0001, *I*^2^ = 0%; Fig. 2). After visualization of the funnel plots for publication bias assessment, some asymmetry was considered to exist. Two data points with large SMDs and equally large variances were identified [10, 14] (Fig. 3). An Egger’s regression test confirmed the potential existence of small-study effect bias (Intercept = − 0.81, bias (slope) = 2.37; *p* = 0.01), and the resulting regression line is represented in Fig. 3. A sensitivity analysis was performed with the exclusion of both studies, with similar results (SMD = 0.07; 95% CI − 0.13, 028; *p* = 0.46, n = 133, tau^2^1 < 0.0001, tau^2^2 =  < 0.0001, I^2^ = 0%). When repeating the Egger’s regression after the exclusion of both studies, bias was still present (Intercept = − 0.64, bias (slope) = 1.86; *p* = 0.03). Therefore, all regressions were performed with both studies included.

### Meta-regressions

No significant influence was detected in meta-regressions for *V̇*O2_peak_, (estimate: 0.03; *df* = 8; 95% CI − 0.03, 0.09; *p* = 0.28, *n* = 84), swilling duration (5 vs. 10 s: 0.00; *df* = 16; 95% CI − 0.41, 0.41; *p* = 1.0, *n* = 153), MEN concentration (low [0.01%] vs. high [0.1%]: − 0.08; *df* = 15; 95% CI − 0.49, 0.32; *p* = 0.67, *n* = 144), number of swills (estimate: 0.02; *df* = 13; 95% CI − 0.05, 0.09; *p* = 0.56, *n* = 122), the use of non-caloric flavoured sweetener or no MR as a control (non-flavoured vs. flavoured: 0.12; *df* = 16; 95% CI − 0.30, 0.55; *p* = 0.55, *n* = 153) or mean environmental temperature during exercise tests (estimate: 0.01; *df* = 16; 95% CI − 0.02, 0.04; *p* = 0.62, *n* = 153). An additional meta-regression found no influence of sex in the effects of menthol mouth rinsing (females not included vs. included: − 0.11; *df* = 16; 95% CI − 0.50, 0.29, *p* = 0.58, *n* = 153). No effect of exercise type was detected by the meta-regression (endurance vs. others: − 0.08; *df* = 16; 95% CI − 0.54, 0.37; *p* = 0.70). Nonetheless, estimated effect sizes were larger for endurance (SMD: 0.14; *df* = 16; 95% CI − 0.09, 0.36, *n* = 134), than for strength/power (SMD: 0.05; *df* = 16; 95% CI − 0.34, 0.35, *n* = 19). When endurance studies alone were pooled, no effect of exercise duration was seen in a meta-regression (estimate: − 0.00; *df* = 10, 95% CI − 0.00, 0.00, *p* = 0.78).

### Risk of Bias

**Fig. 4:**
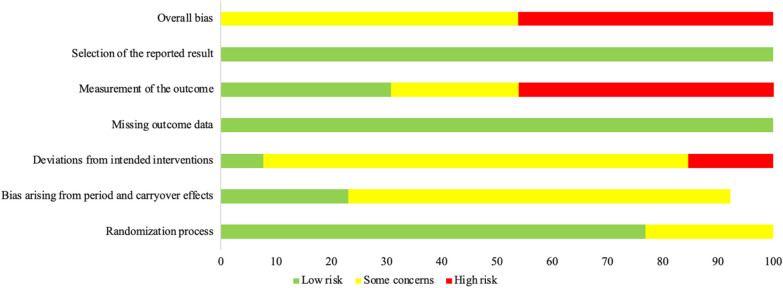
Risk of bias presented as percentages across all included for the six main domains of evaluation. Green, low risk; yellow, somewhat concerned; red, high risk

None of the studies were classified as having a low risk of bias, with 53.8% considered as having some concerns and the remaining 46.2% as high risk. Most of the studies had issues in the second (92.3% as some concerns or high risk) and fourth domain (69.3% as some concerns or high risk) due to bias arising from lack of a ‘true’ placebo or familiarization. The majority of studies were judged as low risk of bias arising from the first and third domain of the ROB 2 tool. A summary of the results are presented in Fig. 4.

### GRADE Certainty of Evidence

Most outcomes were considered to have a very low level of certainty of evidence. Risk of bias was high as most studies had issues in the second and fourth domain. After visual examination, asymmetry was detected in the funnel plots indicating that publication bias existed. Imprecision was deemed to have occurred in 10 out of the 10 outcomes with very low certainty, mostly due to large confidence intervals. Indirectness was evident for power and short duration types of exercise. Evidence for endurance-based exercise in male individuals, female individuals, cycling, and running was considered as high certainty.

## Discussion

The current systematic review and meta-analysis focused on the level of evidence and impact MEN MR has on exercise capacity and performance. The methodology used to select MEN MR studies revealed that there is a low level of scientific evidence suggesting that a MEN MR does not significantly (SMD = 0.16; 95% CI − 0.07, 0.38; *p* = 0.13, Fig. 2) improve exercise performance from a group standpoint.

### Menthol Mouth Rinse Effects on Capacity and Performance

The results from this meta-analysis demonstrate that MEN MR did not significantly improve exercise capacity and performance; however, improvements seemed to be greatest during endurance exercise. Despite most individual studies showing a significant improvement, the overall mean change was not significant. This is in contrast to the meta-analysis by Jeffries and Waldron [2] which reported that MEN significantly improved performance. However, it should be noted that the meta-analysis by Jeffries and Waldron [2] analysed both included both internal and external application of MEN, and exhibited greater effects with application internally (Hedges’ *g* = 0.40, 95% CI 0.04–0.76, *p* = 0.03). Furthermore, while the present meta-analysis reported a SMD = 0.16, the meta-analysis of Jeffries and Waldron [2] reported a SMD = 0.33, a value more than 2 times greater than that reported in the current review. This difference between the two meta-analyses may be due to the number of studies included in the meta-analyses (current review, *n* = 10; Jeffries and Waldron [2], *n* = 13), methodological differences across investigations, as well as the inclusion of five studies in the present review that were not available in that of Jeffries and Waldron [2]. From a methodological perspective, while the present review only included articles in which the comparator was either a control (no MR) or a placebo (non-caloric, water, or non-mint containing), the meta-analysis by Jeffries and Waldron [2] included all studies in which MEN was compared to anything without the containment of MEN (e.g. inclusive of carbohydrate content etc.). Additionally, Jeffries and Waldron [2] included all published studies on MEN, inclusive of sedentary and active participants, whereas the present review included active participants only. Given that work by Foster et al. [29] and Hibbert et al. [30] suggests that current fitness levels and experience can impact the reliability of outcomes, one may conclude that the study inclusion criteria and participant population had influence over the strength of the results, possibly explaining the difference between the two reviews.

Our findings demonstrate that there was no relationship between MEN concentration, swilling duration, or mean environmental temperature during the tests. While no study has directly compared any of the aforementioned factors with menthol, we can postulate that the present results are in agreement with work by James et al. [31]. To illustrate, published work by James et al. [31] reported that there was no dose–response effect of carbohydrate MR concentration of 7 and 14% maltodextrin on 1 h cycling time trial performance (7%, 57.3 ± 4.5 min; 14%, 57.4 ± 4.1 min, *p* = 0.737). Similarly, while the present study observed no impact of swilling duration and performance improvement, in carbohydrate MR swilling duration work by Sinclair et al. [32] analysing the difference between 5 and 10 s during a 30-min self-selected time trial was able to show that 10 s of swilling was significantly more effective than 5 s (20.4 ± 2.3 km, vs. 19.2 ± 2.2 km; *p* < 0.01). While this is the only carbohydrate MR study which compared the differences in swilling duration, work by Stevens et al. [6] suggests that the same trend could be observed with MEN MR. In their study, Stevens et al. [6] compared ice-slurry ingestion and MEN MR during a running time trial. The authors observed no significant improvement with the ice-slurry ingestion but a positive effect with the MEN MR. Given that the ice-slurry ingestion and MEN MR would activate the same thermoreceptors (TRPM8) located in the oral cavity, this suggests that exposure time of the MEN MR may influence performance.

In addition, the present meta-analysis observed no relationship between environmental temperature and MEN MR relative to physiological performance. Given that limited MEN MR research has been conducted in thermoneutral conditions (< 22 °C; *n* = 1 study [14]) with three primary outcomes (Table 1), it is hard to determine whether MEN MR is more beneficial in thermoneutral or hot environments. Although Best et al. [15] showed a significant improvement between MEN MR and the familiarization session, the comparison between the control and MEN MR was unclear. Moreover, it is important to note that the humidity was not reported and this could had a significant impact on total body heat strain [33]. Independent of the work done by Best et al. [15], the present review displayed environmental conditions between 30.0–40.2 °C and 40–70% RH with 8 of the studies between ~ 30.0–35.0 °C, and 1 at 40.0 °C. As such, it is hard to determine the overall influence environmental conditions have on MEN MR given the paucity of research in the area, and the fact that most athletes and teams use MEN MR in hot and humid conditions.

### Methodological Aspects

Certain aspects of the present review should be acknowledged. Firstly, we decided to only include original studies that used MEN MR and measured exercise capacity or performance. Although we tried to ensure homogeneity of the articles in this review, exercise modality was not accounted for and could have influenced the results. Secondly, given that anything ‘mint’ or ‘menthol’ flavoured stimulates the TRPM8 receptors, a ‘true’ placebo is not attainable and could have impacted the results. Given that work by Saunders et al. [34] showed that belief in a product may lead to positive improvements in exercise performance, this suggests that pre-trial preference could have impacted the results. Another factor that could have impacted outcomes is exercise protocol. In the present meta-analysis, protocols differed among each study which may describe the differences associated with MEN MR. We decided to include all modes of exercise given the relevance and applicability of supplementation in all types of activity [35]; however, it is unclear as to whether MEN MR would have a greater effect with different modes as the number of controlled studies on MEN MR across modes of exercise is limited.

Moreover, another methodological factor of the current review is that we only analysed data from crossover design studies. In some respects, the duration of time from one trial to the next differed among each study and may be considered as a confounding variable. In addition, no research has analysed the washout period or whether certain nutrition strategies influence the beneficial effects of MEN MR. Although work by Best et al. [36] investigated thermal perception and the time course following a MEN MR, no work has been done on thermoreceptors and brain activity. Assuming the MEN MR effects are dependent on time, one may suggest that a washout period and control of habitual MEN uses (i.e. menthol toothpaste, chewing gum) may influence the magnitude of the effect of MEN MR. Also, since the number of included outcomes was relatively small, it is unclear whether enough statistical power was reached for each of the sub-analyses performed (*V̇*O2_peak_, *n* = 9; swilling duration, *n* = 15; MEN concentration, *n* = 14; number of swills, *n* = 12; type of control, *n* = 12; mean room temperature, *n* = 15). Lastly, given that the original purpose of the project differed from the current version, the study was not pre-registered ahead of time. While we are unaware of any consequences associated with this, the lack of pre-registration could be a limitation.

### Level and Quality of Evidence Reviewed for Publication Bias

Overall, the analysis indicated a high risk of bias as the majority of studies did not use a familiarization trial or double-blinding procedures when working with the MEN MR. While the studies by Mundel and Jones [12], Flood et al. [10], Gavel et al. [7], and Crosby et al. [16] used a subject single-blinded design, the researchers involved with the procedures were aware of the MR allocation and substance rinsed in each trial. Furthermore, based on the current meta-analysis, it must be acknowledged that no MEN MR study has disclosed whether researchers involved with data analysis were blinded to manipulation and the risk of detection bias. As such, given the absence of information regarding outcome assessments, future MEN MR research should take this into account as it could negatively affect the assessment of outcomes and detection bias [21].

Another factor that could contribute to the high risk of bias is the lack of a ‘true’ placebo [37]. Given that studies did not describe how the purpose of the study was communicated to the participants, one could suggest that the lack of blinding could have impacted performance and be correlated with performance improvements. For example, in a short-duration high-intensity cycling time trial work by Mears et al. [38] investigating the effect of a semisolid breakfast containing carbohydrate verses a taste- and texture-matched placebo or water, it was shown that the performance was completed more quickly when subjects perceived that they had consumed breakfast. As such, it is hard to determine whether the improvement with MEN MR caused a true physiological change, or the improvement stemmed from the participants knowing the purpose of the study. Future research should explore this speculation.

### Future Directions and Research Considerations

Based on the current evidence available, MEN MR seems to be most useful in endurance sport. The results of this meta-analysis demonstrate the need for more high-quality research on MEN MR to elucidate the true effect of MEN MR. Given that a high number of studies did not include a familiarization trial, were not double-blinded, and failed to include information regarding outcome assessments, future work should follow the framework of Betts et al. [39] for Proper Reporting of Evidence in Sport and Exercise Nutrition Trials.

Furthermore, once methodological quality and study design have improved, other areas of future work include the need to understand the mechanisms of action, establish the dose (concentration)-response of MEN, compare single versus repeated dose effects on performance measures, determine the best timing of use of MEN in an endurance event (pre, during, later stages), and measure the repeatability of the effect of MEN for a given exercise test and the effect across various endurance tests (i.e. steady-effort vs. stochastic in nature) and intra- and inter-individual variability as a function of MEN habituation, along with exploring the washout period needed. Moreover, although some research does exist [40], future work should also evaluate the efficacy of MEN MR in combination with other products (Additional file 1).

## Conclusions

In summary, using the present methodology to review randomized crossover design MEN MR studies using a placebo or controlled trial with the outcome being exercise performance or capacity, this meta-analysis provides evidence that a MEN MR does not generally improve performance across all exercise modalities and study designs. However, it should be noted that MEN appears unlikely to harm performance, and at best, may have a small positive influence during endurance exercise. Thus, athletes may wish to systematically test this product in training to determine its efficacy for them. As such, MEN MR should be taken with caution until further research elucidates the optimal conditions in which one might benefit from MEN MR (Additional file 2).

### Supplementary Information

**Additional file 1:** Supplementary Regressions.

**Additional file 2:** Excluded Articles.

## Data Availability

The datasets used/analysed during the current study are available from the corresponding author on reasonable request.

## Abbreviations

MEN: Menthol
MR: Mouth rinse
TRPM8: Transient receptor potential membrane 8 ion channel
RPE: Rating of perceived exertion
s: Second
*V̇*O2_peak_: Volume of peak oxygen consumed
PRISMA: Preferred Reporting Items for Systematic Reviews
PICOS: Population Intervention Comparator Outcomes and Study Design
SD: Standard deviation
°C: Degrees Celsius
RH: Relative humidity
*p-*values: Level of significance
GRADE: Grading Recommendations, Assessment, Development, and Evaluations Framework
SMD: Standardized means difference
SE: Standard error
REML: Restricted maximum-likelihood estimator
CI: Confidence intervals
