# Supplementary material for: How Cool is That? The Effects of Menthol Mouth Rinsing on Exercise Capacity and Performance: A Systematic Review and Meta-analysis
Source: Sports Med Open. 2024 Feb 21;10:18. doi: 10.1186/s40798-024-00679-8 (PMC10881929; doi:10.1186/s40798-024-00679-8)
Supplement: Supplementary file 1 — Additional file 1: Supplementary Regressions. [file 40798_2024_679_MOESM1_ESM.docx]

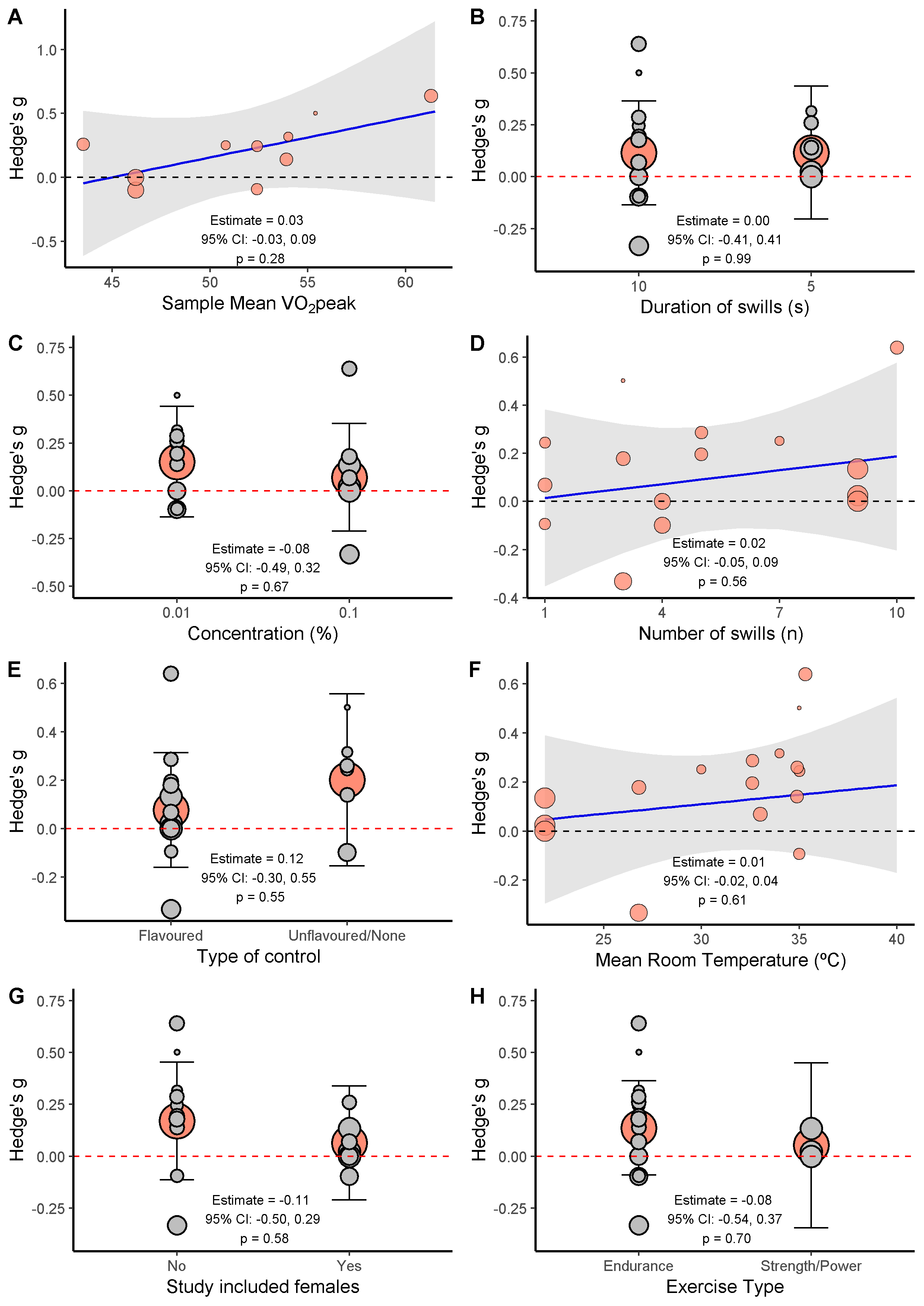


**Supplementary Figure1.** Pannels A to H refer to meta-regressions representing the influence of the following factors on the effect of menthol mouth-rinsing on exercise performance: A) continuous with VO_2_Peak, B) binomial comparing swill duration, C) binomial with concentration of menthol, D) continuous with number of swills during a single exercise trial, E) binomial with type of control utilized for comparison, F) continuous with mean room temperature during exercise trials, G) binomial with the inclusion of females, H) binomial with exercise type. On panels A, D and F, the blue line represents the resulting regression line, and the red dots represent effect sizes from individual studies. On panels B, C, E, G and H, the big red dots represent the mean SMD estimate, while grey dots represent the effect sizes of individual studies, and black lines represent the 95% confidence intervals.
