# Supplementary material for: How Cool is That? The Effects of Menthol Mouth Rinsing on Exercise Capacity and Performance: A Systematic Review and Meta-analysis
Source: Sports Med Open. 2024 Feb 21;10:18. doi: 10.1186/s40798-024-00679-8 (PMC10881929; doi:10.1186/s40798-024-00679-8)
Supplement: Supplementary file 2 — Additional file 2: Excluded Articles. [file 40798_2024_679_MOESM2_ESM.doc]

1. Mouthrinses and dental caries. Int Dent J, 2002. 52(5): p. 337-45.

2. Breath Aids. Men's Fitness, 2004. 20(5): p. 56-56.

3. Aktuelles in Kuerze: Leistungsverbesserung durch Mundspuelung. / News in brief: improving performance by rinsing the mouth. Leistungssport, 2005. 35(2): p. 30-31.

4. The White Stuff. 2008. p. 88-88.

5. Carbohydrate mouth rinsing. Peak Performance, 2009(274): p. 11-12.

6. It worked for me. Cycling Weekly, 2009: p. 47-47.

7. Abad-Santos, F., et al., Assessment of sex differences in pharmacokinetics and pharmacodynamics of amlodipine in a bioequivalence study. Pharmacological Research, 2005. 51(5): p. 445-452.

8. Abdel-Kader, M.S., et al., Quantitative Analysis of Benzyl Isothiocyanate in Salvadora persica Extract and Dental Care Herbal Formulations Using Reversed Phase C18 High-Performance Liquid Chromatography Method. Pharmacognosy Magazine, 2017. 13(51): p. S412-S416.

9. Abdulkareem, A.A., et al., A randomized double-blind clinical trial to evaluate the efficacy of chlorhexidine, antioxidant, and hyaluronic acid mouthwashes in the management of biofilm-induced gingivitis. Int J Dent Hyg, 2020. 18(3): p. 268-277.

10. Abelson, D.C., J. Barton, and I.D. Mandel, The effect of chewing sorbitol-sweetened gum on salivary flow and cemental plaque pH in subjects with low salivary flow. J Clin Dent, 1990. 2(1): p. 3-5.

11. Abelson, D.C. and I.D. Mandel, Comparative study of plaque pH on enamel and cemental surfaces. J Clin Dent, 1990. 2(1): p. 1-2.

12. Ablon, G., A 3-Month, Randomized, Double-Blind, Placebo-Controlled Study Evaluating the Ability of an Extra-Strength Marine Protein Supplement to Promote Hair Growth and Decrease Shedding in Women with Self-Perceived Thinning Hair. Dermatology Research and Practice, 2015. 2015.

13. Abu Bakar, A.H., et al., The Effects of Varying Level of Glucose and Fructose on Brain Activation During Mouth Rinse, in 3rd International Conference on Movement, Health and Exercise: Engineering Olympic Success: From Theory to Practice, F. Ibrahim, et al., Editors. 2017. p. 111-116.

14. Adams, M.R., et al., ORAL L-ARGININE INHIBITS PLATELET-AGGREGATION BUT DOES NOT ENHANCE ENDOTHELIUM-DEPENDENT DILATION IN HEALTHY-YOUNG MEN. Journal of the American College of Cardiology, 1995. 26(4): p. 1054-1061.

15. Addai, F.K., I.K. Nuamah, and G.E. Parkins, Brief chewing of Garcinia manii stick reverses reduced saliva pH after a glucose rinse. Med Sci Monit, 2002. 8(11): p. Cr746-50.

16. Addy, M., et al., An evaluation of video instruction for an electric toothbrush - Comparative single-brushing cross-over study. Journal of Clinical Periodontology, 1999. 26(5): p. 289-293.

17. Aden, U., C. Jung-Hoffmann, and H. Kuhl, A randomized cross-over study on various hormonal parameters of two triphasic oral contraceptives. Contraception, 1998. 58(2): p. 75-81.

18. Agustina, F., Z.M. Sofro, and G. Partadiredja, Subchronic Administration of High-Dose Sodium Fluoride Causes Deficits in Cerebellar Purkinje Cells But Not Motor Coordination of Rats. Biological trace element research, 2019. 188(2): p. 424-433.

19. Ahldén, M.L. and G. Frostell, Variation in pH of plaque after a mouth rinse with a saturated solution of mannitol. Odontol Revy, 1975. 26(1): p. 1-5.

20. Ahmed, S.F., et al., Randomized, crossover comparison study of the short-term effect of oral testosterone undecanoate and intramuscular testosterone depot on linear growth and serum bone alkaline phosphatase. Journal of Pediatric Endocrinology & Metabolism, 2004. 17(7): p. 941-950.

21. Ahmetov, II, et al., The ACTN3 R577X polymorphism in Russian endurance athletes. British Journal of Sports Medicine, 2010. 44(9): p. 649-652.

22. Ahmetov, I.I., The ACTN3 R577X polymorphism in Russian endurance athletes. British Journal of Sports Medicine, 2010. 44(9): p. 649-652.

23. Ahmetov, I.I., et al., The ACTN3 R577X polymorphism in Russian endurance athletes. British journal of sports medicine, 2010. 44(9): p. 649-52.

24. Aibibai, Y., W. Hamulati, and L.J. Zhong, [Effects of xipayi mouth rinse on the DNA synthesis and change of cell cycles of human gingival fibroblast induced by lipopolysaccharide]. Zhong Nan Da Xue Xue Bao Yi Xue Ban, 2006. 31(4): p. 483-6.

25. Akhmetov, I.I., et al., [The association of gene polymorphisms with the muscle fiber type composition]. Rossiiskii fiziologicheskii zhurnal imeni I.M. Sechenova, 2006. 92(7): p. 883-8.

26. Al-Ak'hali, M.S., Performance of Dental Students and Interns in the Motivation and Verbal Delivery of Oral Hygiene Instructions in Dental Practice. The journal of contemporary dental practice, 2020. 21(5): p. 532-538.

27. Al-Nimer, M.S.M., Effects of meloxicam and rofecoxib on psychomotor performance: A randomized, double-blind, placebo-controlled cross-over study. Indian Journal of Pharmacology, 2007. 39(6): p. 291-293.

28. Albersen, M., et al., Pentoxifylline Promotes Recovery of Erectile Function in a Rat Model of Postprostatectomy Erectile Dysfunction. European Urology, 2011. 59(2): p. 286-296.

29. Ali, A., et al., Carbohydrate mouth rinsing has no effect on power output during cycling in a glycogen-reduced state. Journal of the International Society of Sports Nutrition, 2016. 13: p. 1-10.

30. Ali, A., et al., Effect of mouth rinsing and ingestion of carbohydrate solutions on mood and perceptual responses during exercise. Journal of the International Society of Sports Nutrition, 2017. 14: p. 1-10.

31. Ali, A., et al., Effect of mouth rinsing and ingestion of carbohydrate solutions on mood and perceptual responses during exercise. Journal of the International Society of Sports Nutrition, 2017. 14: p. 4.

32. Ali, A., et al., Carbohydrate mouth rinsing has no effect on power output during cycling in a glycogen-reduced state. J Int Soc Sports Nutr, 2016. 13: p. 19.

33. Ali, A., et al., Carbohydrate mouth rinsing has no effect on power output during cycling in a glycogen-reduced state. Journal of the International Society of Sports Nutrition, 2016. 13: p. 19.

34. Alioth, C., et al., APPLICATION OF DUAL RADIOTELEMETRIC TECHNIQUE IN STUDYING DRUG-DRUG INTERACTION BETWEEN DICLOFENAC SODIUM AND RANITIDINE HCL IN VOLUNTEERS. Pharmaceutical Research, 1993. 10(11): p. 1688-1692.

35. Alkhudhairy, F., et al., Effect of Er,Cr:YSGG Laser and Ascorbic Acid on the Bond Strength and Microleakage of Bleached Enamel Surface. Photomed Laser Surg, 2018. 36(8): p. 431-438.

36. Allen, C.M., et al., Changes in plasma and oral mucosal lycopene isomer concentrations in healthy adults consuming standard servings of processed tomato products. Nutrition and Cancer-an International Journal, 2003. 47(1): p. 48-56.

37. Almqvist, H. and F. Lagerlof, Effect of intermittent delivery of fluoride to solution on root hard-tissue de- and remineralization measured by 125I absorptiometry. Journal of dental research, 1993. 72(12): p. 1593-8.

38. Altenburger, M.J., et al., The evaluation of fluorescence changes after application of casein phosphopeptides (CPP) and amorphous calcium phosphate (ACP) on early carious lesions. American Journal of Dentistry, 2010. 23(4): p. 188-192.

39. Amar, M.J.A., et al., Randomized double blind clinical trial on the effect of oral alpha-cyclodextrin on serum lipids. Lipids in Health and Disease, 2016. 15.

40. Amini, H., et al., Lack of bioequivalence between two aciclovir tablets in healthy subjects. Clinical Drug Investigation, 2008. 28(1): p. 47-53.

41. Ammar, H.O., et al., Design and In Vitro/In Vivo Evaluation of Ultra-Thin Mucoadhesive Buccal Film Containing Fluticasone Propionate. AAPS PharmSciTech, 2017. 18(1): p. 93-103.

42. Andersson, H., et al., The effect of carbohydrate mouth rinse on a 30-minute arm cranking performance. Comparative Exercise Physiology, 2016. 12(1): p. 41-47.

43. Andries, A., et al., Changes in IGF-I, urinary free cortisol and adipokines during dronabinol therapy in anorexia nervosa: Results from a randomised, controlled trial. Growth Hormone & Igf Research, 2015. 25(5): p. 247-252.

44. Anlamlert, W. and P. Sermsappasuk, Pomegranate Juice does not Affect the Bioavailability of Cyclosporine in Healthy Thai Volunteers. Current Clinical Pharmacology, 2020. 15(2): p. 145-151.

45. Anlamlert, W., et al., Pomelo Enhances Cyclosporine Bioavailability in Healthy Male Thai Volunteers. Journal of Clinical Pharmacology, 2015. 55(4): p. 377-383.

46. Anthonappa, R.P., N.M. King, and A.B.M. Rabie, Evaluation of the long-term storage stability of saliva as a source of human DNA. Clinical Oral Investigations, 2013. 17(7): p. 1719-1725.

47. Aqil, M., et al., Comparative bioavailability of metoprolol tartrate after oral and transidermal administration in healthy male volunteers. Clinical Drug Investigation, 2007. 27(12): p. 833-839.

48. Aranibar Quiroz, E.M., P. Lingström, and D. Birkhed, Influence of short-term sucrose exposure on plaque acidogenicity and cariogenic microflora in individuals with different levels of mutans streptococci. Caries Res, 2003. 37(1): p. 51-7.

49. Archer, D.F., et al., Pharmacokinetics and adhesion of the Agile transdermal contraceptive patch (AG200-15) during daily exposure to external conditions of heat, humidity and exercise. Contraception, 2013. 87(2): p. 212-219.

50. Archer, T.M., et al., Pharmacodynamic Monitoring of Canine T-Cell Cytokine Responses to Oral Cyclosporine. Journal of Veterinary Internal Medicine, 2011. 25(6): p. 1391-1397.

51. Arlt, W., F. Callies, and B. Allolio, DHEA replacement in women with adrenal insufficiency pharmacokinetics, bioconversion and clinical effects on well-being, sexuality and cognition. Endocrine Research, 2000. 26(4): p. 505-511.

52. Arnaoutis, G., et al., Small Amount of Water Ingestion but not Mouth Rinse Improves Exercise Performance in Dehydrated Athletes. Medicine and Science in Sports and Exercise, 2010. 42(5): p. 112-112.

53. Arnaoutis, G., et al., Water ingestion improves performance compared with mouth rinse in dehydrated subjects. Medicine and science in sports and exercise, 2012. 44(1): p. 175-9.

54. Arthur, R.A., et al., Genotypic and phenotypic analysis of S. mutans isolated from dental biofilms formed in vivo under high cariogenic conditions. Braz Dent J, 2011. 22(4): p. 267-74.

55. Arunakul, M., et al., Efficacy of xylitol and fluoride mouthrinses on salivary Mutans streptococci. Asian Pac J Trop Biomed, 2011. 1(6): p. 488-90.

56. Arweiler, N.B., et al., Efficacy of an amine fluoride-triclosan mouthrinse as compared to the individual active ingredients. Journal of clinical periodontology, 2003. 30(3): p. 192-6.

57. Arweiler, N.B., et al., Differences in efficacy of two commercial 0.2% chlorhexidine mouthrinse solutions: a 4-day plaque re-growth study. Journal of clinical periodontology, 2006. 33(5): p. 334-9.

58. Arweiler, N.B., et al., Antibacterial Effect and Substantivity of Toothpaste Slurries In Vivo. Oral health & preventive dentistry, 2018. 16(2): p. 175-181.

59. Arweiler, N.B., et al., Effect of food preservatives on in situ biofilm formation. Clinical Oral Investigations, 2008. 12(3): p. 203-208.

60. Arweiler, N.B., L. Netuschil, and E. Reich, Alcohol-free mouthrinse solutions to reduce supragingival plaque regrowth and vitality. A controlled clinical study. Journal of clinical periodontology, 2001. 28(2): p. 168-74.

61. Arweiler, N.B., L. Netuschil, and E. Reich, Alcohol-free mouthrinse solutions to reduce supragingival plaque regrowth and vitality - A controlled clinical study. Journal of Clinical Periodontology, 2001. 28(2): p. 168-174.

62. Arya, S.C., AIDS: the plateau? Health for the millions, 1991. 17(4): p. 12-4.

63. Ashkenazi, M., M. Bidoosi, and L. Levin, Factors associated with reduced compliance of children to dental preventive measures. Odontology, 2012. 100(2): p. 241-8.

64. Ashkenazi, M., O. Kessler-Baruch, and L. Levin, Oral hygiene instructions provided by dental hygienists: Results from a self-report cohort study and a suggested protocol for oral hygiene education. Quintessence International, 2014. 45(3): p. 265-269.

65. Ashworth, A., et al., Dietary intake of inorganic nitrate in vegetarians and omnivores and its impact on blood pressure, resting metabolic rate and the oral microbiome. Free radical biology & medicine, 2019. 138: p. 63-72.

66. Ashworth, E.T., J.D. Cotter, and A.E. Kilding, Impact of elevated core temperature on cognition in hot environments within a military context. European Journal of Applied Physiology, 2021. 121(4): p. 1061-1071.

67. Asiri, Y.A., et al., Comparative bioavailability study of cefixime (equivalent to 100 mg/5 ml) suspension (Winex vs Suprax) in healthy male volunteers. International Journal of Clinical Pharmacology and Therapeutics, 2005. 43(10): p. 499-504.

68. Asmussen, E. and A. Peutzfeldt, Bonding of dual-curing resin cements to dentin. J Adhes Dent, 2006. 8(5): p. 299-304.

69. Ataide-Silva, T., et al., CHO Mouth Rinse Ameliorates Neuromuscular Response with Lower Endogenous CHO Stores. Medicine & Science in Sports & Exercise, 2016. 48(9): p. 1810-1820.

70. Ataide-Silva, T., et al., CHO Mouth Rinse Ameliorates Neuromuscular Response with Lower Endogenous CHO Stores. Medicine and science in sports and exercise, 2016. 48(9): p. 1810-20.

71. Atkins, P.C., et al., PRODUCTS OF ARACHIDONIC-ACID METABOLISM AND THE EFFECTS OF CYCLOOXYGENASE INHIBITION ON ONGOING CUTANEOUS ALLERGIC REACTIONS IN HUMAN-BEINGS. Journal of Allergy and Clinical Immunology, 1995. 95(3): p. 742-747.

72. Attin, T., et al., Anti-erosive effect of a self-assembling peptide gel. Swiss dental journal, 2017. 127(10): p. 857-864.

73. Aweeka, F.T., et al., PHARMACOKINETICS OF ORALLY AND INTRAVENOUSLY ADMINISTERED CYCLOSPORINE IN PRE-KIDNEY TRANSPLANT PATIENTS. Journal of Clinical Pharmacology, 1994. 34(1): p. 60-67.

74. Axelsson, P. and J. Lindhe, Efficacy of mouthrinses in inhibiting dental plaque and gingivitis in man. Journal of clinical periodontology, 1987. 14(4): p. 205-12.

75. Aydin, M., et al., Instant and freshness effect of mouth rinses on type 1 (oral) halitosis. Acta Odontol Latinoam, 2019. 32(2): p. 79-87.

76. Aydinyurt, H.S., et al., Evaluation of biochemical and clinical effects of hyaluronic acid on non-surgical periodontal treatment: a randomized controlled trial. Ir J Med Sci, 2020. 189(4): p. 1485-1494.

77. Aydın, Z.U., et al., Effect of chitosan nanoparticle, QMix, and EDTA on TotalFill BC sealers' dentinal tubule penetration: a confocal laser scanning microscopy study. Odontology, 2019. 107(1): p. 64-71.

78. Aykut-Yetkiner, A., T. Attin, and A. Wiegand, Prevention of dentine erosion by brushing with anti-erosive toothpastes. J Dent, 2014. 42(7): p. 856-61.

79. Aykut-Yetkiner, A., A. Wiegand, and T. Attin, The effect of saliva substitutes on enamel erosion in vitro. J Dent, 2014. 42(6): p. 720-5.

80. Baena, E., et al., Effect of Chitosan as a Cross-Linker on Matrix Metalloproteinase Activity and Bond Stability with Different Adhesive Systems. Mar Drugs, 2020. 18(5).

81. Bagley, J.R., et al., Validity of Field Expedient Devices to Assess Core Temperature During Exercise in the Cold. Aviation Space and Environmental Medicine, 2011. 82(12): p. 1098-1103.

82. Bagli, M., et al., Pharmacokinetics of chlorprothixene after single intravenous and oral administration of three galenic preparations. Arzneimittel-Forschung/Drug Research, 1996. 46(3): p. 247-250.

83. Baguet, A., et al., Carnosine loading and washout in human skeletal muscles. Journal of Applied Physiology, 2009. 106(3): p. 837-842.

84. Bailey, S.P., et al., Impact of a Carbohydrate Mouth Rinse on Quadriceps Muscle Function and Corticomotor Excitability. International journal of sports physiology and performance, 2019. 14(7): p. 927-933.

85. Bain, M., et al., Colitis after clindamycin therapy. Journal of oral surgery (American Dental Association : 1965), 1975. 33(4): p. 307-10.

86. Baisini, O., et al., Ursodeoxycholic acid does not affect ethinylestradiol bioavailability in women taking oral contraceptives. European Journal of Clinical Pharmacology, 2004. 60(7): p. 481-487.

87. Baker, A.D., et al., "High Five to Healthy Living": A Health Intervention Program for Youth at an Inner City Community Center. Journal of Community Health, 2012. 37(1): p. 1-9.

88. Baker, L.D., et al., Effects of Growth Hormone-Releasing Hormone on Cognitive Function in Adults With Mild Cognitive Impairment and Healthy Older Adults Results of a Controlled Trial. Archives of Neurology, 2012. 69(11): p. 1420-1429.

89. Bakhtazad, A., et al., Evaluation of the CART peptide expression in morphine sensitization in male rats. European Journal of Pharmacology, 2017. 802: p. 52-59.

90. Balcells, M.E., et al., Rapid molecular detection of pulmonary tuberculosis in HIV-infected patients in Santiago, Chile. International Journal of Tuberculosis and Lung Disease, 2012. 16(10): p. 1349-1353.

91. Ballini, A., et al., Combined sea salt-based oral rinse with xylitol in orthodontic patients: clinical and microbiological study. J Biol Regul Homeost Agents, 2019. 33(1): p. 263-268.

92. Ballini, A., et al., Efficacy of Sea Salt-Based Mouthwash and Xylitol in Improving Oral Hygiene among Adolescent Population: A Pilot Study. Int J Environ Res Public Health, 2020. 18(1).

93. Balloni, S., et al., Cytotoxicity of three commercial mouthrinses on extracellular matrix metabolism and human gingival cell behaviour. Toxicol In Vitro, 2016. 34: p. 88-96.

94. Baltazar-Martins, G. and J. Del Coso, Carbohydrate Mouth Rinse Decreases Time to Complete a Simulated Cycling Time Trial. Frontiers in Nutrition, 2019. 6.

95. Baralic, I., et al., EFFECT OF SENSORY STIMULATION ON SALIVARY IgA SECRETION RATE IN KARATE PLAYERS. Biology of Sport, 2010. 27(4): p. 273-278.

96. Barbé, G., et al., [Vancomycin-resistant enterococci in pediatric hematology: don't panic!]. Pathol Biol (Paris), 1998. 46(6): p. 408-11.

97. Barbério, D., et al., An in vitro study to assess glucose microleakage along fiber posts cemented with 2- and 3-step etch-and-rinse resin adhesive systems. Eur J Prosthodont Restor Dent, 2011. 19(1): p. 33-7.

98. Barbour, M.M., et al., EFFECT OF DIPYRIDAMOLE THERAPY ON MYOCARDIAL-ISCHEMIA IN PATIENTS WITH STABLE ANGINA-PECTORIS RECEIVING CONCURRENT ANTIISCHEMIC THERAPY. American Journal of Cardiology, 1992. 69(5): p. 449-452.

99. Barditch-Crovo, P., et al., The effects of rifampin and rifabutin on the pharmacokinetics and pharmacodynamics of a combination oral contraceptive. Clinical Pharmacology & Therapeutics, 1999. 65(4): p. 428-438.

100. Barker, G., et al., The effects of sucralfate suspension and diphenhydramine syrup plus kaolin-pectin on radiotherapy-induced mucositis. Oral Surg Oral Med Oral Pathol, 1991. 71(3): p. 288-93.

101. Barone, J.A., et al., Food interaction and steady-state pharmacokinetics of itraconazole oral solution in healthy volunteers. Pharmacotherapy, 1998. 18(2): p. 295-301.

102. Bartlett, J.D., J.A. Hawley, and J.P. Morton, Carbohydrate availability and exercise training adaptation: too much of a good thing? European journal of sport science, 2015. 15(1): p. 3-12.

103. Barwood, M.J., et al., Menthol as an Ergogenic Aid for the Tokyo 2021 Olympic Games: An Expert-Led Consensus Statement Using the Modified Delphi Method. Sports medicine (Auckland, N.Z.), 2020. 50(10): p. 1709-1727.

104. Basaraba, C.N., et al., Estimating systemic exposure to levonorgestrel from an oral contraceptive. Contraception, 2017. 95(4): p. 398-404.

105. Bashar, T., et al., Pharmacokinetics and Bioavailability Study of a Prednisolone Tablet as a Single Oral Dose in Bangladeshi Healthy Volunteers. Dose-Response, 2018. 16(3).

106. Bastos-Silva, V.J., et al., Effects of carbohydrate intake on time to exhaustion and anaerobic contribution during supramaximal exercise. Revista De Nutricao-Brazilian Journal of Nutrition, 2016. 29(5): p. 691-697.

107. Bastos-Silva, V.J., et al., Carbohydrate Mouth Rinse Maintains Muscle Electromyographic Activity and Increases Time to Exhaustion during Moderate but not High-Intensity Cycling Exercise. Nutrients, 2016. 8(3).

108. Bastos-Silva, V.J., et al., Carbohydrate Mouth Rinse Maintains Muscle Electromyographic Activity and Increases Time to Exhaustion during Moderate but not High-Intensity Cycling Exercise. Nutrients, 2016. 8(3): p. 49.

109. Bastos-Silva, V.J., et al., Carbohydrate Mouth Rinse Maintains Muscle Electromyographic Activity and Increases Time to Exhaustion during Moderate but not High-Intensity Cycling Exercise. Nutrients, 2016. 8(3): p. 49.

110. Bastos-Silva, V.J., J. Prestes, and A.A.R. Geraldes, Effect of Carbohydrate Mouth Rinse on Training Load Volume in Resistance Exercises. Journal of strength and conditioning research, 2019. 33(6): p. 1653-1657.

111. Bataineh, M.a.F., et al., Impact of carbohydrate mouth rinsing on time to exhaustion during Ramadan: A randomized controlled trial in Jordanian men. European journal of sport science, 2018. 18(3): p. 357-366.

112. Bataineh, M.F., et al., Impact of carbohydrate mouth rinsing on time to exhaustion during Ramadan: A randomized controlled trial in Jordanian men. European Journal of Sport Science, 2018. 18(3): p. 357-366.

113. Bauer, J.E., et al., Obtaining DNA from a geographically dispersed cohort of current and former smokers: use of mail-based mouthwash collection and monetary incentives. Nicotine & tobacco research : official journal of the Society for Research on Nicotine and Tobacco, 2004. 6(3): p. 439-46.

114. Bauer, J.E., et al., Obtaining DNA from a geographically dispersed cohort of current and former smokers: Use of mail-based mouthwash collection and monetary incentives. Nicotine & Tobacco Research, 2004. 6(3): p. 439-446.

115. Bauer, J.G., The index of ADOH: concept of measuring oral self-care functioning in the elderly. Special care in dentistry : official publication of the American Association of Hospital Dentists, the Academy of Dentistry for the Handicapped, and the American Society for Geriatric Dentistry, 2001. 21(2): p. 63-7.

116. Bavaresco Gambassi, B., et al., Carbohydrate mouth rinse improves cycling performance carried out until the volitional exhaustion. Journal of Sports Medicine and Physical Fitness, 2019. 59(1): p. 1-5.

117. Bavaresco Gambassi, B., et al., Carbohydrate mouth rinse improves cycling performance carried out until the volitional exhaustion. The Journal of sports medicine and physical fitness, 2019. 59(1): p. 1-5.

118. Bavaresco Gambassi, B., et al., Carbohydrate mouth rinse improves cycling performance carried out until the volitional exhaustion. J Sports Med Phys Fitness, 2019. 59(1): p. 1-5.

119. Bazzucchi, I., et al., Carbohydrate Mouth Rinsing: Improved Neuromuscular Performance During Isokinetic Fatiguing Exercise. International journal of sports physiology and performance, 2017. 12(8): p. 1031-1038.

120. Beale, D.J., et al., Metabolic Profiling from an Asymptomatic Ferret Model of SARS-CoV-2 Infection. Metabolites, 2021. 11(5).

121. Beaven, C.M., et al., Effects of caffeine and carbohydrate mouth rinses on repeated sprint performance. Applied physiology, nutrition, and metabolism = Physiologie appliquee, nutrition et metabolisme, 2013. 38(6): p. 633-7.

122. Beelen, M., et al., Carbohydrate mouth rinsing in the fed state: lack of enhancement of time-trial performance. Int J Sport Nutr Exerc Metab, 2009. 19(4): p. 400-9.

123. Beelen, M., et al., Carbohydrate mouth rinsing in the fed state: lack of enhancement of time-trial performance. International journal of sport nutrition and exercise metabolism, 2009. 19(4): p. 400-9.

124. Beggrow, E.P., et al., Assessing Scientific Practices Using Machine-Learning Methods: How Closely Do They Match Clinical Interview Performance? Journal of Science Education and Technology, 2014. 23(1): p. 160-182.

125. Behn, D. and M.J. Potter, Sildenafil-mediated reduction in retinal function in heterozygous mice lacking the gamma-subunit of phosphodiesterase. Investigative Ophthalmology & Visual Science, 2001. 42(2): p. 523-527.

126. Bejder, J., et al., Tramadol Does Not Improve Performance or Impair Motor Function in Trained Cyclists. Medicine and Science in Sports and Exercise, 2020. 52(5): p. 1169-1175.

127. Bell, E.B., et al., Green Salad Intake Is Associated with Improved Oral Cancer Survival and Lower Soluble CD44 Levels. Nutrients, 2021. 13(2).

128. Belmonte, C., et al., Evaluation of the Relationship Between Pharmacokinetics and the Safety of Aripiprazole and Its Cardiovascular Effects in Healthy Volunteers. Journal of Clinical Psychopharmacology, 2016. 36(6): p. 608-614.

129. Bemben, M.G., et al., Effects of creatine supplementation on isometric force-time curve characteristics. Medicine and Science in Sports and Exercise, 2001. 33(11): p. 1876-1881.

130. Benevides, R.O.A., et al., Syzygium cumini (L.) Skeels improves metabolic and ovarian parameters in female obese rats with malfunctioning hypothalamus-pituitary-gonadal axis. Journal of Ovarian Research, 2019. 12.

131. Berardi, J.M. and T.N. Ziegenfuss, Effects of ribose supplementation on repeated sprint performance in men. Journal of Strength and Conditioning Research, 2003. 17(1): p. 47-52.

132. Bergen, A.W., et al., Comparison of yield and genotyping performance of multiple displacement amplification and OmniPlex whole genome amplified DNA generated from multiple DNA sources. Human mutation, 2005. 26(3): p. 262-70.

133. Bergler, R., [Psychology of Hygiene: Rsult of a Comparative Study 1968/1976 (author's transl)]. Psychologie der Sauberkeit: Ergebnis einer Vergleichsuntersuchung 1968/1976, 1976. 163(1-4): p. 268-310.

134. Bergler, R., STUDIES TO THE PSYCHOLOGICAL SITUATION OF HOME CARE PATIENTS IN THE FEDERAL-REPUBLIC-OF-GERMANY. Zentralblatt Fur Hygiene Und Umweltmedizin, 1993. 194(1-2): p. 33-79.

135. Beringer, P.M., et al., Lack of effect of P-glycoprotein inhibition on renal clearance of dicloxacillin in patients with cystic fibrosis. Pharmacotherapy, 2008. 28(7): p. 883-894.

136. Berktas, B., et al., Mucormycosis Presented with Facial Pain in a Renal Transplant Patient: A Case Report. Transplantation Proceedings, 2019. 51(7): p. 2498-2500.

137. Berlin-Broner, Y., L. Levin, and M. Ashkenazi, Awareness of orthodontists regarding oral hygiene performance during active orthodontic treatment. European journal of paediatric dentistry, 2012. 13(3): p. 187-91.

138. Bertera, E.M., Storytelling Slide Shows to Improve Diabetes and High Blood Pressure Knowledge and Self-Efficacy: Three-Year Results Among Community Dwelling Older African Americans. Educational Gerontology, 2014. 40(11): p. 785-800.

139. Bescos, R., et al., Effects of Chlorhexidine mouthwash on the oral microbiome. Scientific reports, 2020. 10(1): p. 5254.

140. Bescos, R., et al., Effects of Dietary L-Arginine Intake on Cardiorespiratory and Metabolic Adaptation in Athletes. International Journal of Sport Nutrition and Exercise Metabolism, 2009. 19(4): p. 355-365.

141. Best, R., P.S. Maulder, and N. Berger, Perceptual and Physiological Responses to Carbohydrate and Menthol Mouth-Swilling Solutions: A Repeated Measures Cross-Over Preliminary Trial. Beverages, 2021. 7(1).

142. Best, R., et al., Can taste be ergogenic? European Journal of Nutrition, 2021. 60(1): p. 45-54.

143. Best, R., et al., The Development of a Menthol Solution for Use during Sport and Exercise. Beverages, 2018. 4(2).

144. Betteridge, S., et al., No effect of acute beetroot juice ingestion on oxygen consumption, glucose kinetics, or skeletal muscle metabolism during submaximal exercise in males. Journal of applied physiology (Bethesda, Md. : 1985), 2016. 120(4): p. 391-8.

145. Beyari, M.M., et al., Inter- and intra-person cytomegalovirus infection in Malawian families. J Med Virol, 2005. 75(4): p. 575-82.

146. Bidra, A.S., et al., Antimicrobial efficacy of oral topical agents on microorganisms associated with radiated head and neck cancer patients: an in vitro study. Quintessence Int, 2011. 42(4): p. 307-15.

147. Biederbick, W., et al., Caffeine in saliva after peroral intake: Early sample collection as a possible source of error. Therapeutic Drug Monitoring, 1997. 19(5): p. 521-524.

148. Bienek, D.R. and J.J. Filliben, Risk assessment and sensitivity meta-analysis of alveolar osteitis occurrence in oral contraceptive users. Journal of the American Dental Association, 2016. 147(6): p. 394-404.

149. Birkhed, D. and G. Fuchs, Influence of sugar content in soft bread on pH of human dental plaque. Acta Odontol Scand, 1975. 33(2): p. 59-66.

150. Birkhed, D., K. Wickholm, and G. Frostell, Degradation of maltose and starch by human saliva and by supernatants of dental plaque material. Odontol Revy, 1975. 26(1): p. 7-16.

151. Bisson, C., et al., PHARMACOKINETICS AND PHARMACODYNAMICS OF RANITIDINE AND FAMOTIDINE IN HEALTHY ELDERLY SUBJECTS - A DOUBLE-BLIND, PLACEBO-CONTROLLED COMPARISON. Pharmacotherapy, 1993. 13(1): p. 3-9.

152. Björkman, L. and B. Lind, Factors influencing mercury evaporation rate from dental amalgam fillings. Scand J Dent Res, 1992. 100(6): p. 354-60.

153. Black, C.D., et al., Carbohydrate Mouth Rinsing Does Not Prevent the Decline in Maximal Strength After Fatiguing Exercise. Journal of strength and conditioning research, 2018. 32(9): p. 2466-2473.

154. Blake-Haskins, J.C., J.R. Mellberg, and C. Snyder, Effect of calcium in model plaque on the anticaries activity of fluoride in vitro. Journal of dental research, 1992. 71(8): p. 1482-6.

155. Bloomer, R.J., et al., Impact of Oral Ubiquinol on Blood Oxidative Stress and Exercise Performance. Oxidative Medicine and Cellular Longevity, 2012. 2012.

156. Bloomer, R.J., W.A. Smith, and K.H. Fisher-Wellman, Oxidative Stress in Response to Forearm Ischemia-reperfusion with and without Carnitine Administration. International Journal for Vitamin and Nutrition Research, 2010. 80(1): p. 12-23.

157. Boat, R., et al., Self-control exertion and caffeine mouth rinsing: Effects on cycling time-trial performance. Psychology of Sport and Exercise, 2021. 53.

158. Bogossian, P.M., et al., Effect of carbohydrate mouth rinse on exercise performance in horses. Comparative Exercise Physiology, 2017. 13(2): p. 79-86.

159. Boldry, E.J., et al., Effects of 2-Phenethyl Isothiocyanate on Metabolism of 1,3-Butadiene in Smokers. Cancer Prevention Research, 2020. 13(1): p. 91-99.

160. Bondonno, C.P., et al., Antibacterial mouthwash blunts oral nitrate reduction and increases blood pressure in treated hypertensive men and women. American journal of hypertension, 2015. 28(5): p. 572-5.

161. Bonifacio, A., et al., French general practitioners' views and experiences of home-made remedies The RGM study. Exercer-La Revue Francophone De Medecine Generale, 2019(151): p. 122-128.

162. Bonnet, F., et al., Consumption of a Carbonated Beverage with High-Intensity Sweeteners Has No Effect on Insulin Sensitivity and Secretion in Nondiabetic Adults. Journal of Nutrition, 2018. 148(8): p. 1293-1299.

163. Bono, A.V. and S. Cuffari, Efficacy and tolerability of tramadol in neoplastic pain: A comparative study with buprenorphine. Drugs, 1997. 53: p. 40-49.

164. Borszcz, F.K. and R.D. de Lucas, Comment on: "Effects of Carbohydrate Mouth Rinse on Cycling Time Trial Performance: A Systematic Review and Meta-analysis" and Subsequent Comment/Author Reply from Li et al. Sports Medicine, 2020. 50(3): p. 629-632.

165. Borthen Svinhufvud, L., A. Heimdahl, and C.E. Nord, Effect of topical administration of vancomycin versus chlorhexidine on alpha-hemolytic streptococci in oral cavity. Oral Surg Oral Med Oral Pathol, 1988. 66(3): p. 304-9.

166. Bortolotti, H., et al., Enxágue bucal com carboidrato: recurso ergogênico capaz de otimizar o desempenho físico. / Carbohydrate mouth rinse: an ergogenic aid able of optimizing the performance. Brazilian Journal of Kineanthropometry & Human Performance, 2011. 13(2): p. 158-161.

167. Bortolotti, H., et al., Mouth rinse with carbohydrate drink and performance: hypothesis of its central action. International SportMed Journal, 2014. 15(1): p. 84-95.

168. Bortolotti, H., et al., Carbohydrate mouth rinse does not improve repeated sprint performance. / Enxágue bucal com carboidrato não melhora o desempenho em sprints repetidos. Brazilian Journal of Kineanthropometry & Human Performance, 2013. 15(6): p. 639-645.

169. Botelho, M.P.J., et al., Rubbing time and bonding performance of one-step adhesives to primary enamel and dentin. J Appl Oral Sci, 2017. 25(5): p. 523-532.

170. Bottiger, Y., et al., Pharmacokinetic interaction between single oral doses of ditiazem and sirolimus in healthy volunteers. Clinical Pharmacology & Therapeutics, 2001. 69(1): p. 32-40.

171. Bowersock, T.L., et al., Prevention of bacteremia in dogs undergoing dental scaling by prior administration of oral clindamycin or chlorhexidine oral rinse. J Vet Dent, 2000. 17(1): p. 11-6.

172. Boyle, N.B., et al., No effects of ingesting or rinsing sucrose on depleted self-control performance. Physiology & behavior, 2016. 154: p. 151-60.

173. Bradshaw, D.J., et al., Creation of oral care flavours to deliver breath-freshening benefits. Oral diseases, 2005. 11 Suppl 1: p. 75-9.

174. Bremme, K., et al., The APC-PCI concentration as an early marker of activation of blood coagulation A study of women on combined oral contraceptives. Thrombosis Research, 2012. 130(4): p. 636-639.

175. Brietzke, C., et al., Carbohydrate Mouth Rinse Mitigates Mental Fatigue Effects on Maximal Incremental Test Performance, but Not in Cortical Alterations. Brain Sci, 2020. 10(8).

176. Brietzke, C., et al., Effects of Carbohydrate Mouth Rinse on Cycling Time Trial Performance: A Systematic Review and Meta-Analysis. Sports medicine (Auckland, N.Z.), 2019. 49(1): p. 57-66.

177. Brietzke, C., et al., Correction to: Effects of Carbohydrate Mouth Rinse on Cycling Time Trial Performance: A Systematic Review and Meta-Analysis. Sports Medicine, 2019. 49(4): p. 645-645.

178. Brietzke, C., et al., Reply to Borszcz & de Lucas: Comment on: "Effects of Carbohydrate Mouth Rinse on Cycling Time Trial Performance: A Systematic Review and Meta-Analysis". Sports Medicine, 2020. 50(3): p. 633-637.

179. Brietzke, C., P.E. Franco-Alvarenga, and F.O. Pires, Reply to Li et al.: Comment on "Effects of Carbohydrate Mouth Rinse on Cycling Time Trial Performance: A Systematic Review and Meta-Analysis". Sports medicine (Auckland, N.Z.), 2019. 49(5): p. 823-825.

180. Brietzke, C., et al., Effects of Carbohydrate Mouth Rinse on Cycling Time Trial Performance: A Systematic Review and Meta-Analysis. Sports Medicine, 2019. 49(1): p. 57-66.

181. Britse, A. and F. Lagerlöf, The diluting effect of saliva on the sucrose concentration in different parts of the human mouth after a mouth-rinse with sucrose. Arch Oral Biol, 1987. 32(10): p. 755-6.

182. Brouns, F., Heat - sweat - dehydration - rehydration: A praxis oriented approach. Journal of Sports Sciences, 1991. 9: p. 143-152.

183. Brouwer, A.F., et al., Multisite HPV infections in the United States (NHANES 2003-2014): An overview and synthesis. Preventive medicine, 2019. 123: p. 288-298.

184. Brown, A.T., et al., In vitro effect of chlorhexidine and amikacin on oral gram-negative bacilli from bone marrow transplant recipients. Oral Surg Oral Med Oral Pathol, 1990. 70(6): p. 715-9.

185. Brown, D.R., et al., Mouth Rinsing With a Pink Non-caloric, Artificially-Sweetened Solution Improves Self-Paced Running Performance and Feelings of Pleasure in Habitually Active Individuals. Frontiers in Nutrition, 2021. 8.

186. Brown, S.A., et al., Oral nutritional supplementation accelerates skin wound healing: A randomized, placebo-controlled, double-arm, crossover study. Plastic and Reconstructive Surgery, 2004. 114(1): p. 237-244.

187. Brudevold, F., et al., Demineralization potential of different concentrations of gelatinized wheat starch. Caries Res, 1988. 22(4): p. 204-9.

188. Brudevold, F., et al., Effect of some salts of calcium, sodium, potassium, and strontium on intra-oral enamel demineralization. J Dent Res, 1985. 64(1): p. 24-7.

189. Bruggeman, B.K., et al., The absorptive effects of orobuccal non-liposomal nano-sized glutathione on blood glutathione parameters in healthy individuals: A pilot study. Plos One, 2019. 14(4).

190. Buckley, D.A., Fragrance ingredient labelling in products on sale in the U.K. The British journal of dermatology, 2007. 157(2): p. 295-300.

191. Buehlmann, M., et al., Highly effective regimen for decolonization of methicillin-resistant Staphylococcus aureus carriers. Infection control and hospital epidemiology, 2008. 29(6): p. 510-6.

192. Bukhari, N.I., et al., Bioequivalence assessment of two enteric-coated aspirin brands, Nu-seals (R) and Loprin (R), after a single oral dose of 150mg in healthy male adults. Therapie, 2005. 60(2): p. 167-173.

193. Burdon, C., et al., Effect of drink temperature on core temperature and endurance cycling performance in warm, humid conditions. Journal of Sports Sciences, 2010. 28(11): p. 1147-1156.

194. Burdon, C.A., et al., The influence of ice slushy on voluntary contraction force following exercise-induced hyperthermia. Applied Physiology Nutrition and Metabolism, 2014. 39(7): p. 781-786.

195. Burdon, C.A., et al., The effect of ice slushy ingestion and mouthwash on thermoregulation and endurance performance in the heat. International journal of sport nutrition and exercise metabolism, 2013. 23(5): p. 458-69.

196. Burdon, C.A., et al., Influence of Beverage Temperature on Exercise Performance in the Heat: A Systematic Review. International Journal of Sport Nutrition and Exercise Metabolism, 2010. 20(2): p. 166-174.

197. Burke, L., et al., Carbohydrates for training and competition. Journal of Sports Sciences, 2011. 29: p. S17-S27.

198. Burke, L.M., et al., Toward a Common Understanding of Diet-Exercise Strategies to Manipulate Fuel Availability for Training and Competition Preparation in Endurance Sport. International Journal of Sport Nutrition and Exercise Metabolism, 2018. 28(5): p. 451-463.

199. Burke, L.M., et al., Carbohydrates for training and competition. J Sports Sci, 2011. 29 Suppl 1: p. S17-27.

200. Burke, L.M., et al., Carbohydrates for training and competition. Journal of sports sciences, 2011. 29 Suppl 1: p. S17-27.

201. Burke, L.M., et al., Contemporary Nutrition Strategies to Optimize Performance in Distance Runners and Race Walkers. International journal of sport nutrition and exercise metabolism, 2019. 29(2): p. 117-129.

202. Burke, L.M., et al., Recovery Antagonized Training at the 2014 Meeting of the American College of Sports Medicine. Sportscience, 2014. 18: p. 8-17.

203. Burke, L.M. and R.J. Maughan, The Governor has a sweet tooth - mouth sensing of nutrients to enhance sports performance. European journal of sport science, 2015. 15(1): p. 29-40.

204. Burke, L.M. and R.J. Maughan, The Governor has a sweet tooth – Mouth sensing of nutrients to enhance sports performance. European Journal of Sport Science, 2015. 15(1): p. 29-40.

205. Burkhardt, O., et al., Effects of enteral feeding on the oral bioavailability of moxifloxacin in healthy volunteers. Clinical Pharmacokinetics, 2005. 44(9): p. 969-976.

206. Burstein, A.H., et al., Absorption of phenytoin from rectal suppositories formulated with a polyethylene glycol base. Pharmacotherapy, 2000. 20(5): p. 562-567.

207. Butler, K., J. Maya, and R.L. Teng, Effect of ticagrelor on pulmonary function in healthy elderly volunteers and asthma or chronic obstructive pulmonary disease patients. Current Medical Research and Opinion, 2013. 29(5): p. 569-577.

208. Caccia, S., et al., MULTIPLE-DOSE PHARMACOKINETICS AND SAFETY OF A POTENTIAL MEMORY-ENHANCING COMPOUND, CL-275,838, IN HEALTHY MALE-VOLUNTEERS. Journal of Clinical Pharmacology, 1994. 34(7): p. 748-753.

209. Caenen, A., et al., An in silico framework to analyze the anisotropic shear wave mechanics in cardiac shear wave elastography. Physics in medicine and biology, 2018. 63(7): p. 075005.

210. Caglar, E., et al., Short-term effect of ice-cream containing Bifidobacterium lactis Bb-12 on the number of salivary mutans streptococci and lactobacilli. Acta Odontologica Scandinavica, 2008. 66(3): p. 154-158.

211. Caglar, E., et al., Effect of yogurt with Bifidobacterium DN-173 010 on salivary mutans streptococci and lactobacilli in young adults. Acta Odontologica Scandinavica, 2005. 63(6): p. 317-320.

212. Campos, M.P.D., et al., Guarana (Paullinia cupana) Improves Fatigue in Breast Cancer Patients Undergoing Systemic Chemotherapy. Journal of Alternative and Complementary Medicine, 2011. 17(6): p. 505-512.

213. Canafax, D.M., et al., Amoxicillin middle ear fluid penetration and pharmacokinetics in children with acute otitis media. Pediatric Infectious Disease Journal, 1998. 17(2): p. 149-156.

214. Cao, B., et al., The antiparasitic clioquinol induces apoptosis in leukemia and myeloma cells by inhibiting histone deacetylase activity. The Journal of biological chemistry, 2013. 288(47): p. 34181-34189.

215. Cao, S.S., et al., Pharmacokinetics and relative bioavailability of a generic amisulpride tablet in healthy Chinese volunteers. International Journal of Clinical Pharmacology and Therapeutics, 2017. 55(10): p. 825-831.

216. Capoluongo, E., et al., Heterogeneity of oral isolates of Candida albicans in HIV-positive patients: correlation between candidal carriage, karyotype and disease stage. Journal of medical microbiology, 2000. 49(11): p. 985-991.

217. Cardenas, K.P.C., et al., Bioequivalence and Tolerability of Ambrisentan: A Pharmacokinetic Study in Mexican Healthy Male Subjects. European Journal of Drug Metabolism and Pharmacokinetics, 2020. 45(5): p. 611-618.

218. Cardona, A.F., et al., Chronic and Severe Non-Lichenoid Oral Ulcers Induced by Nivolumab - Diagnostic and Therapeutic Challenge: A Case Report. Case Reports in Oncology, 2020. 13(1): p. 314-320.

219. Cardoso, F., et al., In situ effect of a proanthocyanidin mouthrinse on dentin subjected to erosion. Journal of applied oral science : revista FOB, 2020. 28: p. e20200051.

220. Carrouel, F., et al., Salivary and Nasal Detection of the SARS-CoV-2 Virus After Antiviral Mouthrinses (BBCovid): A structured summary of a study protocol for a randomised controlled trial. Trials, 2020. 21(1): p. 906.

221. Carrouel, F., et al., Salivary and Nasal Detection of the SARS-CoV-2 Virus After Antiviral Mouthrinses (BBCovid): A structured summary of a study protocol for a randomised controlled trial. Trials, 2020. 21(1).

222. Cartee, L., et al., Evaluation of GM-CSF mouthwash for prevention of chemotherapy-induced mucositis: a randomized, double-blind, dose-ranging study. Cytokine, 1995. 7(5): p. 471-7.

223. Cartee, L., et al., EVALUATION OF GM-CSF MOUTHWASH FOR PREVENTION OF CHEMOTHERAPY-INDUCED MUCOSITIS - A RANDOMIZED, DOUBLE-BLIND, DOSE-RANGING STUDY. Cytokine, 1995. 7(5): p. 471-477.

224. Carter, J.M., A.E. Jeukendrup, and D.A. Jones, The effect of carbohydrate mouth rinse on 1-h cycle time trial performance. Medicine and science in sports and exercise, 2004. 36(12): p. 2107-11.

225. Carvalho, A.A., et al., Influence of different application protocols of universal adhesive system on the clinical behavior of Class I and II restorations of composite resin - a randomized and double-blind controlled clinical trial. Bmc Oral Health, 2019. 19(1).

226. Carvalho, T.S. and A. Lussi, Combined effect of a fluoride-, stannous- and chitosan-containing toothpaste and stannous-containing rinse on the prevention of initial enamel erosion-abrasion. J Dent, 2014. 42(4): p. 450-9.

227. Casella, M., et al., Hormone replacement therapy: One-year follow up of DNA damage. Mutation Research-Genetic Toxicology and Environmental Mutagenesis, 2005. 585(1-2): p. 14-20.

228. Caselli, E., et al., Defining the oral microbiome by whole-genome sequencing and resistome analysis: the complexity of the healthy picture. BMC Microbiol, 2020. 20(1): p. 120.

229. Cavalcanti, D.R. and F.R.X. da Silveira, Alpha lipoic acid in burning mouth syndrome - a randomized double-blind placebo-controlled trial. Journal of Oral Pathology & Medicine, 2009. 38(3): p. 254-261.

230. Cazzola, M., et al., Comparative effects of a two-week treatment with nebivolol and nifedipine in hypertensive patients suffering from COPD. Respiration, 2004. 71(2): p. 159-164.

231. Celik, A.C.T., E. Coban, and H.E. Ulker, Effects of mouthwashes on color stability and surface roughness of three different resin-based composites. Nigerian Journal of Clinical Practice, 2021. 24(4): p. 555-560.

232. Cermak, N. and L. Loon, The Use of Carbohydrates During Exercise as an Ergogenic Aid. Sports Medicine, 2013. 43(11): p. 1139-1155.

233. Chadwick, K.D., et al., Fifty Years of "the Pill": Risk Reduction and Discovery of Benefits Beyond Contraception, Reflections, and Forecast. Toxicological Sciences, 2012. 125(1): p. 2-9.

234. Chai, R.C., et al., A pilot study to compare the detection of HPV-16 biomarkers in salivary oral rinses with tumour p16(INK4a) expression in head and neck squamous cell carcinoma patients. BMC cancer, 2016. 16: p. 178.

235. Chambers, E.S., M.W. Bridge, and D.A. Jones, Carbohydrate sensing in the human mouth: effects on exercise performance and brain activity. J Physiol, 2009. 587(Pt 8): p. 1779-94.

236. Chan, J.T., T.T. Yip, and A.H. Jeske, The role of caffeinated beverages in dental fluorosis. Med Hypotheses, 1990. 33(1): p. 21-2.

237. Chandler, M.C., et al., Carbohydrate mouth rinse has no effects on behavioral or neuroelectric indices of cognition. International Journal of Psychophysiology, 2020. 151: p. 49-58.

238. Chandna, P., N. Srivastava, and S. Ali, Remineralizing Agents: The Next Frontier. Curr Clin Pharmacol, 2016. 11(3): p. 211-220.

239. Chang, A.Y.C., et al., Phase II study of neo-adjuvant chemotherapy for locally advanced gastric cancer. Bmj Open Gastroenterology, 2016. 3(1).

240. Chang, H., J. Blackburn, and M. Grootveld, Chemometric analysis of the consumption of oral rinse chlorite (ClO2-) by human salivary biomolecules. Clinical oral investigations, 2013. 17(9): p. 2065-78.

241. Chang, T., L.Z. Benet, and M.F. Hebert, The effect of water-soluble vitamin E On cyclosporine pharmacokinetics in healthy volunteers. Clinical Pharmacology & Therapeutics, 1996. 59(3): p. 297-303.

242. Chapple, C., et al., A pooled analysis of three phase III studies to investigate the efficacy, tolerability and safety of darifenacin, a muscarinic M-3 selective receptor antagonist, in the treatment of overactive bladder. Bju International, 2005. 95(7): p. 993-1001.

243. Charone, S., et al., The effect of mouthwashes containing biguanides on the progression of erosion in dentin. BMC oral health, 2014. 14: p. 131.

244. Charone, S., et al., The effect of mouthwashes containing biguanides on the progression of erosion in dentin. Bmc Oral Health, 2014. 14.

245. Chavan, S.D., N.L. Shetty, and M. Kanuri, Comparative evaluation of garlic extract mouthwash and chlorhexidine mouthwash on salivary Streptococcus mutans count - an in vitro study. Oral Health Prev Dent, 2010. 8(4): p. 369-74.

246. Chavez, P.R., et al., Performance evaluation of four point-of-care HIV tests using unprocessed specimens. Journal of Clinical Virology, 2020. 124.

247. Chavez-Teyes, L., G. Castaneda-Hernandez, and F.J. Flores-Murrieta, Pharmacokinetics of midazolam in Mexicans - Evidence for interethnic variability. Clinical Drug Investigation, 1999. 17(3): p. 233-239.

248. Che Muhamed, A.M., et al., Mouth rinsing improves cycling endurance performance during Ramadan fasting in a hot humid environment. Applied physiology, nutrition, and metabolism = Physiologie appliquee, nutrition et metabolisme, 2014. 39(4): p. 458-64.

249. Chen, A.M., et al., Quality of Life Among Long-Term Survivors of Head and Neck Cancer Treated by Intensity-Modulated Radiotherapy. Jama Otolaryngology-Head & Neck Surgery, 2014. 140(2): p. 129-133.

250. Chen, C.J., et al., A school-based fluoride mouth rinsing programme in Sarawak: a 3-year field study. Community Dent Oral Epidemiol, 2010. 38(4): p. 310-4.

251. Chen, C.N., et al., An in vitro study on restoring bond strength of a GIC to saliva contaminated enamel under unrinse condition. J Dent, 2002. 30(5-6): p. 189-94.

252. Chen, D., et al., Evaluation of Gabapentin Enacarbil on Cardiac Repolarization: A Randomized, Double-Blind, Placeboand Active-Controlled, Crossover Thorough QT/QTc Study in Healthy Adults. Clinical Therapeutics, 2012. 34(2): p. 351-362.

253. Chen, G., et al., Pharmacokinetics, Safety, and Bioequivalence of Two Empagliflozin Formulations after Single Oral Administration under Fasting and Fed Conditions in Healthy Chinese Subjects: An Open-label Randomized Single-dose Two-sequence, Two-treatment, Two-period Crossover Study. Pharmacotherapy, 2020. 40(7): p. 623-631.

254. Chen, J., et al., Bioequivalence Studies of 2 Oral Cefaclor Capsule Formulations in Chinese Healthy Subjects. Arzneimittelforschung-Drug Research, 2012. 62(3): p. 134-137.

255. Chen, J.W., et al., Effects of short-term treatment of Nicorandil on exercise-induced myocardial ischemia and abnormal cardiac autonomic activity in microvascular angina. American Journal of Cardiology, 1997. 80(1): p. 32-38.

256. Chen, Y., et al., Genotyping as a Key Element of Sample Size Optimization in Bioequivalence of Risperidone Tablets. European Journal of Drug Metabolism and Pharmacokinetics, 2018. 43(4): p. 431-439.

257. Chen, Y., et al., Effect of a traditional Chinese medicine Liu Wei Di Huang Wan on the activities of CYP2C19, CYP2D6 and CYP3A4 in healthy volunteers. Xenobiotica, 2012. 42(6): p. 596-602.

258. Cheng, I.S., et al., Oral hydroxycitrate supplementation enhances glycogen synthesis in exercised human skeletal muscle. British Journal of Nutrition, 2012. 107(7): p. 1048-1055.

259. Cherif, A., et al., Repeated-sprints exercise in daylight fasting: carbohydrate mouth rinsing does not affect sprint and reaction time performance. Biology of Sport, 2018. 35(3): p. 237-244.

260. Cheung, S.S., et al., Separate and combined effects of dehydration and thirst sensation on exercise performance in the heat. Scandinavian journal of medicine & science in sports, 2015. 25 Suppl 1: p. 104-11.

261. Chikandiwa, A., et al., Oropharyngeal HPV infection: prevalence and sampling methods among HIV-infected men in South Africa. International journal of STD & AIDS, 2018. 29(8): p. 776-780.

262. Chin, M.Y.H., et al., Fluoride release and cariostatic potential of orthodontic adhesives with and without daily fluoride rinsing. American Journal of Orthodontics and Dentofacial Orthopedics, 2009. 136(4): p. 547-553.

263. Chin, M.Y.H., et al., Fluoride release and cariostatic potential of orthodontic adhesives with and without daily fluoride rinsing. American journal of orthodontics and dentofacial orthopedics : official publication of the American Association of Orthodontists, its constituent societies, and the American Board of Orthodontics, 2009. 136(4): p. 547-53.

264. Chirinos, J.A., et al., Arterial pulsatile hemodynamic load induced by isometric exercise strongly predicts left ventricular mass in hypertension. American journal of physiology. Heart and circulatory physiology, 2010. 298(2): p. H320-30.

265. Chirinos, J.A., et al., Arterial pulsatile hemodynamic load induced by isometric exercise strongly predicts left ventricular mass in hypertension. American Journal of Physiology-Heart and Circulatory Physiology, 2010. 298(2): p. H320-H330.

266. Choi, E.K., et al., Ketoprofen inhibits expression of inflammatory mediators in human dental pulp cells. J Endod, 2013. 39(6): p. 764-7.

267. Choi, H.M., et al., Associations Among Oral Hygiene Behavior and Hypertension Prevalence and Control: The 2008 to 2010 Korea National Health and Nutrition Examination Survey. Journal of periodontology, 2015. 86(7): p. 866-73.

268. Choi, H.Y., et al., Effects of Ketoconazole on the Pharmacokinetic Properties of CG100649, A Novel NSAID: A Randomized, Open-Label Crossover Study in Healthy Korean Male Volunteers. Clinical Therapeutics, 2014. 36(1): p. 115-125.

269. Chong, E., K.J. Guelfi, and P.A. Fournier, Effect of a carbohydrate mouth rinse on maximal sprint performance in competitive male cyclists. Journal of science and medicine in sport, 2011. 14(2): p. 162-7.

270. Chong, E., K.J. Guelfi, and P.A. Fournier, Combined glucose ingestion and mouth rinsing improves sprint cycling performance. International journal of sport nutrition and exercise metabolism, 2014. 24(6): p. 605-12.

271. Chong, E., K.J. Guelti, and P.A. Fournier, Combined Glucose Ingestion and Mouth Rinsing Improves Sprint Cycling Performance. International Journal of Sport Nutrition and Exercise Metabolism, 2014. 24(6): p. 605-612.

272. Chow, L.C., et al., Remineralization effects of a two-solution fluoride mouthrinse: an in situ study. Journal of dental research, 2000. 79(4): p. 991-5.

273. Christiansen, J.J., et al., Dehydroepiandrosterone substitution in female adrenal failure: no impact on endothelial function and cardiovascular parameters despite normalization of androgen status. Clinical Endocrinology, 2007. 66(3): p. 426-433.

274. Chronopoulou, L., et al., Chitosan based nanoparticles functionalized with peptidomimetic derivatives for oral drug delivery. N Biotechnol, 2016. 33(1): p. 23-31.

275. Chryssanthopoulos, C., et al., Carbohydrate mouth rinse does not affect performance during a 60-min running race in women. Journal of sports sciences, 2018. 36(7): p. 824-833.

276. Chryssanthopoulos, C., et al., Carbohydrate mouth rinse does not affect performance during a 60-min running race in women. Journal of Sports Sciences, 2018. 36(7): p. 824-833.

277. Chuckravanen, D., et al., Review of exercise-induced physiological control models to explain the development of fatigue to improve sports performance and future trend. Science & Sports, 2019. 34(3): p. 131-140.

278. Chung, J.H., et al., Associations Between Periodontitis and Chronic Obstructive Pulmonary Disease: The 2010 to 2012 Korean National Health and Nutrition Examination Survey. Journal of periodontology, 2016. 87(8): p. 864-71.

279. Chung, J.H., et al., Associations Between Periodontitis and Chronic Obstructive Pulmonary Disease: The 2010 to 2012 Korean National Health and Nutrition Examination Survey. Journal of Periodontology, 2016. 87(8): p. 864-871.

280. Church, J.A., et al., The Impact of Improved Water, Sanitation, and Hygiene on Oral Rotavirus Vaccine Immunogenicity in Zimbabwean Infants: Substudy of a Cluster-randomized Trial. Clinical Infectious Diseases, 2019. 69(12): p. 2074-2081.

281. Cidon, E.U., Chemotherapy induced oral mucositis: prevention is possible. Chinese clinical oncology, 2018. 7(1): p. 6.

282. Clark, T.P., et al., The steady-state pharmacokinetics and bioequivalence of carprofen administered orally and subcutaneously in dogs. Journal of Veterinary Pharmacology and Therapeutics, 2003. 26(3): p. 187-192.

283. Clarke, N.D., et al., Carbohydrate mouth rinse improves morning high-intensity exercise performance. European journal of sport science, 2017. 17(8): p. 955-963.

284. Clarke, N.D., E. Kornilios, and D.L. Richardson, Carbohydrate and Caffeine Mouth Rinses Do Not Affect Maximum Strength and Muscular Endurance Performance. Journal of strength and conditioning research, 2015. 29(10): p. 2926-31.

285. Clarke, N.D., et al., No Dose-Response Effect of Carbohydrate Mouth Rinse Concentration on 5-km Running Performance in Recreational Athletes. Journal of strength and conditioning research, 2017. 31(3): p. 715-720.

286. Claydon, N., et al., Comparative professional plaque removal study using 8 branded toothbrushes. Journal of Clinical Periodontology, 2002. 29(4): p. 310-316.

287. Claydon, N.C.A., et al., Clinical study to compare the effectiveness of a test whitening toothpaste with a commercial whitening toothpaste at inhibiting dental stain. Journal of clinical periodontology, 2004. 31(12): p. 1088-91.

288. Cliff, M.A. and B.G. Green, Sensitization and desensitization to capsaicin and menthol in the oral cavity: interactions and individual differences. Physiol Behav, 1996. 59(3): p. 487-94.

289. Close, G.L., A.M. Kasper, and J.P. Morton, From Paper to Podium: Quantifying the Translational Potential of Performance Nutrition Research. Sports Medicine, 2019. 49: p. S25-S37.

290. Cocco, F., et al., The strip method and the microelectrode technique in assessing dental plaque pH. Minerva Stomatol, 2017. 66(6): p. 241-247.

291. Cohen, E.R., et al., CD44 and associated markers in oral rinses and tissues from oral and oropharyngeal cancer patients. Oral Oncol, 2020. 106: p. 104720.

292. Coll, S., et al., Elimination profiles of betamethasone after different administration routes: Evaluation of the reporting level and washout periods to ensure safe therapeutic administrations. Drug Testing and Analysis, 2021. 13(2): p. 348-359.

293. Coll, S., et al., Elimination profiles of prednisone and prednisolone after different administration routes: Evaluation of the reporting level and washout periods to ensure safe therapeutic administrations. Drug Testing and Analysis, 2021. 13(3): p. 571-582.

294. Contreras, J.C.Z., et al., Physical exercise on markers of oxidative stress in saliva of sedentary postmenopausal women. Viref-Revista De Educacion Fisica, 2020. 9(4): p. 109-122.

295. Cooper, S., et al., Effect of mouth taping at night on asthma control - A randomised single-blind crossover study. Respiratory Medicine, 2009. 103(6): p. 813-819.

296. Correia-Oliveira, C., et al., Strategies of Dietary Carbohydrate Manipulation and Their Effects on Performance in Cycling Time Trials. Sports Medicine, 2013. 43(8): p. 707-719.

297. Correia-Oliveira, C., et al., Strategies of Dietary Carbohydrate Manipulation and Their Effects on Performance in Cycling Time Trials. Sports Medicine, 2013. 43(8): p. 707-719.

298. Correia-Oliveira, C.R., et al., Strategies of dietary carbohydrate manipulation and their effects on performance in cycling time trials. Sports Med, 2013. 43(8): p. 707-19.

299. Correia-Oliveira, C.R., et al., Strategies of dietary carbohydrate manipulation and their effects on performance in cycling time trials. Sports medicine (Auckland, N.Z.), 2013. 43(8): p. 707-19.

300. Cortellini, P., et al., Chlorhexidine with an anti discoloration system after periodontal flap surgery: a cross-over, randomized, triple-blind clinical trial. J Clin Periodontol, 2008. 35(7): p. 614-20.

301. Costa, A., et al., Effects of Oral Administration of Alprazolam and Lorazepam as Hypnotics on Cardiovascular Parameters in Hypertensive Patients. Journal of Clinical Psychopharmacology, 2021. 41(2): p. 191-195.

302. Costanzo, A., et al., A Fatty Acid Mouth Rinse Decreases Self-Reported Hunger and Increases Self-Reported Fullness in Healthy Australian Adults: A Randomized Cross-Over Trial. Nutrients, 2020. 12(3).

303. Cotrim, A.P., et al., Kinetics of tempol for prevention of xerostomia following head and neck irradiation in a mouse model. Clinical cancer research : an official journal of the American Association for Cancer Research, 2005. 11(20): p. 7564-8.

304. Cottin, F., et al., Effect of oral glucocorticoid intake on autonomic cardiovascular control. Springerplus, 2015. 4.

305. Cox, G., CARBOHYDRATE SUPPLEMENTATION FOR RUNNING PERFORMANCE. Run for Your Life: R4YL, 2014(54): p. 22-23.

306. Cramer, M.N., M.W. Thompson, and J.D. Periard, Thermal and Cardiovascular Strain Mitigate the Potential Benefit of Carbohydrate Mouth Rinse During Self-Paced Exercise in the Heat. Frontiers in Physiology, 2015. 6.

307. Crivaro, M., et al., Mild arterial hypertension and impaired glucose tolerance: Short term effects of manidipine hydrochloride. Advances in Therapy, 1996. 13(6): p. 365-372.

308. Crofton, P.M., et al., Physiological Versus Standard Sex Steroid Replacement in Young Women With Premature Ovarian Failure: Effects on Bone Mass Acquisition and Turnover EDITORIAL COMMENT. Obstetrical & Gynecological Survey, 2011. 66(6): p. 348-350.

309. Cross, L.J., et al., Serum itraconazole concentrations and clinical responses in Candida-associated denture stomatitis patients treated with itraconazole solution and itraconazole capsules. The Journal of antimicrobial chemotherapy, 2000. 45(1): p. 95-9.

310. Cross, L.J., et al., Evaluation of the recurrence of denture stomatitis and Candida colonization in a small group of patients who received itraconazole. Oral Surgery Oral Medicine Oral Pathology Oral Radiology and Endodontics, 2004. 97(3): p. 351-358.

311. Crossner, C.G., Variation in human oral lactobacilli following a change in sugar intake. Scand J Dent Res, 1984. 92(3): p. 204-10.

312. Cundy, K.C., et al., ORAL BIOAVAILABILITY OF THE ANTIRETROVIRAL AGENT 9-(2-PHOSPHONYLMETHOXYETHYL)ADENINE (PMEA) FROM 3-FORMULATIONS OF THE PRODRUG BIS(PIVALOYLOXYMETHYL)-PMEA IN FASTED MALE CYNOMOLGUS MONKEYS. Pharmaceutical Research, 1994. 11(6): p. 839-843.

313. Cundy, K.C., et al., Pharmacokinetics and bioavailability of the anti-human immunodeficiency virus nucleotide analog 9- (R)-2-(phosphonomethoxy)propyl adenine (PMPA) in dogs. Antimicrobial Agents and Chemotherapy, 1998. 42(3): p. 687-690.

314. Curtis, K.J., et al., Acute Dietary Nitrate Supplementation and Exercise Performance in COPD: A Double-Blind, Placebo-Controlled, Randomised Controlled Pilot Study. Plos One, 2015. 10(12).

315. Cutler, C., et al., Post-exercise hypotension and skeletal muscle oxygenation is regulated by nitrate-reducing activity of oral bacteria. Free radical biology & medicine, 2019. 143: p. 252-259.

316. d'Alcantara, P., S.N. Schiffmann, and S. Swillens, Effect of protein kinase A-induced phosphorylation on the gating mechanism of the brain Na+ channel: model fitting to whole-cell current traces. Biophysical journal, 1999. 77(1): p. 204-16.

317. D'Souza, G., et al., Evaluating the Utility and Prevalence of HPV Biomarkers in Oral Rinses and Serology for HPV-related Oropharyngeal Cancer. Cancer prevention research (Philadelphia, Pa.), 2019. 12(10): p. 689-700.

318. da Silva, L.A.F., et al., The effect of topical application of Stryphnodendron adstringens (Martius) Coville extract and oral biotin supplementation on recovery of wounds from digital dermatitis surgery. Semina-Ciencias Agrarias, 2015. 36(3): p. 1955-1964.

319. Dadey, E., Bioequivalence of 2 Formulations of Sildenafil Oral Soluble Film 100 mg and Sildenafil Citrate (Viagra) 100 mg Oral Tablets in Healthy Male Volunteers. American Journal of Therapeutics, 2017. 24(4): p. E373-E380.

320. Dahmen, R., et al., Interaction of subcutaneous lixisenatide 20 mu g QD on pharmacokinetic and pharmacodynamic properties of oral ramipril 5 mg QD in steady state in young healthy males and females. 10th Congress of the European Association for Clinical Pharmacology and Therapeutics, ed. A. Vas. 2011. 85-92.

321. Dalessandri, D., et al., Treatment of recurrent aphthous stomatitis (RAS; aphthae; canker sores) with a barrier forming mouth rinse or topical gel formulation containing hyaluronic acid: a retrospective clinical study. BMC Oral Health, 2019. 19(1): p. 153.

322. Damkier, P. and K. Brosen, Quinidine as a probe for CYP3A4 activity: Intrasubject variability and lack of correlation with probe-based assays for CYP1A2, CYP2C9, CYP2C19, and CYP2D6. Clinical Pharmacology & Therapeutics, 2000. 68(2): p. 199-209.

323. Dannewitz, B., et al., Full-mouth disinfection as a nonsurgical treatment approach for drug-induced gingival overgrowth: a series of 11 cases. The International journal of periodontics & restorative dentistry, 2010. 30(1): p. 63-71.

324. Daood, U., et al., Effect of chitosan/riboflavin modification on resin/dentin interface: spectroscopic and microscopic investigations. J Biomed Mater Res A, 2013. 101(7): p. 1846-56.

325. Daood, U., et al., In vitro assessment of ribose modified two-step etch-and-rinse dentine adhesive. Dent Mater, 2018. 34(8): p. 1175-1187.

326. Darwish, M., S. Chang, and E.T. Hellriegel, A Pharmacokinetic Comparison of Single Doses of Once-Daily Cyclobenzaprine Extended-Release 15 mg and 30 mg: A Randomized, Double-Blind, Two-Period Crossover Study in Healthy Volunteers. Clinical Therapeutics, 2009. 31(1): p. 108-114.

327. Darwish, M. and E.T. Hellriegel, Steady-State Pharmacokinetics of Once-Daily Cyclobenzaprine Extended Release: A Randomized, Double-Blind, 2-Period Crossover Study in Healthy Volunteers. Clinical Therapeutics, 2011. 33(6): p. 746-753.

328. Darwish, M., E.T. Hellriegel, and F. Xie, Single-Dose Pharmacokinetics of Once-Daily Cyclobenzaprine Extended Release 30 mg versus Cyclobenzaprine Immediate Release 10 mg Three Times Daily in Healthy Young Adults A Randomized, Open-Label, Two-Period Crossover, Single-Centre Study. Clinical Drug Investigation, 2008. 28(12): p. 793-801.

329. Darwish, M. and F. Xie, Comparison of the Single-Dose Pharmacokinetics of Once-Daily Cyclobenzaprine Extended-Release 30 mg and Cyclobenzaprine Immediate-Release 10 mg Three Times Daily in the Elderly A Randomized, Open-Label, Crossover Study. Drugs & Aging, 2009. 26(2): p. 95-101.

330. Das, A., A. Mukherjee, and J. Chakrabarti, Sanguinarine: an evaluation of in vivo cytogenetic activity. Mutation research, 2004. 563(1): p. 81-7.

331. Davies, A.N., et al., Oral candidosis in community-based patients with advanced cancer. Journal of pain and symptom management, 2008. 35(5): p. 508-14.

332. Davis, A., R. Khorzad, and M. Whelan, Dynamic Upper Airway Obstruction Secondary to Severe Feline Asthma. Journal of the American Animal Hospital Association, 2013. 49(2): p. 142-147.

333. Davis, J.L., et al., Polymerase chain reaction of secA1 on sputum or oral wash samples for the diagnosis of pulmonary tuberculosis. Clinical infectious diseases : an official publication of the Infectious Diseases Society of America, 2009. 48(6): p. 725-32.

334. Davis, M.W. and S. Wason, Effect of Steady-State Atorvastatin on the Pharmacokinetics of a Single Dose of Colchicine in Healthy Adults Under Fasted Conditions. Clinical Drug Investigation, 2014. 34(4): p. 259-267.

335. Dawes, C. and G.H. Dibdin, A theoretical analysis of the effects of plaque thickness and initial salivary sucrose concentration on diffusion of sucrose into dental plaque and its conversion to acid during salivary clearance. J Dent Res, 1986. 65(2): p. 89-94.

336. de Ataide e Silva, T., et al., Can carbohydrate mouth rinse improve performance during exercise? A systematic review. Nutrients, 2013. 6(1): p. 1-10.

337. De Beule, F., P. Bercy, and A. Ferrant, The effectiveness of a preventive regimen on the periodontal health of patients undergoing chemotherapy for leukemia and lymphoma. J Clin Periodontol, 1991. 18(5): p. 346-7.

338. De Gregori, S., et al., Bioequivalence of a New Oral Levosulpiride Formulation Compared With a Standard One in Healthy Volunteers. Therapeutic Drug Monitoring, 2017. 39(2): p. 118-123.

339. de Oliveira, E.P. and R.C. Burini, Carbohydrate-dependent, exercise-induced gastrointestinal distress. Nutrients, 2014. 6(10): p. 4191-9.

340. de Oliveira, J.J., et al., Effect of Post-Activation Potentiation and Carbohydrate Mouth Rise on Repeated Sprint Ability in University Futsal Players. Journal of Exercise Physiology Online, 2020. 23(2): p. 29-40.

341. de Oliveira, J.J., et al., Effects of post-activation potentiation and carbohydrate mouth rinse on repeated sprint ability. Journal of Human Sport and Exercise, 2019. 14(1): p. 159-169.

342. De Oliveira, O.J., et al., Effects of post-activation potentiation and carbohydrate mouth rinse on repeated sprint ability. Journal of Human Sport & Exercise, 2019. 14(1): p. 159-169.

343. de Oliveira, T.A., et al., Effect of mouthrinses with different active agents in the prevention of initial dental erosion. Indian journal of dental research : official publication of Indian Society for Dental Research, 2015. 26(5): p. 508-13.

344. De Pauw, K., et al., Effects of caffeine and maltodextrin mouth rinsing on P300, brain imaging, and cognitive performance. Journal of Applied Physiology, 2014. 116(6): p. 776-782.

345. De Pauw, K., et al., Effects of caffeine and maltodextrin mouth rinsing on P300, brain imaging, and cognitive performance. Journal of applied physiology (Bethesda, Md. : 1985), 2015. 118(6): p. 776-82.

346. De Pauw, K., et al., Do Glucose and Caffeine Nasal Sprays Influence Exercise or Cognitive Performance? International Journal of Sports Physiology & Performance, 2017. 12(9): p. 1186-1191.

347. De Pauw, K., et al., Do Glucose and Caffeine Nasal Sprays Influence Exercise or Cognitive Performance? International journal of sports physiology and performance, 2017. 12(9): p. 1186-1191.

348. de Salles Painelli, V., H. Nicastro, and A.H. Lancha, Jr., Carbohydrate mouth rinse: does it improve endurance exercise performance? Nutr J, 2010. 9: p. 33.

349. de Siqueira, F.S.F., et al., Effect of Phosphoric Acid Containing MMP-Inactivator on the Properties of Resin Bonding to Eroded Dentin. Journal of Adhesive Dentistry, 2019. 21(2): p. 149-158.

350. De Smedt, B., et al., Intellectual abilities in a large sample of children with Velo-Cardio-Facial Syndrome: an update. Journal of intellectual disability research : JIDR, 2007. 51(Pt 9): p. 666-70.

351. De Smedt, B., et al., Basic number processing and difficulties in single-digit arithmetic: evidence from Velo-Cardio-Facial Syndrome. Cortex; a journal devoted to the study of the nervous system and behavior, 2009. 45(2): p. 177-88.

352. De Smedt, B., et al., Mathematical disabilities in children with velo-cardio-facial syndrome. Neuropsychologia, 2007. 45(5): p. 885-95.

353. de Souza, J.J., et al., EVALUATION OF THE ORAL HEALTH CONDITIONS OF VOLLEYBALL ATHLETES. Revista Brasileira De Medicina Do Esporte, 2020. 26(3): p. 239-242.

354. de Souza, J.J., et al., EVALUATION OF THE ORAL HEALTH CONDITIONS OF VOLLEYBALL ATHLETES. / EVALUACIÓN DE LAS CONDICIONES DE SALUD BUCAL DE LOS ATLETAS DE VÓLEIBOL. Revista Brasileira de Medicina do Esporte, 2020. 26(3): p. 239-242.

355. de Souza, M.M.A., et al., Detection of oral HPV infection - Comparison of two different specimen collection methods and two HPV detection methods. Diagnostic microbiology and infectious disease, 2018. 90(4): p. 267-271.

356. Dean, J.A., S.M. McDonald, and C.J. Palenik, Comparing infant oral glucose clearance with and without a water rinse. J Indiana Dent Assoc, 2003. 82(4): p. 25-9.

357. Dean, J.W., 3rd, B. Karshen, and P. Briggett, Lectins inhibit periodontal ligament fibroblast attachment, spreading and migration on laminin substrates. J Periodontal Res, 1999. 34(1): p. 41-9.

358. DeBernardi, M., F. DeBernardi, and P. Colombo, Randomised crossover comparison of the pharmacokinetic profiles of two sustained release morphine sulfate formulations in patients with cancer-related pain. Clinical Drug Investigation, 1997. 14: p. 28-33.

359. Decimoni, L.S., et al., Carbohydrate mouth rinsing improves resistance training session performance. International Journal of Sports Science & Coaching, 2018. 13(5): p. 804-809.

360. Decker, E.M., et al., Effect of xylitol/chlorhexidine versus xylitol or chlorhexidine as single rinses on initial biofilm formation of cariogenic streptococci. Quintessence Int, 2008. 39(1): p. 17-22.

361. Degnan, E.J. and A.N. Perlov, Infected oral lesions of cyclic neutropenia. Journal of oral medicine, 1973. 28(1): p. 29-31.

362. Deighton, K., et al., Mouth rinsing with a sweet solution increases energy expenditure and decreases appetite during 60 min of self-regulated walking exercise. Applied physiology, nutrition, and metabolism = Physiologie appliquee, nutrition et metabolisme, 2016. 41(12): p. 1255-1261.

363. Delecluse, C., R. Diels, and M. Goris, Effect of creatine supplementation on intermittent sprint running performance in highly trained athletes. Journal of Strength and Conditioning Research, 2003. 17(3): p. 446-454.

364. Delgado-Lopez, P.D., et al., Preservation of bone flap after craniotomy infection. Neurocirugia, 2009. 20(2): p. 124-131.

365. Delodder, F., et al., Incorporation and washout of n-3 PUFA after high dose intravenous and oral supplementation in healthy volunteers. Clinical Nutrition, 2015. 34(3): p. 400-408.

366. Demiguel, E.M., et al., COMPARATIVE-STUDY OF GALLOPAMIL VERSUS NIFEDIPINE IN PATIENTS WITH ISCHEMIC-HEART-DISEASE. International Journal of Cardiology, 1993. 40(2): p. 127-133.

367. Deng, S., et al., Effects of a Concomitant Single Oral Dose of Rifampicin on the Pharmacokinetics of Pravastatin in a Two-Phase, Randomized, Single-Blind, Placebo-Controlled, Crossover Study in Healthy Chinese Male Subjects. Clinical Therapeutics, 2009. 31(6): p. 1256-1263.

368. Densmore, L. and S.M.V. Pflueger, Using Interphase Fluorescence In Situ Hybridization (I-FISH) to Detect the Transfer of Infant Cells During Breastfeeding. Journal of Human Lactation, 2008. 24(4): p. 401-405.

369. Derosa, G., et al., Berberis aristata/Silybum marianum fixed combination on lipid profile and insulin secretion in dyslipidemic patients. Expert Opinion on Biological Therapy, 2013. 13(11): p. 1495-1506.

370. Derose, K.P., et al., The mediating role of perceived crime in gender and built environment associations with park use and park-based physical activity among park users in high poverty neighborhoods. Preventive medicine, 2019. 129: p. 105846.

371. Deroubaix, X., et al., ORAL BIOAVAILABILITY OF CHF1194, AN INCLUSION COMPLEX OF PIROXICAM AND BETA-CYCLODEXTRIN, IN HEALTHY-SUBJECTS UNDER SINGLE-DOSE AND STEADY-STATE CONDITIONS. European Journal of Clinical Pharmacology, 1995. 47(6): p. 531-536.

372. Descheemaeker, M.J., et al., Prader-Willi syndrome: new insights in the behavioural and psychiatric spectrum. Journal of intellectual disability research : JIDR, 2002. 46(Pt 1): p. 41-50.

373. Devenney, S., K. Collins, and M. Shortall, Effects of various concentrations of carbohydrate mouth rinse on cycling performance in a fed state. European journal of sport science, 2016. 16(8): p. 1073-8.

374. Devenney, S., et al., Effects of carbohydrate mouth rinse and caffeine on high-intensity interval running in a fed state. Applied physiology, nutrition, and metabolism = Physiologie appliquee, nutrition et metabolisme, 2018. 43(5): p. 517-521.

375. Dhariwal, R., et al., Oral submucous fibrosis: a report of two pediatric cases and a brief review. Journal of the Indian Society of Pedodontics and Preventive Dentistry, 2012. 30(1): p. 85-8.

376. Dharuman, J., M. Vasudhevan, and T. Ajithlal, High performance liquid chromatographic method for the determination of cetirizine and ambroxol in human plasma and urine-A boxcar approach. Journal of Chromatography B-Analytical Technologies in the Biomedical and Life Sciences, 2011. 879(25): p. 2624-2631.

377. Di Girolamo, G., et al., Parent drug and/or metabolite? Which of them is most appropriate to establish bioequivalence of two oral oxcarbazepine formulations in healthy volunteers? Expert Opinion on Pharmacotherapy, 2007. 8(10): p. 1415-1423.

378. Di Luigi, L., et al., The type 5 phosphodiesterase inhibitor tadalafil influences salivary cortisol, testosterone, and dehydroepiandrosterone sulphate responses to maximal exercise in healthy men. Journal of Clinical Endocrinology & Metabolism, 2008. 93(9): p. 3510-3514.

379. Dibbelt, L., et al., GROUP COMPARISON OF SERUM ETHINYL ESTRADIOL, SHBG AND CBG LEVELS IN 83 WOMEN USING 2 LOW-DOSE COMBINATION ORAL-CONTRACEPTIVES FOR 3 MONTHS. Contraception, 1991. 43(1): p. 1-21.

380. Diolosà, M., et al., Use of methacrylate-modified chitosan to increase the durability of dentine bonding systems. Biomacromolecules, 2014. 15(12): p. 4606-13.

381. Dodd, M.J., et al., Randomized clinical trial of chlorhexidine versus placebo for prevention of oral mucositis in patients receiving chemotherapy. Oncology nursing forum, 1996. 23(6): p. 921-7.

382. Dodd, M.J., et al., Factors influencing oral mucositis in patients receiving chemotherapy. Cancer practice, 2000. 8(6): p. 291-7.

383. Dodds, M.W. and W.M. Edgar, Effects of dietary sucrose levels on pH fall and acid-anion profile in human dental plaque after a starch mouth-rinse. Arch Oral Biol, 1986. 31(8): p. 509-12.

384. Dodds, M.W., S.C. Hsieh, and D.A. Johnson, The effect of increased mastication by daily gum-chewing on salivary gland output and dental plaque acidogenicity. J Dent Res, 1991. 70(12): p. 1474-8.

385. Doering, T.M., et al., The effect of a caffeinated mouth-rinse on endurance cycling time-trial performance. International journal of sport nutrition and exercise metabolism, 2014. 24(1): p. 90-7.

386. Dolan, P., et al., Effect of Carbohydrate, Caffeine, and Carbohydrate + Caffeine Mouth Rinsing on Intermittent Running Performance in Collegiate Male Lacrosse Athletes. Journal of strength and conditioning research, 2017. 31(9): p. 2473-2479.

387. Dolan, P., et al., EFFECT OF CARBOHYDRATE, CAFFEINE, AND CARBOHYDRATE plus CAFFEINE MOUTH RINSING ON INTERMITTENT RUNNING PERFORMANCE IN COLLEGIATE MALE LACROSSE ATHLETES. Journal of Strength and Conditioning Research, 2017. 31(9): p. 2473-2479.

388. Doll, J., et al., Contrast-enhanced ultrasound for determining muscular perfusion after oral intake of L-citrulline, L-arginine, and galloylated epicatechines A study protocol. Medicine, 2020. 99(41).

389. Dorling, J.L. and C.P. Earnest, Effect of carbohydrate mouth rinsing on multiple sprint performance. Journal of the International Society of Sports Nutrition, 2013. 10.

390. dos Santos, G.L., et al., Effects of elastic tape on kinematic parameters during a functional task in chronic hemiparetic subjects: A randomized sham-controlled crossover trial. Plos One, 2019. 14(1).

391. Doty, R.L., C. Wylie, and M. Potter, Validation of the Waterless Empirical Taste Test (WETT(®)). Behav Res Methods, 2021. 53(2): p. 864-873.

392. Douzi, W., et al., Cooling during exercise enhances performances, but the cooled body areas matter: A systematic review with meta-analyses. Scandinavian Journal of Medicine & Science in Sports, 2019. 29(11): p. 1660-1676.

393. Dowling, T.C., et al., Relative bioavailability of ketoprofen 20% in a poloxamer-lecithin organogel. American Journal of Health-System Pharmacy, 2004. 61(23): p. 2541-2544.

394. Dozono, H., et al., [Prevention of stomatitis induced by anti-cancer drugs]. Gan to kagaku ryoho. Cancer & chemotherapy, 1989. 16(10): p. 3449-51.

395. Drengenes, C., et al., Laboratory contamination in airway microbiome studies. BMC microbiology, 2019. 19(1): p. 187.

396. Driggers, R.W. and A. Baschat, The 12th meeting of the Diabetes in Pregnancy Study Group of North America (DPSG-NA): introduction and overview. Journal of Maternal-Fetal & Neonatal Medicine, 2012. 25(1): p. 3-4.

397. Ducharme, M.P., L.H. Warbasse, and D.J. Edwards, DISPOSITION OF INTRAVENOUS AND ORAL CYCLOSPORINE AFTER ADMINISTRATION WITH GRAPEFRUIT JUICE. Clinical Pharmacology & Therapeutics, 1995. 57(5): p. 485-491.

398. Duckworth, R.M. and S. Jones, On the relationship between the rate of salivary flow and salivary fluoride clearance. Caries Res, 2015. 49(2): p. 141-6.

399. Dugas, J.P., et al., Rates of fluid ingestion alter pacing but not thermoregulatory responses during prolonged exercise in hot and humid conditions with appropriate convective cooling. European Journal of Applied Physiology, 2009. 105(1): p. 69-80.

400. Dunkin, J.E. and S.M. Phillips, The Effect of a Carbohydrate Mouth Rinse on Upper-Body Muscular Strength and Endurance. Journal of strength and conditioning research, 2017. 31(7): p. 1948-1953.

401. Dunn, J.T., et al., The sites of thyroid hormone formation in rabbit thyroglobulin. The Journal of biological chemistry, 1987. 262(35): p. 16948-52.

402. Durkin, M., H. Akeroyd, and A. Holliday, Carbohydrate mouth rinse improves resistance exercise capacity in the glycogen-lowered state. Applied Physiology Nutrition and Metabolism, 2021. 46(2): p. 126-132.

403. Dušková, M., et al., The role of taste in cephalic phase of insulin secretion. Prague Med Rep, 2013. 114(4): p. 222-30.

404. Duus, K.M., et al., Wild-type Kaposi's sarcoma-associated herpesvirus isolated from the oropharynx of immune-competent individuals has tropism for cultured oral epithelial cells. Journal of Virology, 2004. 78(8): p. 4074-4084.

405. Edelman, A.B., et al., Impact of the prostaglandin synthase-2 inhibitor celecoxib on ovulation and luteal events in women. Contraception, 2013. 87(3): p. 352-357.

406. Edelman, A.B., et al., Combined oral contraceptive interference with the ability of ulipristal acetate to delay ovulation: A prospective cohort study. Contraception, 2018. 98(6): p. 463-466.

407. Edmonds, C.J., N. Harte, and M. Gardner, How does drinking water affect attention and memory? The effect of mouth rinsing and mouth drying on children's performance. Physiology & behavior, 2018. 194: p. 233-238.

408. Edmonds, C.J., et al., At what stage in the drinking process does drinking water affect attention and memory? Effects of mouth rinsing and mouth drying in adults. Psychological Research-Psychologische Forschung, 2021. 85(1): p. 214-222.

409. Edwards, A.M., et al., Influence of moderate dehydration on soccer performance: physiological responses to 45 min of outdoor match-play and the immediate subsequent performance of sport-specific and mental concentration tests. British journal of sports medicine, 2007. 41(6): p. 385-91.

410. Edwards, A.M., et al., Influence of moderate dehydration on soccer performance: physiological responses to 45 mm of outdoor match-play and the immediate subsequent performance of sport-specific and mental concentration tests. British Journal of Sports Medicine, 2007. 41(6): p. 385-391.

411. Egginger, G., et al., Stereoselective high-performance liquid chromatographic assay of (+/-)-delmopinol in plasma using solid-phase extraction, a chiral derivatizing agent and electrochemical detection. Journal of chromatography. A, 1994. 666(1-2): p. 275-82.

412. Egginger, G., et al., STEREOSELECTIVE HIGH-PERFORMANCE LIQUID-CHROMATOGRAPHIC ASSAY OF (+/-)-DELMOPINOL IN PLASMA USING SOLID-PHASE EXTRACTION, A CHIRAL DERIVATIZING AGENT AND ELECTROCHEMICAL DETECTION. Journal of Chromatography A, 1994. 666(1-2): p. 275-282.

413. Ehlert, A.M., H.M. Twiddy, and P.B. Wilson, The Effects of Caffeine Mouth Rinsing on Exercise Performance: A Systematic Review. International Journal of Sport Nutrition & Exercise Metabolism, 2020. 30(5): p. 362-373.

414. Eibye, K., et al., Effect of one-week oral or inhaled salbutamol treatment with washout on repeated sprint performance in trained subjects. Translational Sports Medicine, 2021. 4(2): p. 241-249.

415. Eisen, D., et al., Effect of topical cyclosporine rinse on oral lichen planus. A double-blind analysis. The New England journal of medicine, 1990. 323(5): p. 290-4.

416. Eke, P.I., et al., Self-reported measures for surveillance of periodontitis. Journal of dental research, 2013. 92(11): p. 1041-7.

417. Ekstrand, J., et al., Effect of repeated intake of a sugar free fluoride-containing chewing gum on acidogenicity and microbial composition of dental plaque. Scand J Dent Res, 1985. 93(4): p. 309-14.

418. El-Sayed, S., et al., A pilot study evaluating the safety and microbiologic efficacy of an economically viable antimicrobial lozenge in patients with head and neck cancer receiving radiation therapy. Head Neck, 2002. 24(1): p. 6-15.

419. ElEmbaby, A.E., The Effects of Mouth Rinses on the Color Stability of Resin-Based Restorative Materials. Journal of Esthetic and Restorative Dentistry, 2014. 26(4): p. 264-271.

420. ElEmbaby, A.E.-S., The effects of mouth rinses on the color stability of resin-based restorative materials. Journal of esthetic and restorative dentistry : official publication of the American Academy of Esthetic Dentistry ... [et al.], 2014. 26(4): p. 264-71.

421. Eliades, G., et al., Interactions of dentine desensitisers with human dentine: morphology and composition. Journal of dentistry, 2013. 41 Suppl 4: p. S28-39.

422. Elledge, E.S., et al., The effects of topical oral clindamycin antibiotic rinses on the bacterial content of saliva on healthy human subjects. Otolaryngol Head Neck Surg, 1991. 105(6): p. 836-9.

423. Elmogy, M., et al., Oral ivermectin in the treatment of scabies. International Journal of Dermatology, 1999. 38(12): p. 926-928.

424. Elmore, A.R., F.A. Andersen, and P. Cosmetic Ingredient Rev Expert, Final report on the safety assessment of Aluminum Silicate, Calcium Silicate, Magnesium Aluminum Silicate, Magnesium Silicate, Magnesium Trisilicate, Sodium Magnesium Silicate, Zirconium Silicate, Attapulgite, Bentonite, Fuller's Earth, Hectorite, Kaolin, Lithium Magnesium Silicate, Lithium Magnesium Sodium Silicate, Montmorillonite, Pyrophyllite, and Zeolite. International Journal of Toxicology, 2003. 22: p. 37-102.

425. Elsayed, Y.M., et al., EFFECTS OF ORAL-ADMINISTRATION OF COLESTIPOL AND CHOLESTYRAMINE ON THE PHARMACOKINETICS OF KETOPROFEN ADMINISTERED INTRAMUSCULARLY IN MAN. International Journal of Pharmaceutics, 1994. 109(2): p. 107-113.

426. Ely, B.R., et al., Limitations of salivary osmolality as a marker of hydration status. Medicine and science in sports and exercise, 2011. 43(6): p. 1080-4.

427. Emelyanov, D.V., RESULTS OF QUESTIONING PATIENTS WITH NON-ALCOHOLIC FATTY LIVER DISEASE IN THE CONDITIONS OF DENTAL PRACTICE. Wiad Lek, 2021. 74(3 cz 1): p. 504-507.

428. Engebretson, S., et al., Design features of the Diabetes and Periodontal Therapy Trial (DPTT): a multicenter randomized single-masked clinical trial testing the effect of nonsurgical periodontal therapy on glycosylated hemoglobin (HbA1c) levels in subjects with type 2 diabetes and chronic periodontitis. Contemp Clin Trials, 2013. 36(2): p. 515-26.

429. Epstein, J.B., et al., Topical azathioprine in the treatment of immune-mediated chronic oral inflammatory conditions - A series of cases. Oral Surgery Oral Medicine Oral Pathology Oral Radiology and Endodontology, 2001. 91(1): p. 56-61.

430. Epstein, J.B. and D.E. Reece, Topical cyclosporin A for treatment of oral chronic graft-versus-host disease. Bone marrow transplantation, 1994. 13(1): p. 81-6.

431. Epstein, J.B., D.C. Villines, and S. Baker, Efficacy of a glycopolymer-based oral rinse upon pain associated with ulcerative and erosive lesions of the oral mucosa: A within-subject pilot study. Oral Surg Oral Med Oral Pathol Oral Radiol, 2018. 126(3): p. 240-245.

432. Epstein, J.B., et al., Management of dry mouth: assessment of oral symptoms after use of a polysaccharide-based oral rinse. Oral Surg Oral Med Oral Pathol Oral Radiol, 2017. 123(1): p. 76-83.

433. Epstein, J.B. and F.L. Wong, The efficacy of sucralfate suspension in the prevention of oral mucositis due to radiation therapy. Int J Radiat Oncol Biol Phys, 1994. 28(3): p. 693-8.

434. Equi, A., et al., Long term azithromycin in children with cystic fibrosis: a randomised, placebo-controlled crossover trial. Lancet, 2002. 360(9338): p. 978-984.

435. Erhardt, M.C., et al., Adjunctive use of an anti-oxidant agent to improve resistance of hybrid layers to degradation. J Dent, 2011. 39(1): p. 80-7.

436. Erickson, L., Oral health promotion and prevention for older adults. Dent Clin North Am, 1997. 41(4): p. 727-50.

437. Eriksen, H.M., et al., Characterization of saliva proteins from 'stainers' and 'non-stainers' adsorbed to hydroxyapatite. Acta Odontol Scand, 1985. 43(2): p. 115-20.

438. Eriksson, B., et al., Metabolic fate of delmopinol in man after mouth rinsing and after oral administration. Xenobiotica, 2000. 30(2): p. 179-92.

439. Erjavec, M.K., et al., Morphine-fluoxetine interactions in healthy volunteers: Analgesia and side effects. Journal of Clinical Pharmacology, 2000. 40(11): p. 1286-1295.

440. Erovic Ademovski, S., P. Lingström, and S. Renvert, The effect of different mouth rinse products on intra-oral halitosis. Int J Dent Hyg, 2016. 14(2): p. 117-23.

441. Evenson, K.R., et al., United States' neighborhood park use and physical activity over two years: The National Study of Neighborhood Parks. Preventive medicine, 2019. 123: p. 117-122.

442. Fairchild, T.J., et al., GLUCOSE INGESTION DOES NOT IMPROVE MAXIMAL ISOKINETIC FORCE. Journal of Strength and Conditioning Research, 2016. 30(1): p. 194-199.

443. Fakhry-Smith, S., et al., Clearance of sodium lauryl sulphate from the oral cavity. Journal of clinical periodontology, 1997. 24(5): p. 313-7.

444. FakhrySmith, S., et al., Clearance of sodium lauryl sulphate from the oral cavity. Journal of Clinical Periodontology, 1997. 24(5): p. 313-317.

445. Fallah, Z., et al., Effect of fermented camel milk on glucose metabolism, insulin resistence, and inflammatory biomarkers of adolescents with metabolic syndrome: A double-blind, randomized, crossover trail. Journal of Research in Medical Sciences, 2018. 23.

446. Faller, R.V. and S.L. Eversole, Enamel protection from acid challenge--benefits of marketed fluoride dentifrices. J Clin Dent, 2013. 24(1): p. 25-30.

447. Fanchin, R., et al., Vaginal versus oral E-2 administration: effects on endometrial thickness, uterine perfusion and contractility. Fertility and Sterility, 2001. 76(5): p. 994-998.

448. Faraday, N., P.J. GoldschmidtClermont, and P.F. Bray, Gender differences in platelet GPIIb-IIIa activation. Thrombosis and Haemostasis, 1997. 77(4): p. 748-754.

449. Fares, E.J. and B. Kayser, Carbohydrate mouth rinse effects on exercise capacity in pre- and postprandial States. J Nutr Metab, 2011. 2011: p. 385962.

450. Fatemeh, M., et al., Effect of water rinsing after acidulated phosphate fluoride gel on dental plaque acidity: an in situ study. Pediatr Dent, 2014. 36(1): p. 56-60.

451. Fattore, C., et al., Induction of ethinylestradiol and levonorgestrel metabolism by oxcarbazepine in healthy women. Epilepsia, 1999. 40(6): p. 783-787.

452. Faverani, L.P., et al., Corrosion kinetics and topography analysis of Ti-6Al-4V alloy subjected to different mouthwash solutions. Materials science & engineering. C, Materials for biological applications, 2014. 43: p. 1-10.

453. Faverani, L.P., et al., Corrosion kinetics and topography analysis of Ti-6Al-4V alloy subjected to different mouthwash solutions. Materials Science & Engineering C-Materials for Biological Applications, 2014. 43: p. 1-10.

454. Favia, G., et al., Medication-related osteonecrosis of the jaw: Surgical or non-surgical treatment? Oral diseases, 2018. 24(1-2): p. 238-242.

455. Favretto, C.O., et al., In vitro evaluation of the effect of mouth rinse with trimetaphosphate on enamel demineralization. Caries research, 2013. 47(5): p. 532-8.

456. Fawzy, A.S., et al., Chitosan/Riboflavin-modified demineralized dentin as a potential substrate for bonding. J Mech Behav Biomed Mater, 2013. 17: p. 278-89.

457. Fedorowicz, Z., et al., Mouthrinses for the treatment of halitosis. The Cochrane database of systematic reviews, 2008(4): p. CD006701.

458. Fejerskov, O., A.A. Scheie, and F. Manji, The effect of sucrose on plaque pH in the primary and permanent dentition of caries-inactive and -active Kenyan children. J Dent Res, 1992. 71(1): p. 25-31.

459. Ferguson, K.K., et al., Personal care product use among adults in NHANES: associations between urinary phthalate metabolites and phenols and use of mouthwash and sunscreen. Journal of exposure science & environmental epidemiology, 2017. 27(3): p. 326-332.

460. Fernandes, M.J., et al., Assessing oral health-related quality of life in general dental practice in Scotland: validation of the OHIP-14. Community dentistry and oral epidemiology, 2006. 34(1): p. 53-62.

461. Fernandes-Naglik, L., et al., The clinical and microbiological effects of a novel acidified sodium chlorite mouthrinse on oral bacterial mucosal infections. Oral Dis, 2001. 7(5): p. 276-80.

462. Ferreira, A.M.J., et al., Carbohydrate Mouth Rinse and Hydration Strategies on Cycling Performance in 30 Km Time Trial: A Randomized, Crossover, Controlled Trial. Journal of Sports Science and Medicine, 2018. 17(2): p. 181-187.

463. Ferreira, A.M.J., et al., The effect of carbohydrate mouth rinse on performance, biochemical and psychophysiological variables during a cycling time trial: a crossover randomized trial. Journal of the International Society of Sports Nutrition, 2018. 15.

464. Ferreira, A.M.J., et al., Carbohydrate Mouth Rinse and Hydration Strategies on Cycling Performance in 30 Km Time Trial: A Randomized, Crossover, Controlled Trial. J Sports Sci Med, 2018. 17(2): p. 181-187.

465. Ferreira, A.M.J., et al., The effect of carbohydrate mouth rinse on performance, biochemical and psychophysiological variables during a cycling time trial: a crossover randomized trial. J Int Soc Sports Nutr, 2018. 15: p. 23.

466. Ferreira, A.M.J., et al., Carbohydrate Mouth Rinse and Hydration Strategies on Cycling Performance in 30 Km Time Trial: A Randomized, Crossover, Controlled Trial. Journal of sports science & medicine, 2018. 17(2): p. 181-187.

467. Ferreira, A.M.J., et al., The effect of carbohydrate mouth rinse on performance, biochemical and psychophysiological variables during a cycling time trial: a crossover randomized trial. Journal of the International Society of Sports Nutrition, 2018. 15: p. 23.

468. Ferreira, R.C., et al., Assistive technologies for improving the oral hygiene of leprosy patients residing in a former leprosy colony in Betim, Minas Gerais, Brazil. Plos One, 2018. 13(7).

469. Ferreira, R.C., et al., Assistive technologies for improving the oral hygiene of leprosy patients residing in a former leprosy colony in Betim, Minas Gerais, Brazil. PloS one, 2018. 13(7): p. e0200503.

470. Ferron, G.M., et al., Oral bioavailability of pantoprazole suspended in sodium bicarbonate solution. American Journal of Health-System Pharmacy, 2003. 60(13): p. 1324-1329.

471. Feugang, J.M., et al., Beneficial effects of relaxin on motility characteristics of stored boar spermatozoa. Reproductive biology and endocrinology : RB&E, 2015. 13: p. 24.

472. Feugang, J.M., et al., Self-illuminating quantum dots for non-invasive bioluminescence imaging of mammalian gametes. Journal of nanobiotechnology, 2015. 13: p. 38.

473. Fidler, P., et al., Prospective evaluation of a chamomile mouthwash for prevention of 5-FU-induced oral mucositis. Cancer, 1996. 77(3): p. 522-5.

474. Fidler, P., et al., Prospective evaluation of a chamomile mouthwash for prevention of 5-FU-induced oral mucositis. Cancer, 1996. 77(3): p. 522-525.

475. Figueiredo, N., et al., Acute caffeine mouth rinsing does not improve 10-km running performance in CYP1A2 C-allele carriers. Clinical Nutrition Espen, 2021. 42: p. 93-97.

476. Figueiredo, N., et al., Acute caffeine mouth rinsing does not improve 10-km running performance in CYP1A2 C-allele carriers. Clin Nutr ESPEN, 2021. 42: p. 93-97.

477. Firestone, A.R. and H.R. Muhlemann, In vivo pH of plaque-covered and plaque-free interdental surfaces in humans following a sucrose rinse. Clin Prev Dent, 1985. 7(4): p. 24-6.

478. Fisher, B.M., et al., Carriage of Candida species in the oral cavity in diabetic patients: relationship to glycaemic control. J Oral Pathol, 1987. 16(5): p. 282-4.

479. Ford-Johnson, L., et al., Cognitive Effects of Modafinil in Patients With Multiple Sclerosis: A Clinical Trial. Rehabilitation Psychology, 2016. 61(1): p. 82-91.

480. Formaker, B.K., et al., Responses of the rat chorda tympani nerve to glutamate-sucrose mixtures. Chem Senses, 2004. 29(6): p. 473-82.

481. Forss, H., L. Seppä, and P. Alakuijala, Plaque accumulation on glass ionomer filling materials. Proc Finn Dent Soc, 1991. 87(3): p. 343-50.

482. Fosdick, L.S., et al., A comparison of pH values of in vivo dental plaque after sucrose and sorbitol mouth rinses. J Am Dent Assoc, 1957. 55(2): p. 191-5.

483. Fouadtarazi, F.M., M. Okabe, and H. Goren, ALPHA-SYMPATHOMIMETIC TREATMENT OF AUTONOMIC INSUFFICIENCY WITH ORTHOSTATIC HYPOTENSION. American Journal of Medicine, 1995. 99(6): p. 604-610.

484. Fraga, C., et al., Carbohydrate mouth rinse enhances time to exhaustion during treadmill exercise. Clinical Physiology and Functional Imaging, 2017. 37(1): p. 17-22.

485. Fraga, C., et al., Carbohydrate mouth rinse enhances time to exhaustion during treadmill exercise. Clin Physiol Funct Imaging, 2017. 37(1): p. 17-22.

486. Fraga, C., et al., Carbohydrate mouth rinse enhances time to exhaustion during treadmill exercise. Clinical physiology and functional imaging, 2017. 37(1): p. 17-22.

487. Franca, C.M., et al., Severe oral manifestations of chronic graft-vs.-host disease. Journal of the American Dental Association (1939), 2001. 132(8): p. 1124-7.

488. Francisconi-dos-Rios, L.F., et al., Role of chlorhexidine in bond strength to artificially eroded dentin over time. The journal of adhesive dentistry, 2015. 17(2): p. 133-9.

489. Franklin, R.D. and W.H. Kutteh, Characterization of immunoglobulins and cytokines in human cervical mucus: influence of exogenous and endogenous hormones. Journal of Reproductive Immunology, 1999. 42(2): p. 93-106.

490. Frasseto, F., et al., Relationship among salivary carbonic anhydrase VI activity and flow rate, biofilm pH and caries in primary dentition. Caries Res, 2012. 46(3): p. 194-200.

491. Freitas-Fernandes, L.B., et al., Characterization of the binding of delmopinol to salivary precipitates. Brazilian dental journal, 2001. 12(3): p. 173-7.

492. Friedrich, C., et al., Effect of Multiple Oral Doses of Linagliptin on the Steady-State Pharmacokinetics of a Combination Oral Contraceptive in Healthy Female Adults An Open-Label, Two-Period, Fixed-Sequence, Multiple-Dose Study. Clinical Drug Investigation, 2011. 31(9): p. 643-653.

493. Frostell, G., Effects of mouth rinses on the pH of dental plaques. Sven Tandlak Tidskr, 1971. 64(4): p. 227-32.

494. Frostell, G., Effects of mouth rinses with sucrose, glucose, fructose, lactose, sorbitol and Lycasin on the pH of dental plaque. Odontol Revy, 1973. 24(3): p. 217-26.

495. Fujinami, A., T. Miyazawa, and Y. Kobayashi, Development of a method for the quantitation of benzphetamine metabolites in human urine by high-performance liquid chromatography. Annals of Clinical Biochemistry, 1998. 35: p. 775-779.

496. Fujita, T., et al., Effect of L-phenylalanine supplementation and a high-protein diet on pharmacokinetics of cefdinir in healthy volunteers: an exploratory study. Journal of Clinical Pharmacy and Therapeutics, 2007. 32(3): p. 277-285.

497. Fukazawa, M., et al., [High incidence-rate of oral mucositis in breast cancer patients receiving anthracycline-based chemotherapy (FEC100)]. Gan to kagaku ryoho. Cancer & chemotherapy, 2012. 39(3): p. 395-8.

498. Funck, V.R., et al., Differential effects of atorvastatin treatment and withdrawal on pentylenetetrazol-induced seizures. Epilepsia, 2011. 52(11): p. 2094-2104.

499. Furlani, T.A., et al., Effect of calcium pre-rinse and fluoride dentifrice on enamel and on dental plaque formed in situ. Oral Health Prev Dent, 2009. 7(1): p. 23-8.

500. Furuse, A.Y., et al., Bond strength of fiber-reinforced posts to deproteinized root canal dentin. J Contemp Dent Pract, 2014. 15(5): p. 581-6.

501. Furuyashiki, T., et al., Effect of oral supplementation with enzymatically synthesized glycogen (ESG) on cognitive function: a randomized, double-blind, placebo-controlled study. Functional Foods in Health and Disease, 2020. 10(4): p. 155-167.

502. Gabre, P., D. Birkhed, and L. Gahnberg, Fluoride retention of a mucosa adhesive paste compared with other home-care fluoride products. Caries Res, 2008. 42(4): p. 240-6.

503. Gachet, C., et al., THE THIENOPYRIDINE TICLOPIDINE SELECTIVELY PREVENTS THE INHIBITORY EFFECTS OF ADP BUT NOT OF ADRENALINE ON CAMP LEVELS RAISED BY STIMULATION OF THE ADENYLATE-CYCLASE OF HUMAN PLATELETS BY PGE1. Biochemical Pharmacology, 1990. 40(12): p. 2683-2687.

504. Gagliardi, L., et al., Continuous Subcutaneous Hydrocortisone Infusion Therapy in Addison's Disease: A Randomized, Placebo-Controlled Clinical Trial. Journal of Clinical Endocrinology & Metabolism, 2014. 99(11): p. 4149-4157.

505. Gai, M.N., E. Costa, and A. Arancibia, Bioavailability of a controlled-release cyclobenzaprine tablet and influence of a high fat meal on bioavailability. International Journal of Clinical Pharmacology and Therapeutics, 2009. 47(4): p. 269-274.

506. Galan-Herrera, J.F., et al., Bioavailability of Two Oral Formulations of a Single Dose of Levofloxacin 500 mg: An Open-Label, Randomized, Two-Period Crossover Comparison in Healthy Mexican Volunteers. Clinical Therapeutics, 2009. 31(8): p. 1796-1803.

507. Gam, S., K. Guelfi, and P. Fournier, New Insights into Enhancing Maximal Exercise Performance Through the Use of a Bitter Tastant. Sports Medicine, 2016. 46(10): p. 1385-1390.

508. Gam, S., et al., Mouth rinsing and ingestion of a bitter-tasting solution increases corticomotor excitability in male competitive cyclists. European Journal of Applied Physiology, 2015. 115(10): p. 2199-2204.

509. Gam, S., K.J. Guelfi, and P.A. Fournier, Opposition of carbohydrate in a mouth-rinse solution to the detrimental effect of mouth rinsing during cycling time trials. International journal of sport nutrition and exercise metabolism, 2013. 23(1): p. 48-56.

510. Gam, S., K.J. Guelfi, and P.A. Fournier, Mouth rinsing and ingesting a bitter solution improves sprint cycling performance. Medicine and science in sports and exercise, 2014. 46(8): p. 1648-57.

511. Gam, S., K.J. Guelfi, and P.A. Fournier, New Insights into Enhancing Maximal Exercise Performance Through the Use of a Bitter Tastant. Sports medicine (Auckland, N.Z.), 2016. 46(10): p. 1385-90.

512. Gam, S., et al., Mouth rinsing and ingestion of a bitter-tasting solution increases corticomotor excitability in male competitive cyclists. European journal of applied physiology, 2015. 115(10): p. 2199-204.

513. Gam, S., et al., Mouth rinsing with a bitter solution without ingestion does not improve sprint cycling performance. European Journal of Applied Physiology, 2015. 115(1): p. 129-138.

514. Gam, S., et al., Mouth rinsing with a bitter solution without ingestion does not improve sprint cycling performance. European journal of applied physiology, 2015. 115(1): p. 129-38.

515. Ganss, C., et al., Effectiveness of two fluoridation measures on erosion progression in human enamel and dentine in vitro. Caries research, 2001. 35(5): p. 325-30.

516. Ganss, C., et al., Conventional and anti-erosion fluoride toothpastes: effect on enamel erosion and erosion-abrasion. Caries Res, 2011. 45(6): p. 581-9.

517. Gant, N., C.M. Stinear, and W.D. Byblow, Carbohydrate in the mouth immediately facilitates motor output. Brain research, 2010. 1350: p. 151-8.

518. Gao, Y., et al., [Effect of dentin proteoglycans on the bonding properties of dentin]. Zhonghua Kou Qiang Yi Xue Za Zhi, 2014. 49(12): p. 753-7.

519. Garcia-Gea, C., et al., Rupatadine does not potentiate the CNS depressant effects of lorazepam: randomized, double-blind, crossover, repeated dose, placebo-controlled study. British Journal of Clinical Pharmacology, 2010. 69(6): p. 663-674.

520. García-Godoy, F., et al., Fluoride dentifrice containing xylitol: in vitro root caries formation. Am J Dent, 2013. 26(1): p. 56-60.

521. Garcia-Godoy, F., et al., Comparative bioavailability and antimicrobial activity of cetylpyridinium chloride mouthrinses in vitro and in vivo. American journal of dentistry, 2014. 27(4): p. 185-90.

522. Garcia-Godoy, F., et al., Comparative bioavailability and antimicrobial activity of cetylpyridinium chloride mouthrinses in vitro and in vivo. American Journal of Dentistry, 2014. 27(4): p. 185-190.

523. Garrido Urrutia, C., et al., Oral health practices and beliefs among caregivers of the dependent elderly. Gerodontology, 2012. 29(2): p. e742-7.

524. Gau, C.-H., et al., Can chlorhexidine mouthwash twice daily ameliorate cyclosporine-induced gingival overgrowth? Journal of the Formosan Medical Association = Taiwan yi zhi, 2013. 112(3): p. 131-7.

525. Gau, V. and D. Wong, Oral fluid nanosensor test (OFNASET) with advanced electrochemical-based molecular analysis platform, in Oral-Based Diagnostics, D. Malamud and R.S. Niedbala, Editors. 2007. p. 401-410.

526. Geary, N., Is the control of fat ingestion sexually differentiated? Physiology & Behavior, 2004. 83(4): p. 659-671.

527. Georgios, A., T. Vassiliki, and K. Sotirios, Acidogenicity and acidurance of dental plaque and saliva sediment from adults in relation to caries activity and chlorhexidine exposure. J Oral Microbiol, 2015. 7: p. 26197.

528. Gerardu, V.A., et al., Effects of various rinsing protocols after the use of amine fluoride/stannous fluoride toothpaste on the acid production of dental plaque and tongue flora. Caries Res, 2006. 40(3): p. 245-50.

529. Gerardu, V.A.M., et al., Effects of various rinsing protocols after the use of amine fluoride/stannous fluoride toothpaste on the acid production of dental plaque and tongue flora. Caries research, 2006. 40(3): p. 245-50.

530. Germaine, M., K. Collins, and M. Shortall, The Effect of Caffeine Ingestion and Carbohydrate Mouth Rinse on High-Intensity Running Performance. Sports, 2019. 7(3).

531. Ghai, S., et al., In spite of successful curative surgery for buccal mucosa carcinoma the health-related quality-of-life continues to remain poor. Oral and Maxillofacial Surgery-Heidelberg.

532. Giachetti, L., F. Bertini, and D. Scaminaci Russo, Investigation into the nature of dentin resin tags: a scanning electron microscopic morphological analysis of demineralized bonded dentin. J Prosthet Dent, 2004. 92(3): p. 233-8.

533. Giamberardino, M.A., et al., Effects of prolonged L-carnitine administration on delayed muscle pain and CK release after eccentric effort. International Journal of Sports Medicine, 1996. 17(5): p. 320-324.

534. Giertsen, E., H. Emberland, and A.A. Scheie, Effects of mouth rinses with xylitol and fluoride on dental plaque and saliva. Caries Res, 1999. 33(1): p. 23-31.

535. Giertsen, E. and A.A. Scheie, Effects of chlorhexidine-fluoride mouthrinses on viability, acidogenic potential, and glycolytic profile of established dental plaque. Caries research, 1995. 29(3): p. 181-7.

536. Gilfillan, A., R. Axton, and D.J. Brock, Mass screening for cystic fibrosis heterozygotes: two assay systems compared. Clinical chemistry, 1994. 40(2): p. 197-9.

537. Gilfillan, A., R. Axton, and D.J.H. Brock, MASS-SCREENING FOR CYSTIC-FIBROSIS HETEROZYGOTES - 2 ASSAY SYSTEMS COMPARED. Clinical Chemistry, 1994. 40(2): p. 197-199.

538. Gill, S.K., M. Price, and R.J.S. Costa, Measurement of saliva flow rate in healthy young humans: influence of collection time and mouthrinse water temperature. European journal of oral sciences, 2016. 124(5): p. 447-453.

539. Giovanoulis, G., et al., Evaluation of exposure to phthalate esters and DINCH in urine and nails from a Norwegian study population. Environmental Research, 2016. 151: p. 80-90.

540. Gisleskog, P.O.O., et al., Nicotine Population Pharmacokinetics in Healthy Smokers After Intravenous, Oral, Buccal and Transdermal Administration. Clinical Pharmacokinetics, 2021. 60(4): p. 541-561.

541. Giuliano, F., et al., Switching from intracavernous prostaglandin E1 injections to oral sildenafil citrate in patients with erectile dysfunction: Results of a multicenter European study. Journal of Urology, 2000. 164(3): p. 708-711.

542. Gizaw, Z., et al., Sanitation predictors of childhood morbidities in Ethiopia: evidence from Dabat Health and Demographic Surveillance System. Environmental Health and Preventive Medicine, 2019. 24(1).

543. Goedsche, K., et al., Serielle kaltwasserreize (kneipp'scher oberguss) bei patienten mit chronisch obstruktiver bronchitis (COPD). Forschende Komplementarmedizin, 2007. 14(3): p. 158-166.

544. Goldberg, D.J., et al., Impact of Oral Sildenafil on Exercise Performance in Children and Young Adults After the Fontan Operation A Randomized, Double-Blind, Placebo-Controlled, Crossover Trial. Circulation, 2011. 123(11): p. 1185-1193.

545. Goldfarb, D.M., et al., Self-Collected Saline Gargle Samples as an Alternative to Health Care Worker-Collected Nasopharyngeal Swabs for COVID-19 Diagnosis in Outpatients. Journal of Clinical Microbiology, 2021. 59(4).

546. Goldwater, D.R., et al., Effect of ketoconazole on the pharmacokinetics of maribavir in healthy adults. Antimicrobial Agents and Chemotherapy, 2008. 52(5): p. 1794-1798.

547. Gonçalves, N.C., et al., Effect of xylitol:sorbitol on fluoride enamel demineralization reduction in situ. J Dent, 2006. 34(9): p. 662-7.

548. Gonçalves, N.C., et al., [Effect of sodium fluoride mouth rinses containing xylitol and sorbitol on the number of Streptococcus mutans from human saliva]. Rev Panam Salud Publica, 2001. 9(1): p. 30-4.

549. Gonzalez, A.M., et al., Acute Caffeine Mouth Rinse Does Not Change the Hydration Status following a 10 km Run in Recreationally Trained Runners. BioMed research international, 2020. 2020: p. 6598753.

550. Gonzalez, A.M., et al., Acute Caffeine Mouth Rinse Does Not Change the Hydration Status following a 10 km Run in Recreationally Trained Runners. Biomed Res Int, 2020. 2020: p. 6598753.

551. Goodman, S.P.J. and F.E. Marino, Thirst perception exacerbates objective mental fatigue. Neuropsychologia, 2021. 150.

552. Goodrich, J.A., et al., Intermittent low dose carbon monoxide inhalation does not influence glucose regulation in overweight adults: a randomized controlled crossover trial. Experimental Physiology, 2020. 105(3): p. 460-467.

553. Gooptu, C. and R.C. Staughton, Use of topical cyclosporin in oral pemphigus. Journal of the American Academy of Dermatology, 1998. 38(5 Pt 2): p. 860-1.

554. Gordi, T., et al., Pharmacokinetics of gabapentin after a single day and at steady state following the administration of gastric-retentive-extended-release and immediate-release tablets: A randomized, open-label, multiple-dose, three-way crossover, exploratory study in healthy subjects. Clinical Therapeutics, 2008. 30(5): p. 909-916.

555. Gorodischer, R. and G. Koren, Salivary excretion of drugs in children: theoretical and practical issues in therapeutic drug monitoring. Dev Pharmacol Ther, 1992. 19(4): p. 161-77.

556. Goterris, L., et al., Molecular Diagnosis of Pneumocystis jirovecii Pneumonia by Use of Oral Wash Samples in Immunocompromised Patients: Usefulness and Importance of the DNA Target. Journal of clinical microbiology, 2019. 57(12).

557. Goulet, D., et al., Sugar clearance from saliva and intra-oral spaces. J Dent Res, 1985. 64(3): p. 411-5.

558. Goulet, E.D., Dehydration and endurance performance in competitive athletes. Nutrition Reviews, 2012. 70: p. S132-S136.

559. Goulet, E.D.B., Dehydration and endurance performance in competitive athletes. Nutrition reviews, 2012. 70 Suppl 2: p. S132-6.

560. Gracia, L.H., et al., An in vitro evaluation of a novel high fluoride daily mouthrinse using a combination of microindentation, 3D profilometry and DSIMS. J Dent, 2010. 38 Suppl 3: p. S12-20.

561. Grases, F., et al., Anticalculus effect of a triclosan mouthwash containing phytate: a double-blind, randomized, three-period crossover trial. J Periodontal Res, 2009. 44(5): p. 616-21.

562. Grassi, D., et al., Blood pressure is reduced and insulin sensitivity increased in glucose-intolerant, hypertensive subjects after 15 days of consuming high-polyphenol dark chocolate. Journal of Nutrition, 2008. 138(9): p. 1671-1676.

563. Grassi, D., et al., Short-term administration of dark chocolate is followed by a significant increase in insulin sensitivity and a decrease in blood pressure in healthy persons. American Journal of Clinical Nutrition, 2005. 81(3): p. 611-614.

564. Graziani, F., et al., Dental plaque, gingival inflammation and tooth -discolouration with different commercial -formulations of 0.2% chlorhexidine rinse: a double-blind randomised controlled clinical trial. Oral Health Prev Dent, 2015. 13(2): p. 101-11.

565. Green, M.S., et al., Effect of Carbohydrate Mouth Rinse on Resistance Exercise Performance. J Strength Cond Res, 2020.

566. Greenstein, R.B., et al., Reduction of oral malodor by oxidizing lozenges. J Periodontol, 1997. 68(12): p. 1176-81.

567. Grenier, J., et al., Pomelo juice, but not cranberry juice, affects the pharmacokinetics of cyclosporine in humans. Clinical Pharmacology & Therapeutics, 2006. 79(3): p. 255-262.

568. Grgic, J., et al., The Influence of Caffeine Supplementation on Resistance Exercise: A Review. Sports medicine (Auckland, N.Z.), 2019. 49(1): p. 17-30.

569. Grgic, J., et al., The Influence of Caffeine Supplementation on Resistance Exercise: A Review. Sports Medicine, 2019. 49(1): p. 17-30.

570. Griffen, A.L. and S.J. Goepferd, Preventive oral health care for the infant, child, and adolescent. Pediatr Clin North Am, 1991. 38(5): p. 1209-26.

571. Grossman, R.P. and B.D. Till, The persistence of classically conditioned brand attitudes. Journal of Advertising, 1998. 27(1): p. 23-31.

572. Gu, L.S., et al., Chitosan-Based Extrafibrillar Demineralization for Dentin Bonding. J Dent Res, 2019. 98(2): p. 186-193.

573. Guangda, X., et al., Apovarepsilon(4) allele increases the risk for exercise-induced silent myocardial ischemia in non-insulin-dependent diabetes mellitus. Atherosclerosis, 1999. 147(2): p. 293-6.

574. Guarda Nardini, L., et al., [Influence of intra-articular injections of sodium hyaluronate on clinical features and synovial fluid nitric oxide levels of temporomandibular osteoarthritis]. Reumatismo, 2004. 56(4): p. 272-7.

575. Gubbins, P.O., et al., Influence of grapefruit juice on the systemic availability of itraconazole oral solution in healthy adult volunteers. Pharmacotherapy, 2004. 24(4): p. 460-467.

576. Guerret, M., P. Francheteau, and M. Hubert, Evaluation of effects of terbinafine on single oral dose pharmacokinetics and anticoagulant actions of warfarin in healthy volunteers. Pharmacotherapy, 1997. 17(4): p. 767-773.

577. Guest, N.S., et al., International society of sports nutrition position stand: caffeine and exercise performance. J Int Soc Sports Nutr, 2021. 18(1): p. 1.

578. Guimaraes, D.M., et al., Effect of Denture Adhesives in New Complete Dentures During a Function. Open Dentistry Journal, 2018. 12: p. 969-973.

579. Gulotta, C., et al., EFFECTS OF ORAL LACIDIPINE ON CARDIOPULMONARY FUNCTION AT REST AND DURING EXERCISE IN NORMAL SUBJECTS. Journal of Cardiovascular Pharmacology, 1991. 17: p. S55-S58.

580. Gunaydin, Z., A.R. Yazici, and Z.C. Cehreli, In Vivo and In Vitro Effects of Chlorhexidine Pretreatment on Immediate and Aged Dentin Bond Strengths. Operative Dentistry, 2016. 41(3): p. 258-267.

581. Gungor, A.S. and N. Donmez, Dentin erosion preventive effects of various plant extracts: An in vitro atomic force microscopy, scanning electron microscopy, and nanoindentation study. Microscopy Research and Technique, 2021. 84(5): p. 1042-1052.

582. Guo, B.N., et al., Safety and Clinical Pharmacokinetics of Nemonoxacin, a Novel Non-Fluorinated Quinolone, in Healthy Chinese Volunteers Following Single and Multiple Oral Doses. Clinical Drug Investigation, 2012. 32(7): p. 475-486.

583. Guo, C.X., et al., Effects of genetic factors on the pharmacokinetics and pharmacodynamics of amlodipine in primary hypertensive patients. Biomedical Reports, 2015. 3(2): p. 195-200.

584. Guo, J.M., et al., Polymer conjugation optimizes EDTA as a calcium-chelating agent that exclusively removes extrafibrillar minerals from mineralized collagen. Acta Biomater, 2019. 90: p. 424-440.

585. Gupta, S.K., et al., EFFECT OF ALOSETRON (A NEW 5-HT3 RECEPTOR ANTAGONIST) ON THE PHARMACOKINETICS OF HALOPERIDOL IN SCHIZOPHRENIC-PATIENTS. Journal of Clinical Pharmacology, 1995. 35(2): p. 202-207.

586. Haab, F., L. Stewart, and P. Dwyer, Darifenacin, an M-3 selective receptor antagonist, is an effective and well-tolerated once-daily treatment for overactive bladder. European Urology, 2004. 45(4): p. 420-429.

587. Hagger, M.S. and N.L. Chatzisarantis, The sweet taste of success: the presence of glucose in the oral cavity moderates the depletion of self-control resources. Pers Soc Psychol Bull, 2013. 39(1): p. 28-42.

588. Hagger, M.S. and N.L.D. Chatzisarantis, The sweet taste of success: the presence of glucose in the oral cavity moderates the depletion of self-control resources. Personality & social psychology bulletin, 2013. 39(1): p. 28-42.

589. Hagiu, A., et al., Dose-dependent green tea effect on decrease of inflammation in human oral gingival epithelial keratinocytes: in vitro study. Clin Oral Investig, 2020. 24(7): p. 2375-2383.

590. Hahnel, S., et al., Influence of saliva substitute films on the initial adhesion of Candida albicans to dental substrata prior to and after artificial ageing. Arch Oral Biol, 2010. 55(5): p. 391-6.

591. Hahnel, S., et al., Influence of saliva substitute films on initial Streptococcus mutans adhesion to enamel and dental substrata. J Dent, 2008. 36(12): p. 977-83.

592. Hallam, K.T., et al., Comparative cognitive and psychomotor effects of single doses of Valeriana officianalis and triazolam in healthy volunteers. Human Psychopharmacology-Clinical and Experimental, 2003. 18(8): p. 619-625.

593. Hallstrom, H., et al., Effect of probiotic lozenges on inflammatory reactions and oral biofilm during experimental gingivitis. Acta Odontologica Scandinavica, 2013. 71(3-4): p. 828-833.

594. Hamilton, A., The rinse method. Athletics Weekly (0004-6671), 2010. 65(16): p. 58-59.

595. Hamulati, W., Y. Aibibai, and L.J. Zhong, [Inhibitory effect of xipayi mouth rinse on the secretion of IL-6 from human gingival fibroblast induced by lipopolysaccharide]. Zhong Nan Da Xue Xue Bao Yi Xue Ban, 2006. 31(3): p. 326-8, 335.

596. Hanaki, M., et al., Glucose clearance from different surfaces of human central incisors and first molars. Arch Oral Biol, 1993. 38(6): p. 479-82.

597. Haney, M., et al., Dronabinol and marijuana in HIV-positive marijuana smokers - Caloric intake, mood, and sleep. Jaids-Journal of Acquired Immune Deficiency Syndromes, 2007. 45(5): p. 545-554.

598. Haney, M., et al., Effects of THC and lofexidine in a human laboratory model of marijuana withdrawal and relapse. Psychopharmacology, 2008. 197(1): p. 157-168.

599. Hang, T.J., et al., Simultaneous determination and pharmacokinetic study of roxithromycin and ambroxol hydrochloride in human plasma by LC-MS/MS. Clinica Chimica Acta, 2007. 382(1-2): p. 20-24.

600. Hanif, M., et al., PHARMACOKINETICS AND BIOEQUIVALENCE STUDIES OF TWO NIMESULIDE 100 mg TABLETS: UNIT DOSE, RANDOMIZED-SEQUENCE, TWO-WAY CROSSOVER STUDY IN HEALTHY VOLUNTEERS OF PAKISTANI POPULATION. Acta Poloniae Pharmaceutica, 2017. 74(2): p. 489-495.

601. Hannig, C., et al., Lysozyme activity in the initially formed in situ pellicle. Archives of Oral Biology, 2005. 50(9): p. 821-828.

602. Hannig, C., et al., Do edible oils reduce bacterial colonization of enamel in situ? Clin Oral Investig, 2013. 17(2): p. 649-58.

603. Hanning, S.M. and N.J. Medlicott, Oil-based compositions as saliva substitutes: A pilot study to investigate in-mouth retention. Int J Pharm, 2016. 501(1-2): p. 265-70.

604. Harorli, O.T. and C. Barutcigil, Color recovery effect of commercial mouth rinses on a discolored composite. Journal of esthetic and restorative dentistry : official publication of the American Academy of Esthetic Dentistry ... [et al.], 2014. 26(4): p. 256-63.

605. Harpenau, L.A., J.M. Plemons, and T.D. Rees, Effectiveness of a low dose of cyclosporine in the management of patients with oral erosive lichen planus. Oral surgery, oral medicine, oral pathology, oral radiology, and endodontics, 1995. 80(2): p. 161-7.

606. Harpenau, L.A., J.M. Plemons, and T.D. Rees, EFFECTIVENESS OF A LOW-DOSE OF CYCLOSPORINE IN THE MANAGEMENT OF PATIENTS WITH ORAL EROSIVE LICHEN-PLANUS. Oral Surgery Oral Medicine Oral Pathology Oral Radiology and Endodontology, 1995. 80(2): p. 161-167.

607. Harris, A., et al., Hemodynamic and visual function effects of oral nifedipine in patients with normal-tension glaucoma. American Journal of Ophthalmology, 1997. 124(3): p. 296-302.

608. Harrison, L.I., et al., EFFECT OF FOOD ON SALSALATE ABSORPTION. Therapeutic Drug Monitoring, 1992. 14(2): p. 87-91.

609. Hart, R.P., C.C. Colenda, and R.M. Hamer, EFFECTS OF BUSPIRONE AND ALPRAZOLAM ON THE COGNITIVE PERFORMANCE OF NORMAL ELDERLY SUBJECTS. American Journal of Psychiatry, 1991. 148(1): p. 73-77.

610. Hartmann, D., et al., Lack of interaction between orlistat and oral contraceptives. European Journal of Clinical Pharmacology, 1996. 50(5): p. 421-424.

611. Hasan, F., et al., The effects of aspirin gel and mouthwash on levels of salivary biomarkers PGE2, TNF-alpha and nitric oxide in patients with periodontal diseases. Pakistan journal of pharmaceutical sciences, 2019. 32(5): p. 2019-2023.

612. Hase, J.C. and D. Birkhed, Salivary glucose clearance, dry mouth and pH changes in dental plaque in man. Arch Oral Biol, 1988. 33(12): p. 875-80.

613. Hase, J.C. and D. Birkhed, Oral sugar clearance in elderly people with prosthodontic reconstructions. Scand J Dent Res, 1991. 99(4): p. 333-9.

614. Hase, J.C., et al., Oral retention of glucose at pharmacologically reduced salivary flow in man. Scand J Dent Res, 1994. 102(3): p. 180-5.

615. Hase, J.C., et al., An individual training programme for speeding up prolonged oral sugar clearance in hospitalized elderly patients. A pilot study. Swed Dent J, 1992. 16(6): p. 239-45.

616. Hase, J.C., et al., 6-month use of 0.2% delmopinol hydrochloride in comparison with 0.2% chlorhexidine digluconate and placebo (II). Effect on plaque and salivary microflora. Journal of clinical periodontology, 1998. 25(11 Pt 1): p. 841-9.

617. Hasegawa, S., et al., Bioequivalence of rebamipide granules and tablets in healthy adult male volunteers. Clinical Drug Investigation, 2003. 23(12): p. 771-779.

618. Hasheminasab, F.S., et al., Effects of a Plantago ovata-based herbal compound in prevention and treatment of oral mucositis in patients with breast cancer receiving chemotherapy: A double-blind, randomized, controlled crossover trial. Journal of Integrative Medicine-Jim, 2020. 18(3): p. 214-221.

619. Hassanein, K., B.T. Musgrove, and E. Bradbury, Functional status of patients with oral cancer and its relation to style of coping, social support and psychological status. British Journal of Oral & Maxillofacial Surgery, 2001. 39(5): p. 340-345.

620. Hatsukami, D.K., et al., Safety of cotinine in humans: Physiologic, subjective, and cognitive effects. Pharmacology Biochemistry and Behavior, 1997. 57(4): p. 643-650.

621. Hauptstein, S., et al., Development and in vitro evaluation of a buccal drug delivery system based on preactivated thiolated pectin. Drug Dev Ind Pharm, 2014. 40(11): p. 1530-7.

622. Haverinen, A., et al., Ethinyl estradiol vs estradiol valerate in combined oral contraceptives - Effect on glucose tolerance: A randomized, controlled clinical trial. Contraception, 2021. 103(1): p. 53-59.

623. Hawkins, K.R., et al., Running Performance With Nutritive and Nonnutritive Sweetened Mouth Rinses. International journal of sports physiology and performance, 2017. 12(8): p. 1105-1110.

624. Hayashi, T., et al., Visceral adiposity is an independent predictor of incident hypertension in Japanese Americans. Annals of Internal Medicine, 2004. 140(12): p. 992-1000.

625. Hayashi, Y., et al., Chitosan-containing gum chewing accelerates antibacterial effect with an increase in salivary secretion. J Dent, 2007. 35(11): p. 871-4.

626. Hearris, M.A., et al., Regulation of Muscle Glycogen Metabolism during Exercise: Implications for Endurance Performance and Training Adaptations. Nutrients, 2018. 10(3).

627. Heaton, A.L., et al., Mechanism for the Increase in Human Growth Hormone with Administration of a Novel Test Supplement and Results Indicating Improved Physical Fitness and Sleep Efficiency. Journal of Medicinal Food.

628. Heine, P.R., et al., LACK OF INTERACTION BETWEEN DIAZEPAM AND NIMODIPINE DURING CHRONIC ORAL-ADMINISTRATION TO HEALTHY ELDERLY SUBJECTS. British Journal of Clinical Pharmacology, 1994. 38(1): p. 39-43.

629. Helenius, I., et al., No effect of montelukast on asthma-like symptoms in elite ice hockey players. Allergy, 2004. 59(1): p. 39-44.

630. Helms, J.A., et al., Effects of chlorhexidine on human taste perception. Arch Oral Biol, 1995. 40(10): p. 913-20.

631. Helmy, S.A. and N.O. Mansour, In Vitro Dissolution and In Vivo Bioequivalence Evaluation of Two Brands of Trimetazidine Tablets. Clinical Pharmacology in Drug Development, 2014. 3(2): p. 139-143.

632. Hemelaar, M., et al., Oral, more than transdermal, estrogen therapy improves lipids and lipoprotein(a) in postmenopausal women: a randomized, placebo-controlled study. Menopause-the Journal of the North American Menopause Society, 2003. 10(6): p. 550-558.

633. Hemelaar, M., et al., Effects of transdermal and oral postmenopausal hormone therapy on vascular function: a randomized, placebo-controlled study in healthy postmenopausal women. Menopause-the Journal of the North American Menopause Society, 2005. 12(5): p. 526-535.

634. Hendley, T.M., R.B. Steed, and G.M. Galbraith, Interleukin-1 beta gene expression in human oral polymorphonuclear leukocytes. J Periodontol, 1995. 66(9): p. 761-5.

635. Henkin, R.I., M. Schultz, and L. Minnick-Poppe, Intranasal Theophylline Treatment of Hyposmia and Hypogeusia A Pilot Study. Archives of Otolaryngology-Head & Neck Surgery, 2012. 138(11): p. 1064-1070.

636. Henningfield, J.E., et al., Drinking coffee and carbonated beverages blocks absorption of nicotine from nicotine polacrilex gum. Jama, 1990. 264(12): p. 1560-4.

637. Herberg, K.W., et al., Pantoprazole does not affect performance in traffic-related safety tests - A double-blind, randomised, placebo-controlled, crossover study in healthy volunteers. Clinical Drug Investigation, 1998. 16(1): p. 63-70.

638. Hermann, G.A., et al., Variability of quantitative scintigraphic salivary indices in normal subjects. Journal of Nuclear Medicine, 1998. 39(7): p. 1260-1263.

639. Herrmann, N., et al., Methylphenidate for the treatment of apathy in Alzheimer disease: Prediction of Response Using Dextroamphetamine Challenge. Journal of Clinical Psychopharmacology, 2008. 28(3): p. 296-301.

640. Hertel, S., et al., Effect of Tannic Acid on the Protective Properties of the in situ Formed Pellicle. Caries Res, 2017. 51(1): p. 34-45.

641. Heuner, A., et al., A SINGLE-DOSE AND 3-MONTH CLINICAL-PHARMACOKINETIC STUDY WITH A NEW COMBINATION ORAL-CONTRACEPTIVE. Advances in Contraception, 1995. 11(3): p. 207-225.

642. Higham, S.M. and W.M. Edgar, Human dental plaque pH, and the organic acid and free amino acid profiles in plaque fluid, after sucrose rinsing. Arch Oral Biol, 1989. 34(5): p. 329-34.

643. Hildebrandt, G., I. Lee, and J. Hodges, Oral mutans streptococci levels following use of a xylitol mouth rinse: a double-blind, randomized, controlled clinical trial. Spec Care Dentist, 2010. 30(2): p. 53-8.

644. Hildebrandt, G.H. and B.S. Sparks, Maintaining mutans streptococci suppression with xylitol chewing gum. J Am Dent Assoc, 2000. 131(7): p. 909-16.

645. Hirano, T., et al., Inhibition of reactive nitrogen species production in COPD airways: comparison of inhaled corticosteroid and oral theophylline. Thorax, 2006. 61(9): p. 761-766.

646. Hirota, W., Intra-articular injection of hyaluronic acid reduces total amounts of leukotriene C4, 6-keto-prostaglandin F1alpha, prostaglandin F2alpha and interleukin-1beta in synovial fluid of patients with internal derangement in disorders of the temporomandibular joint. Br J Oral Maxillofac Surg, 1998. 36(1): p. 35-8.

647. Hitz Lindenmüller, I. and J.T. Lambrecht, Oral care. Curr Probl Dermatol, 2011. 40: p. 107-115.

648. Hnot, M.L., et al., Effect of feeding on the pharmacokinetics of oral minocycline in healthy research dogs. Veterinary Dermatology, 2015. 26(6): p. 399-+.

649. Ho, P.C., et al., Grapefruit juice has no effect on quinine pharmacokinetics. European Journal of Clinical Pharmacology, 1999. 55(5): p. 393-398.

650. Hodson, N.A. and R.W. Linden, The effect of monosodium glutamate on parotid salivary flow in comparison to the response to representatives of the other four basic tastes. Physiol Behav, 2006. 89(5): p. 711-7.

651. Holbrook, W.P., T. Kristmundsdottir, and T. Loftsson, Aqueous hydrocortisone mouthwash solution: clinical evaluation. Acta odontologica Scandinavica, 1998. 56(3): p. 157-60.

652. Holden, A.C.L., Cosmetic dentistry: A socioethical evaluation. Bioethics, 2018. 32(9): p. 602-610.

653. Holz, O., et al., SCH527123, a novel CXCR2 antagonist, inhibits ozone-induced neutrophilia in healthy subjects. European Respiratory Journal, 2010. 35(3): p. 564-570.

654. Hopes, H. and J. Wandmacher, A COMPARISON OF THE EFFECT OF LOFEPRAMINE, MAPROTILINE AND PLACEBO ON INFORMATION-PROCESSING IN HEALTHY-VOLUNTEERS. Human Psychopharmacology-Clinical and Experimental, 1992. 7(3): p. 183-191.

655. Hopkins, W.G., Effects Went Away at the 2013 Annual Meeting of the European College of Sport Science. Sportscience, 2013. 17: p. 1-12.

656. Hoppe, K., [Psychophysics of sweet taste. II. Statistical theory of the stimulus-response behavior of sweet taste receptors]. Nahrung, 1981. 25(2): p. 151-65.

657. Horner-Devine, A.R., et al., A conceptual model of the strongly tidal Columbia River plume. Journal of Marine Systems, 2009. 78(3): p. 460-475.

658. Hoshino, E., et al., Plaque pH recovery by mouth-rinses with water. Shika Kiso Igakkai Zasshi, 1989. 31(2): p. 218-23.

659. Hoszek, A., et al., Chlorhexidine-containing glass ionomer cement. A clinical investigation on the fissure caries inhibiting effect in first permanent molars. Swed Dent J, 2005. 29(3): p. 89-96.

660. Hou, Z.Y., et al., Pharmacokinetics and relative bioavailability of two allopurinol tablets in healthy Chinese volunteers. International Journal of Clinical Pharmacology and Therapeutics, 2017. 55(6): p. 540-546.

661. Houghton, J.W., et al., Agonists of Orally Expressed TRP Channels Stimulate Salivary Secretion and Modify the Salivary Proteome. Mol Cell Proteomics, 2020. 19(10): p. 1664-1676.

662. Houghton, J.W., et al., Sensory effects of transient receptor potential channel agonists on whole mouth saliva extensional rheology. J Texture Stud, 2017. 48(4): p. 313-317.

663. Howard, D.R., et al., Single-dose and steady-state bioequivalence of fexofenadine and pseudoephedrine combination tablets compared with individual formulations in healthy adults. Current Medical Research and Opinion, 2005. 21(5): p. 769-775.

664. Hsieh, J.Y.K., L. Lin, and B.K. Matuszewski, High-throughput liquid chromatographic determination of rofecoxib in human plasma using a fully automated on-line solid-phase extraction system. Journal of Liquid Chromatography & Related Technologies, 2001. 24(6): p. 799-812.

665. Hu, O.Y.P., et al., ABSORPTION STUDY WITH A DISSOLUTION INDEPENDENT DRUG - ACETAMINOPHEN. Journal of Food and Drug Analysis, 1995. 3(3): p. 155-162.

666. Huang, X.M., et al., Bioequivalence of two quetiapine extended release tablets in Chinese healthy volunteers under fasting and fed conditions and effects of food on pharmacokinetic profiles. Drug Design Development and Therapy, 2019. 13: p. 255-264.

667. Huang, Z., et al., Use of experimental-resin-based materials doped with carboxymethyl chitosan and calcium phosphate microfillers to induce biomimetic remineralization of caries-affected dentin. J Mech Behav Biomed Mater, 2019. 89: p. 81-88.

668. Huestis, M.A., et al., Estimating time of last oral ingestion of cannabis from plasma THC and THCCOOH concentrations. Therapeutic Drug Monitoring, 2006. 28(4): p. 540-544.

669. Hui, F.R., et al., Improvement in inner retinal function in glaucoma with nicotinamide (vitaminB3) supplementation: A crossover randomized clinical trial. Clinical and Experimental Ophthalmology, 2020. 48(7): p. 903-914.

670. Hung, M.J., et al., Effects of verapamil in normal elderly individuals with left ventricular diastolic dysfunction. Echocardiography-a Journal of Cardiovascular Ultrasound and Allied Techniques, 2001. 18(2): p. 123-129.

671. Hunter, K.D., et al., Fluconazole-resistant Candida species in the oral flora of fluconazole-exposed HIV-positive patients. Oral Surg Oral Med Oral Pathol Oral Radiol Endod, 1998. 85(5): p. 558-64.

672. Hunter, L., et al., A study of a pre-brushing mouthrinse as an adjunct to oral hygiene. Journal of periodontology, 1994. 65(8): p. 762-5.

673. Hussein, I., M.A. Pollard, and M.E. Curzon, A comparison of the effects of some extrinsic and intrinsic sugars on dental plaque pH. Int J Paediatr Dent, 1996. 6(2): p. 81-6.

674. Ibrahim, F., et al., Probiotics and immunosenescence: cheese as a carrier. Fems Immunology and Medical Microbiology, 2010. 59(1): p. 53-59.

675. Idayu Mat Nawi, R., et al., Oral Cryotherapy: Prevention of Oral Mucositis and Pain Among Patients With Colorectal Cancer Undergoing Chemotherapy. Clinical journal of oncology nursing, 2018. 22(5): p. 555-560.

676. Immonen, E., et al., Randomized controlled and double-blinded study of Caphosol versus saline oral rinses in pediatric patients with cancer. Pediatric blood & cancer, 2020. 67(10): p. e28520.

677. Ionta, F.Q., et al., Effect of vegetable oils applied over acquired enamel pellicle on initial erosion. Journal of applied oral science : revista FOB, 2017. 25(4): p. 420-426.

678. Ionta, F.Q., et al., Effect of vegetable oils applied over acquired enamel pellicle on initial erosion. Journal of Applied Oral Science, 2017. 25(4): p. 420-426.

679. Ishikawa, T., et al., The amino acid-rich elemental diet Elental R preserves lean body mass during chemo- or chemoradiotherapy for esophageal cancer. Oncology reports, 2016. 36(2): p. 1093-100.

680. Isogai, A., et al., Use of fluoridated dentifrice and glucose retention at the approximal areas of anterior teeth. ASDC J Dent Child, 2001. 68(1): p. 42-6, 12.

681. Ispoglou, T., et al., Mouth Rinsing With Carbohydrate Solutions at the Postprandial State Fail to Improve Performance During Simulated Cycling Time Trials. Journal of strength and conditioning research, 2015. 29(8): p. 2316-25.

682. Ispoglou, T., et al., MOUTH RINSING WITH CARBOHYDRATE SOLUTIONS AT THE POSTPRANDIAL STATE FAIL TO IMPROVE PERFORMANCE DURING SIMULATED CYCLING TIME TRIALS. Journal of Strength & Conditioning Research, 2015. 29(8): p. 2316-2325.

683. Ispoglou, T., et al., Mouth Rinsing With Carbohydrate Solutions at the Postprandial State Fail to Improve Performance During Simulated Cycling Time Trials. J Strength Cond Res, 2015. 29(8): p. 2316-25.

684. Ito, M.K., Effects of extensive and poor gastrointestinal metabolism on the pharmacodynamics of pravastatin. Journal of Clinical Pharmacology, 1998. 38(4): p. 331-336.

685. Iturrino, J., et al., Acute Effects of a Glucagon-Like Peptide 2 Analogue, Teduglutide, on Gastrointestinal Motor Function and Permeability in Adult Patients With Short Bowel Syndrome on Home Parenteral Nutrition. Journal of Parenteral and Enteral Nutrition, 2016. 40(8): p. 1089-1095.

686. Iwata, C., et al., Daily use of dentifrice with and without xylitol and fluoride: effect on glucose retention in humans in vivo. Arch Oral Biol, 2003. 48(5): p. 389-95.

687. Iyer, G.R. and D.R. Taft, Determination of methazolamide concentrations in human biological fluids using high performance liquid chromatography. Journal of Pharmaceutical and Biomedical Analysis, 1998. 16(6): p. 1021-1027.

688. Izquierdo, I., et al., Comparative bioavailability study of triflusal oral solution vs. triflusal capsules in healthy subjects A single, randomized, two-way cross-over, open-label phase I study. Arzneimittelforschung-Drug Research, 2010. 60(1): p. 36-41.

689. Jackson, C.L., Comparison between electric toothbrushing and manual toothbrushing, with and without oral irrigation, for oral hygiene of orthodontic patients. American journal of orthodontics and dentofacial orthopedics : official publication of the American Association of Orthodontists, its constituent societies, and the American Board of Orthodontics, 1991. 99(1): p. 15-20.

690. Jacobs, P.L., et al., Oral creatine supplementation enhances upper extremity work capacity in persons with cervical-level spinal cord injury. Archives of Physical Medicine and Rehabilitation, 2002. 83(1): p. 19-23.

691. Jadert, C., et al., Decreased leukocyte recruitment by inorganic nitrate and nitrite in microvascular inflammation and NSAID-induced intestinal injury. Free radical biology & medicine, 2012. 52(3): p. 683-692.

692. Jagtap, A.G. and S.G. Karkera, Potential of the aqueous extract of Terminalia chebula as an anticaries agent. J Ethnopharmacol, 1999. 68(1-3): p. 299-306.

693. James, R.M., et al., No Dose Response Effect of Carbohydrate Mouth Rinse on Cycling Time-Trial Performance. International journal of sport nutrition and exercise metabolism, 2017. 27(1): p. 25-31.

694. Jansat, J.M., et al., Effect of food intake on the bioavailability of almotriptan, an antimigraine compound, in healthy volunteers: an open, randomized, crossover, single-dose clinical trial. International Journal of Clinical Pharmacology and Therapeutics, 2006. 44(4): p. 185-190.

695. Javadzadeh, A., et al., Efficacy of Clobetasol, Ketoconazole and Amitryptiline Mouthwash on Oral Lichen Planus. Iranian Journal of Pharmaceutical Research, 2008. 7(3): p. 171-178.

696. Jeffers, R., et al., The effect of a carbohydrate mouth-rinse on neuromuscular fatigue following cycling exercise. Applied physiology, nutrition, and metabolism = Physiologie appliquee, nutrition et metabolisme, 2015. 40(6): p. 557-64.

697. Jensen, M., et al., Effect of Carbohydrate Mouth Rinse on Performance after Prolonged Submaximal Cycling. Medicine and science in sports and exercise, 2018. 50(5): p. 1031-1038.

698. Jensen, M., et al., Effect of Carbohydrate Mouth Rinse on Performance after Prolonged Submaximal Cycling. Medicine & Science in Sports & Exercise, 2018. 50(5): p. 1031-1038.

699. Jensen, M., T. Stellingwerff, and M. Klimstra, Carbohydrate Mouth Rinse Counters Fatigue Related Strength Reduction. International journal of sport nutrition and exercise metabolism, 2015. 25(3): p. 252-61.

700. Jensen, M.E., P.J. Polansky, and C.F. Schachtele, Plaque sampling and telemetry for monitoring acid production on human buccal tooth surfaces. Arch Oral Biol, 1982. 27(1): p. 21-31.

701. Jensen, M.E. and C.F. Schachtele, Plaque pH measurements by different methods on the buccal and approximal surfaces of human teeth after a sucrose rinse. J Dent Res, 1983. 62(10): p. 1058-61.

702. Jeukendrup, A., The New Carbohydrate Intake Recommendations, in Nutritional Coaching Strategy to Modulate Training Efficiency, K.D. Tipton and L.J.C. VanLoon, Editors. 2013. p. 63-71.

703. Jeukendrup, A., A step towards personalized sports nutrition: carbohydrate intake during exercise. Sports medicine (Auckland, N.Z.), 2014. 44 Suppl 1: p. S25-33.

704. Jeukendrup, A.E., Oral carbohydrate rinse: placebo or beneficial? Current sports medicine reports, 2013. 12(4): p. 222-7.

705. Jeukendrup, A.E. and E.S. Chambers, Oral carbohydrate sensing and exercise performance. Curr Opin Clin Nutr Metab Care, 2010. 13(4): p. 447-51.

706. Jiang, L., et al., Dynamic analysis of oral Candida carriage, distribution, and antifungal susceptibility in HIV-infected patients during the first year of highly active antiretroviral therapy in Guangxi, China. Journal of oral pathology & medicine : official publication of the International Association of Oral Pathologists and the American Academy of Oral Pathology, 2014. 43(9): p. 696-703.

707. Johnson, N., et al., Rapid assessment of salivary MMP-8 and periodontal disease using lateral flow immunoassay. Oral diseases, 2016. 22(7): p. 681-7.

708. Joiner, A., et al., Enhanced enamel benefits from a novel fluoride toothpaste. International Dental Journal, 2009. 59(4): p. 244-253.

709. Jordao, L.M.R., D.C. Malta, and M.D.M. Freire, Clustering patterns of oral and general health-risk behaviours in Brazilian adolescents: Findings from a national survey. Community Dentistry and Oral Epidemiology, 2018. 46(2): p. 194-202.

710. Jose, A., et al., A randomized clinical trial in subjects with dry mouth evaluating subjective perceptions of an experimental oral gel, an oral rinse and a mouth spray compared to water. American journal of dentistry, 2016. 29(1): p. 58-64.

711. Jose, A., et al., A randomized controlled study to evaluate an experimental moisturizing mouthwash formulation in participants experiencing dry mouth symptoms. Oral surgery, oral medicine, oral pathology and oral radiology, 2018. 126(3): p. 231-239.e5.

712. Joshipura, K., et al., Over-the-counter mouthwash use, nitric oxide and hypertension risk. Blood pressure, 2020. 29(2): p. 103-112.

713. Joshipura, K.J., et al., Over-the-counter mouthwash use and risk of pre-diabetes/diabetes. Nitric oxide : biology and chemistry, 2017. 71: p. 14-20.

714. Jouhar, R., et al., Association of BMI, Diet, Physical Activity, and Oral Hygiene Practices with DMFT Index of Male Dental Students at King Faisal University, Al-Ahsa. Nutrients, 2021. 13(1).

715. Jovanovic, D., et al., A randomized, open-label pharmacokinetic comparison of two oral formulations of fluconazole 150 mg in healthy adult volunteers. Clinical Therapeutics, 2005. 27(10): p. 1588-1595.

716. Jukic, A.M.Z., A.Z. Steiner, and D.D. Baird, Association between serum 25-hydroxyvitamin D and ovarian reserve in premenopausal women. Menopause-the Journal of the North American Menopause Society, 2015. 22(3): p. 312-316.

717. Jung, Y.-J., et al., A novel RUNX2 mutation in exon 8, G462X, in a patient with Cleidocranial Dysplasia. Journal of cellular biochemistry, 2018. 119(1): p. 1152-1162.

718. Jung, Y.J., et al., A novel RUNX2 mutation in exon 8, G462X, in a patient with Cleidocranial Dysplasia. Journal of Cellular Biochemistry, 2018. 119(1): p. 1152-1162.

719. Jungell, P. and M. Malmstrom, Cyclosporin A mouthwash in the treatment of oral lichen planus. International journal of oral and maxillofacial surgery, 1996. 25(1): p. 60-2.

720. Juntunen, K.S., et al., High-fiber rye bread and insulin secretion and sensitivity in healthy postmenopausal women. American Journal of Clinical Nutrition, 2003. 77(2): p. 385-391.

721. Jurema, A.L., et al., Effect of Over-the-counter Whitening Products associated or Not with 10% Carbamide Peroxide on Color Change and Microhardness: in vitro Study. The journal of contemporary dental practice, 2018. 19(4): p. 359-366.

722. Jurevic, R.J., et al., Identification of gentian violet concentration that does not stain oral mucosa, possesses anti-candidal activity and is well tolerated. European Journal of Clinical Microbiology & Infectious Diseases, 2011. 30(5): p. 629-633.

723. Kalfas, S. and S. Edwardsson, Sorbitol-fermenting predominant cultivable flora of human dental plaque in relation to sorbitol adaptation and salivary secretion rate. Oral Microbiol Immunol, 1990. 5(1): p. 33-8.

724. Kamaruddin, H.K., et al., Carbohydrate Mouth Rinse Enhances Time to Exhaustion of Running Performance Among Dehydrated Subjects, in 3rd International Conference on Movement, Health and Exercise: Engineering Olympic Success: From Theory to Practice, F. Ibrahim, et al., Editors. 2017. p. 121-128.

725. Kamaruddin, H.K., et al., The ergogenic potency of carbohydrate mouth rinse on endurance running performance of dehydrated athletes. European journal of applied physiology, 2019. 119(8): p. 1711-1723.

726. Kamaruddin, H.K., et al., The ergogenic potency of carbohydrate mouth rinse on endurance running performance of dehydrated athletes. European Journal of Applied Physiology, 2019. 119(8): p. 1711-1723.

727. Kamolnarumeth, K., et al., Effect of mixed chlorhexidine and hydrogen peroxide mouthrinses on developing plaque and stain in gingivitis patients: a randomized clinical trial. Clinical oral investigations, 2021. 25(4): p. 1697-1704.

728. Kamonpatana, K., et al., Susceptibility of anthocyanins to ex vivo degradation in human saliva. Food Chemistry, 2012. 135(2): p. 738-747.

729. Kana, K., et al., Mouth rinsing with a carbohydrate solution attenuates exercise-induced decline in executive function. Journal of the International Society of Sports Nutrition, 2017. 14: p. 1-8.

730. Kang, Y.S. and Y.J. Chang, Using a motion-controlled game to teach four elementary school children with intellectual disabilities to improve hand hygiene. Journal of Applied Research in Intellectual Disabilities, 2019. 32(4): p. 942-951.

731. Kannankeril, P.J., et al., Efficacy of Flecainide in the Treatment of Catecholaminergic Polymorphic Ventricular Tachycardia A Randomized Clinical Trial. Jama Cardiology, 2017. 2(7): p. 759-766.

732. Kao, C.H. and Y.Y. Shen, Misdiagnosis of laryngeal cancer resulting from FDG secretion in saliva. Clin Nucl Med, 2003. 28(9): p. 794.

733. Kaposvári, I., et al., [Prospective randomized study regarding the effect of the preoperative antibiotic and chlorhexidine rinse on wound healing after mandibular third molar surgery]. Orv Hetil, 2017. 158(1): p. 13-19.

734. Karayigit, R., et al., Different Doses of Carbohydrate Mouth Rinse Have No Effect on Exercise Performance in Resistance Trained Women. Int J Environ Res Public Health, 2021. 18(7).

735. Karayigit, R., et al., Different Doses of Carbohydrate Mouth Rinse Have No Effect on Exercise Performance in Resistance Trained Women. International journal of environmental research and public health, 2021. 18(7).

736. Karayigit, R., et al., Different Doses of Carbohydrate Mouth Rinse Have No Effect on Exercise Performance in Resistance Trained Women. International Journal of Environmental Research and Public Health, 2021. 18(7).

737. Karayiğit, R., et al., HIGH DOSE OF SERIAL CARBOHYDRATE MOUTH RINSE IMPROVES SPRINT CYCLING PERFORMANCE IN FEMALE ATHLETES. Congress Papers of The Association of Sports Sciences, 2019: p. 1560-1560.

738. KarayİĞİT, R., et al., EFFECT OF SERIAL CAFFEINE MOUTH RINSE ON WINGATE ANAEROBIC PERFORMANCE. Journal of Physical Education & Sports Sciences / Ankara Üniversitesi Beden Egitimi ve Spor Yüksekokulu SPORMETRE Beden Eitimi ve Spor Bilimleri Dergisi, 2017. 15(4): p. 191-196.

739. Karayiğit, R., et al., KARBONHİDRATIN AĞIZDA ÇALKALANMASININ KADIN SPORCULARDA KUVVET VE KASSAL DAYANIKLILIK PERFORMANSINA ETKİSİ. / THE EFFECT OF CARBOHYDRATE MOUTH RINSE ON STRENGTH AND MUSCULAR ENDURANCE PERFORMANCE IN FEMALE ATHLETES. Congress Papers of The Association of Sports Sciences, 2019: p. 3191-3191.

740. Karim, A.C., et al., The effects of adriamycin on dental proteins formation and mineralization in vitro. Exp Toxicol Pathol, 1993. 45(1): p. 41-6.

741. Karpecka, E. and B. Fraczek, Macronutrients and water - do they matter in the context of cognitive performance in athletes? Baltic Journal of Health and Physical Activity, 2020. 12(3): p. 103-124.

742. Karuk, H.N., G.R. Nalcakan, and E. Pekunlu, Effects of carbohydrate and caffeine combination mouth rinse on anaerobic performance of highly trained male athletes. European Journal of Sport Science.

743. Karuk, H.N., G. Rudarli Nalcakan, and E. Pekünlü, Effects of carbohydrate and caffeine combination mouth rinse on anaerobic performance of highly trained male athletes. Eur J Sport Sci, 2021: p. 1-11.

744. Kashima, H., et al., Suppression of sweet sensing with glucose, but not aspartame, delays gastric emptying and glycemic response. Nutr Res, 2019. 68: p. 62-69.

745. Kashima, N., et al., Suppression of Oral Sweet Sensations during Consumption of Sweet Food in Humans: Effects on Gastric Emptying Rate, Glycemic Response, Appetite, Food Satisfaction and Desire for Basic Tastes. Nutrients, 2020. 12(5).

746. Kashket, S. and L.R. Lopez, Reduction of intra-oral demineralization of enamel after single exposures to sodium fluoride. J Dent Res, 1992. 71 Spec No: p. 867-70.

747. Kashket, S. and T. Yaskell, Accumulation of enamel constituents in Streptococcus mutans plaque during intraoral demineralization. Caries Res, 1990. 24(4): p. 248-53.

748. Kashket, S. and T. Yaskell, Effect of timing of administered calcium lactate on the sucrose-induced intraoral demineralization of bovine enamel. Arch Oral Biol, 1992. 37(3): p. 187-91.

749. Kashket, S. and T. Yaskell, Limitations in the intraoral demineralization of bovine enamel. Caries Res, 1992. 26(2): p. 98-103.

750. Kashket, S. and T. Yaskell, Effectiveness of calcium lactate added to food in reducing intraoral demineralization of enamel. Caries Res, 1997. 31(6): p. 429-33.

751. Kashket, S., T. Yaskell, and L.R. Lopez, Prevention of sucrose-induced demineralization of tooth enamel by chewing sorbitol gum. J Dent Res, 1989. 68(3): p. 460-2.

752. Kasper, A.M., et al., Carbohydrate mouth rinse and caffeine improves high-intensity interval running capacity when carbohydrate restricted. European journal of sport science, 2016. 16(5): p. 560-8.

753. Kato, I., et al., Nutritional Correlates of Human Oral Microbiome. Journal of the American College of Nutrition, 2017. 36(2): p. 88-98.

754. Kato, I., et al., Nutritional Correlates of Human Oral Microbiome. Journal of the American College of Nutrition, 2017. 36(2): p. 88-98.

755. Kawano, Y., et al., Development and Characterization of Oral Spray for Stomatitis Containing Irsogladine Maleate. Chemical & pharmaceutical bulletin, 2016. 64(12): p. 1659-1665.

756. Kawasaki, K., Y. Kamikawa, and K. Sugihara, In vitro and in vivo removal of oral Candida from the denture base. Gerodontology, 2016. 33(2): p. 247-252.

757. Kay, G.G. and K.A. Wesnes, Pharmacodynamic effects of darifenacin, a muscarinic M-3 selective receptor antagonist for the treatment of overactive bladder, in healthy volunteers. Bju International, 2005. 96(7): p. 1055-1062.

758. Keane, J., et al., The Performance Effect of Scheduled Carbohydrate and Caffeine Intake during Simulated Team Sport Match-Play. Nutrients, 2020. 12(7).

759. Keane, W.F., et al., Rabeprazole: Pharmacokinetics and tolerability in patients with stable, end-stage renal failure. Journal of Clinical Pharmacology, 1999. 39(9): p. 927-933.

760. Keller, G.A., et al., Comparative Bioavailability of 2 Tablet Formulations of Levodopa/Benserazide in Healthy, Fasting Volunteers: A Single-Dose, Randomized-Sequence, Open-Label Crossover Study. Clinical Therapeutics, 2011. 33(4): p. 500-510.

761. Keller, M.K., et al., Effect of chewing gums containing the probiotic bacterium Lactobacillus reuteri on oral malodour. Acta Odontologica Scandinavica, 2012. 70(3): p. 246-250.

762. Kemmeren, J.M., A. Algra, and D.E. Grobbee, Effect of second and third generation oral contraceptives on lipid metabolism in the absence or presence of the factor V Leiden mutation. Journal of Internal Medicine, 2001. 250(5): p. 441-448.

763. Kemmeren, J.M., et al., Effect of second- and third-generation oral contraceptives on fibrinolysis in the absence or presence of the factor V Leiden mutation. Blood Coagulation & Fibrinolysis, 2002. 13(5): p. 373-381.

764. Kemmeren, J.M., et al., Effects of second and third generation oral contraceptives and their respective progestagens on the coagulation system in the absence or presence of the factor V Leiden mutation. Thrombosis and Haemostasis, 2002. 87(2): p. 199-205.

765. Kemmeren, J.M., et al., Effect of second- and third-generation oral contraceptives on the protein C system in the absence or presence of the factor V-Leiden mutation: a randomized. Blood, 2004. 103(3): p. 927-933.

766. Ker, K., D. Beecher, and I. Roberts, Topical application of tranexamic acid for the reduction of bleeding. Cochrane Database of Systematic Reviews, 2013(7).

767. Keranen, T., et al., Effects of charcoal on the absorption and elimination of the antiepileptic drugs lamotrigine and oxcarbazepine. Arzneimittel-Forschung-Drug Research, 2010. 60(7): p. 421-426.

768. Khan, A., et al., Bioequivance Study of Newly Developed Metoclopramide HCl Tablets in Healthy Male Pakistani Volunteers. Latin American Journal of Pharmacy, 2015. 34(1): p. 153-160.

769. Khong, T.K., V. Selvanayagam, and A. Yusof, Effect of glucose and sodium chloride mouth rinses on neuromuscular fatigue: a preliminary study. European Journal of Sport Science, 2021. 21(2): p. 224-230.

770. Khoroushi, M. and S. Aghelinejad, Effect of postbleaching application of an antioxidant on enamel bond strength of three different adhesives. Med Oral Patol Oral Cir Bucal, 2011. 16(7): p. e990-6.

771. Kim, E.H., et al., Prediction of Chronic Periodontitis Severity Using Machine Learning Models Based On Salivary Bacterial Copy Number. Frontiers in Cellular and Infection Microbiology, 2020. 10.

772. Kim, H., J.H. Bae, and W.S. Chung, Effects of a chattering teeth training oral appliance for working memory improvement in healthy volunteers: a cross-over randomized trial. Integrative Medicine Research, 2019. 8(4): p. 247-251.

773. Kim, H.S., et al., Effect of voglibose on the pharmacokinetics of metformin in healthy Korean subjects. International Journal of Clinical Pharmacology and Therapeutics, 2014. 52(11): p. 1005-1011.

774. Kim, J. and E.-K. Kim, Nutritional Strategies to Optimize Performanceand Recovery in Rowing Athletes. Nutrients, 2020. 12(6).

775. Kim, J. and E.K. Kim, Nutritional Strategies to Optimize Performance and Recovery in Rowing Athletes. Nutrients, 2020. 12(6).

776. Kim, K.J., et al., The Partial Pressure of Inspired Carbon Dioxide Exposure Levels in the Extravehicular Mobility Unit. Aerospace Medicine and Human Performance, 2020. 91(12): p. 923-931.

777. Kim, M.J., et al., Comparative Pharmacokinetics and Bioavailability of Gemifloxacin Administered as an Intravenous 200 mg Formulation or an Oral 320 mg Tablet. Clinical Drug Investigation, 2014. 34(3): p. 195-201.

778. Kim, M.K., C.H. Nightingale, and D.P. Nicolau, Influence of sex on the pharmacokinetic interaction of fleroxacin and ciprofloxacin with caffeine. Clinical Pharmacokinetics, 2003. 42(11): p. 985-996.

779. Kim, S.D., et al., Bioequivalence and Tolerability of Two Clopidogrel Salt Preparations, Besylate and Bisulfate: A Randomized, Open-Label, Crossover Study in Healthy Korean Male Subjects. Clinical Therapeutics, 2009. 31(4): p. 793-803.

780. Kim, T.E., et al., Effect of food on the pharmacokinetics of the oral phosphodiesterase 5 inhibitor udenafil for the treatment of erectile dysfunction. British Journal of Clinical Pharmacology, 2009. 68(1): p. 43-46.

781. Kim, Y.C., et al., Comparative bioavailability of silibinin in healthy male volunteers. International Journal of Clinical Pharmacology and Therapeutics, 2003. 41(12): p. 593-596.

782. Kim, Y.G., et al., Bioequivalence of two aceclofenac tablet formulations after a single oral dose to healthy male Korean volunteers. International Journal of Clinical Pharmacology and Therapeutics, 2001. 39(2): p. 83-88.

783. Kim, Y.G., et al., Bioequivalence assessment of closerin capsule to Dura seromycin capsule of cycloserine after a single oral dose administration to healthy male volunteers. International Journal of Clinical Pharmacology and Therapeutics, 2000. 38(10): p. 461-466.

784. Kim, Y.K., et al., A comparative pharmacokinetic and tolerability analysis of the novel orotic acid salt form of tenofovir disoproxil and the fumaric acid salt form in healthy subjects. Drug Design Development and Therapy, 2017. 11: p. 3171-3177.

785. Kimoto, M., et al., A role of salivary carbonic anhydrase VI in dental plaque. Arch Oral Biol, 2006. 51(2): p. 117-22.

786. King, G., A. Byrne, and P. Fleming, A case of severe NSAID exacerbated respiratory disease (NERD) following a dental procedure in a child. European archives of paediatric dentistry : official journal of the European Academy of Paediatric Dentistry, 2016. 17(4): p. 277-81.

787. Kirchner, J.C., S.C. Edberg, and C.T. Sasaki, The use of topical oral antibiotics in head and neck prophylaxis: is it justified? Laryngoscope, 1988. 98(1): p. 26-9.

788. Kirsch, J., et al., Influence of pure fluorides and stannous ions on the initial bacterial colonization in situ. Sci Rep, 2019. 9(1): p. 18499.

789. Kirwan, L.D., et al., Acute and chronic effects of hormone replacement therapy on the cardiovascular system in healthy postmenopausal women. Journal of Clinical Endocrinology & Metabolism, 2004. 89(4): p. 1618-1629.

790. Kizzi, J., et al., Influence of a caffeine mouth rinse on sprint cycling following glycogen depletion. European journal of sport science, 2016. 16(8): p. 1087-94.

791. Kleinberg, I. and D.M. Codipilly, Cysteine challenge testing: a powerful tool for examining oral malodour processes and treatments in vivo. Int Dent J, 2002. 52 Suppl 3: p. 221-8.

792. Kleinloog, J.P.D., et al., Aerobic Exercise Training Improves Cerebral Blood Flow and Executive Function: A Randomized, Controlled Cross-Over Trial in Sedentary Older Men. Frontiers in Aging Neuroscience, 2019. 11.

793. Klimacka-Nawrot, E., W. Suchecka, and B. Błońska-Fajfrowska, [Gustometry usefulness for the evaluation of taste sense efficiency. Part I. The range of taste substances concentrations and the result of gustometry examination]. Wiad Lek, 2007. 60(9-10): p. 409-14.

794. Kocoloski, G.M. and A.R. Crecelius, Effects of Single-Dose Dietary Nitrate on Oxygen Consumption During and After Maximal and Submaximal Exercise in Healthy Humans: A Pilot Study. International Journal of Exercise Science, 2018. 11(3): p. 214-225.

795. Koller, M.M., et al., An analysis of submandibular salivary gland function with desipramine and age in female NIA Fischer 344 rats. Mechanisms of Ageing and Development, 2000. 119(3): p. 131-147.

796. Koller, M.M., et al., An analysis of parotid salivary gland function with desipramine and age in female NIA Fischer 344 rats. Experimental Gerontology, 2001. 36(1): p. 141-157.

797. Koller, M.M., et al., Desipramine induced changes in salivary proteins, cultivable oral microbiota and gingival health in aging female NIA Fischer 344 rats. Life Sciences, 2000. 68(4): p. 445-455.

798. Komariah, A., et al., Effects Chitosan and Calcium Nanoparticles Mouthwash from Xylotrophes gideon In the Liver And Kidney Rat. Research Journal of Pharmaceutical Biological and Chemical Sciences, 2017. 8: p. 1-9.

799. Kondo, W., et al., Laparoscopic Double Discoid Resection With a Circular Stapler for Bowel Endometriosis. Journal of Minimally Invasive Gynecology, 2015. 22(6): p. 929-931.

800. Kong, K., et al., Effect of phytic acid etchant on resin-dentin bonding: Monomer penetration and stability of dentin collagen. J Prosthodont Res, 2017. 61(3): p. 251-258.

801. Kong, K., et al., Effect of phytic acid etchant on the structural stability of demineralized dentine and dentine bonding. J Mech Behav Biomed Mater, 2015. 48: p. 145-152.

802. Konishi, K., et al., Mouth rinsing with a carbohydrate solution attenuates exercise-induced decline in executive function. Journal of the International Society of Sports Nutrition, 2017. 14: p. 45.

803. Konstan, M.W., et al., Effect of ibuprofen on neutrophil migration in vivo in cystic fibrosis and healthy subjects. Journal of Pharmacology and Experimental Therapeutics, 2003. 306(3): p. 1086-1091.

804. Kornblum, N., et al., Randomized Phase II Trial of Fulvestrant Plus Everolimus or Placebo in Postmenopausal Women With Hormone Receptor-Positive, Human Epidermal Growth Factor Receptor 2-Negative Metastatic Breast Cancer Resistant to Aromatase Inhibitor Therapy: Results of PrE0102. Journal of clinical oncology : official journal of the American Society of Clinical Oncology, 2018. 36(16): p. 1556-1563.

805. Kornblum, N., et al., Randomized Phase II Trial of Fulvestrant Plus Everolimus or Placebo in Postmenopausal Women With Hormone Receptor-Positive, Human Epidermal Growth Factor Receptor 2-Negative Metastatic Breast Cancer Resistant to Aromatase Inhibitor Therapy: Results of PrE0102. Journal of Clinical Oncology, 2018. 36(16): p. 1556-+.

806. Koschier, F., et al., In vitro effects of ethanol and mouthrinse on permeability in an oral buccal mucosal tissue construct. Food Chem Toxicol, 2011. 49(10): p. 2524-9.

807. Kovarik, J.M., et al., Pharmacokinetic and pharmacodynamic assessments of HMG-CoA reductase inhibitors when coadministered with everolimus. Journal of Clinical Pharmacology, 2002. 42(2): p. 222-228.

808. Kovarik, J.M., et al., Differential influence of two cyclosporine formulations on everolimus pharmacokinetics: A clinically relevant pharmacokinetic interaction. Journal of Clinical Pharmacology, 2002. 42(1): p. 95-99.

809. Kovarik, J.M., et al., Pharmacokinetics of dexamethasone and valspodar, a P-glycoprotein (mdr1) modulator: Implications for coadministration. Pharmacotherapy, 1998. 18(6): p. 1230-1236.

810. Kramer, N., et al., Nanohybrid vs. fine hybrid composite in Class II cavities: Clinical results and margin analysis after four years. Dental Materials, 2009. 25(6): p. 750-759.

811. Kreuzig, F. and G. Nahler, Salivary levels of gramicidin after use of a tyrothricin lozenge and a tyrothricin gargle/mouth-wash. International journal of clinical pharmacology research, 1983. 3(2): p. 65-70.

812. Krieger, J.W., M. Crowe, and S.E. Blank, Chronic glutamine supplementation increases nasal but not salivary IgA during 9 days of interval training. Journal of Applied Physiology, 2004. 97(2): p. 585-591.

813. Krings, B., et al., Effects of Carbohydrate Mouth Rinsing on Upper Body Resistance Exercise Performance. International Journal of Sport Nutrition and Exercise Metabolism, 2020. 30(1): p. 42-47.

814. Krings, B.M., et al., Effects of Carbohydrate Ingestion and Carbohydrate Mouth Rinse on Repeat Sprint Performance. International journal of sport nutrition and exercise metabolism, 2017. 27(3): p. 204-212.

815. Krings, B.M., et al., Effects of acute carbohydrate ingestion on anaerobic exercise performance. Journal of the International Society of Sports Nutrition, 2016. 13.

816. Krings, B.M., et al., Effects of Carbohydrate Mouth Rinsing on Upper Body Resistance Exercise Performance. International Journal of Sport Nutrition & Exercise Metabolism, 2020. 30(1): p. 42-47.

817. Krishna, G., et al., Effects of Oral Posaconazole on the Pharmacokinetic Properties of Oral and Intravenous Midazolam: A Phase I, Randomized, Open-Label, Crossover Study in Healthy Volunteers. Clinical Therapeutics, 2009. 31(2): p. 286-298.

818. Krishnan, V., et al., Development and evaluation of two PVD-coated beta-titanium orthodontic archwires for fluoride-induced corrosion protection. Acta biomaterialia, 2011. 7(4): p. 1913-27.

819. Kubota, C., et al., Association between chewing-stimulated salivary flow under the effects of atropine and mixing ability assessed using a color-changeable chewing gum. Journal of Prosthodontic Research, 2017. 61(4): p. 387-392.

820. Kudolo, G.B., D. Delaney, and J. Blodgett, Short-term oral ingestion of Ginkgo biloba extract (EGb 761) reduces malondialdehyde levels in washed platelets of type 2 diabetic subjects. Diabetes Research and Clinical Practice, 2005. 68(1): p. 29-38.

821. Kuhnz, W., G. Alyacoub, and A. Fuhrmeister, PHARMACOKINETICS OF LEVONORGESTREL IN 12 WOMEN WHO RECEIVED A SINGLE ORAL DOSE OF 0.15 MG LEVONORGESTREL AND, AFTER A WASH-OUT PHASE, THE SAME DOSE DURING ONE TREATMENT CYCLE. Contraception, 1992. 46(5): p. 443-454.

822. Kuhnz, W., G. Alyacoub, and A. Fuhrmeister, PHARMACOKINETICS OF LEVONORGESTREL AND ETHINYLESTRADIOL IN 9 WOMEN WHO RECEIVED A LOW-DOSE ORAL-CONTRACEPTIVE OVER A TREATMENT PERIOD OF 3 MONTHS AND, AFTER A WASH-OUT PHASE, A SINGLE ORAL-ADMINISTRATION OF THE SAME CONTRACEPTIVE FORMULATION. Contraception, 1992. 46(5): p. 455-469.

823. Kuhnz, W., et al., PHARMACOKINETICS OF GESTODENE AND ETHINYLESTRADIOL IN 14 WOMEN DURING 3 MONTHS OF TREATMENT WITH A NEW TRI-STEP COMBINATION ORAL-CONTRACEPTIVE - SERUM-PROTEIN BINDING OF GESTODENE AND INFLUENCE OF TREATMENT ON FREE AND TOTAL TESTOSTERONE LEVELS IN THE SERUM. Contraception, 1993. 48(4): p. 303-322.

824. Kuhnz, W., C. Gansau, and A. Fuhrmeister, PHARMACOKINETICS OF GESTODENE IN 12 WOMEN WHO RECEIVED A SINGLE ORAL DOSE OF 0.075 MG GESTODENE AND, AFTER A WASH-OUT PHASE, THE SAME DOSE DURING ONE TREATMENT CYCLE. Contraception, 1992. 46(1): p. 29-40.

825. Kuhnz, W. and B. Lofberg, URINARY-EXCRETION OF 6-BETA-HYDROXYCORTISOL IN WOMEN DURING TREATMENT WITH DIFFERENT ORAL-CONTRACEPTIVE FORMULATIONS. Journal of Steroid Biochemistry and Molecular Biology, 1995. 55(1): p. 129-133.

826. Kuhnz, W., et al., INFLUENCE OF HIGH-DOSES OF VITAMIN-C ON THE BIOAVAILABILITY AND THE SERUM-PROTEIN BINDING OF LEVONORGESTREL IN WOMEN USING A COMBINATION ORAL-CONTRACEPTIVE. Contraception, 1995. 51(2): p. 111-116.

827. Kuhnz, W., B. Schutt, and R. Woloszczak, INFLUENCE OF CHANGES IN THE CONCENTRATION OF SEX HORMONE-BINDING GLOBULIN IN HUMAN SERUM ON THE PROTEIN-BINDING OF THE CONTRACEPTIVE STEROIDS LEVONORGESTREL, 3-KETO-DESOGESTREL AND GESTODENE. Journal of Steroid Biochemistry and Molecular Biology, 1994. 48(5-6): p. 573-580.

828. Kuhnz, W., T. Staks, and G. Jutting, PHARMACOKINETICS OF CYPROTERONE-ACETATE AND ETHINYLESTRADIOL IN 15 WOMEN WHO RECEIVED A COMBINATION ORAL-CONTRACEPTIVE DURING 3 TREATMENT CYCLES. Contraception, 1993. 48(6): p. 557-575.

829. Kuhnz, W., T. Staks, and G. Jutting, PHARMACOKINETICS OF LEVONORGESTREL AND ETHINYLESTRADIOL IN 14 WOMEN DURING 3 MONTHS OF TREATMENT WITH A TRI-STEP COMBINATION ORAL-CONTRACEPTIVE - SERUM-PROTEIN BINDING OF LEVONORGESTREL AND INFLUENCE OF TREATMENT ON FREE AND TOTAL TESTOSTERONE LEVELS IN THE SERUM. Contraception, 1994. 50(6): p. 563-579.

830. Kulaksiz, T.N., et al., Mouth Rinsing with Maltodextrin Solutions Fails to Improve Time Trial Endurance Cycling Performance in Recreational Athletes. Nutrients, 2016. 8(5).

831. Kulaksız, T.N., et al., Mouth Rinsing with Maltodextrin Solutions Fails to Improve Time Trial Endurance Cycling Performance in Recreational Athletes. Nutrients, 2016. 8(5).

832. Kumar, N., et al., Caffeine Ingestion With or Without Low-Dose Carbohydrate Improves Exercise Tolerance in Sedentary Adults. Frontiers in Nutrition, 2019. 6.

833. Kumar, N., et al., Carbohydrate ingestion but not mouth rinse maintains sustained attention when fasted. Physiology & behavior, 2016. 153: p. 33-9.

834. Kumar, V., et al., Pharmacokinetics of cefpodoxime in plasma and subcutaneous fluid following oral administration of cefpodoxime proxetil in male beagle dogs. Journal of Veterinary Pharmacology and Therapeutics, 2011. 34(2): p. 130-135.

835. Küng, M., S.W. Croley, and B.A. Phillips, Systemic cardiovascular and metabolic effects associated with the inhalation of an increased dose of albuterol. Influence of mouth rinsing and gargling. Chest, 1987. 91(3): p. 382-7.

836. Kunz, K., et al., Bioavailability of escin after administration of two oral formulations containing Aesculus extract. Arzneimittel-Forschung-Drug Research, 1998. 48(8): p. 822-825.

837. Kurita, H., et al., Usefulness of a commercial enzyme-linked immunosorbent assay kit for Candida mannan antigen for detecting Candida in oral rinse solutions. Oral surgery, oral medicine, oral pathology, oral radiology, and endodontics, 2009. 107(4): p. 531-4.

838. Kurzban, R., Does the Brain Consume Additional Glucose During Self-Control Tasks? Evolutionary Psychology, 2010. 8(2): p. 244-259.

839. Kusumoto, T., et al., Comprehensive epigenetic analysis using oral rinse samples: a pilot study. Journal of oral and maxillofacial surgery : official journal of the American Association of Oral and Maxillofacial Surgeons, 2012. 70(6): p. 1486-94.

840. Kuwayama, K., et al., Time-course measurements of caffeine and its metabolites extracted from fingertips after coffee intake: a preliminary study for the detection of drugs from fingerprints. Analytical and Bioanalytical Chemistry, 2013. 405(12): p. 3945-3952.

841. Lacasse, C., K.C. Gamble, and D.M. Boothe, Pharmacokinetics of a Single Dose of Intravenous and Oral Meloxicam in Red-tailed Hawks (Buteo jamaicensis) and Great Horned Owls (Bubo virginianus). Journal of Avian Medicine and Surgery, 2013. 27(3): p. 204-210.

842. LaCreta, F.P., et al., Interchangeability of 400-mg intravenous and oral gatifloxacin in healthy adults. Pharmacotherapy, 2000. 20(6): p. 59S-66S.

843. Laeijendecker, R., F. Heule, and T. van Joost, [Oral lichen ruber planus: clinical and immunological aspects]. Orale lichen ruber planus: klinische en immunologische aspecten., 1992. 136(44): p. 2167-71.

844. Laforgia, A., et al., Non-surgical periodontal management in scleroderma disease patients. Journal of biological regulators and homeostatic agents, 2016. 30(3): p. 847-851.

845. Lagerlöf, F. and C. Dawes, The effect of swallowing frequency on oral sugar clearance and pH changes by Streptococcus mitior in vivo after sucrose ingestion. J Dent Res, 1985. 64(10): p. 1229-32.

846. Lagerlöf, F., R. Dawes, and C. Dawes, The effects of different concentrations of sucrose, fructose, and glucose on pH changes by Streptococcus mitior in an artificial mouth. J Dent Res, 1985. 64(3): p. 405-10.

847. Lai, C., et al., EFFECT OF CALCIUM-ANTAGONISTS ON EXERCISE TESTS. Journal of Cardiovascular Pharmacology, 1992. 20: p. S55-S64.

848. Lai, C.M., et al., DETERMINATION OF DUP-128, AN ACAT INHIBITOR AND ITS SULFOXIDE AND SULFONE METABOLITES IN HUMAN PLASMA BY LIQUID-CHROMATOGRAPHY. Journal of Pharmaceutical and Biomedical Analysis, 1994. 12(9): p. 1163-1172.

849. Lal, K., et al., Assessment of antimicrobial treatment of denture stomatitis using an in vivo replica model system: therapeutic efficacy of an oral rinse. J Prosthet Dent, 1992. 67(1): p. 72-7.

850. Lal, R., et al., Pharmacokinetics and Tolerability of Single Escalating Doses of Gabapentin Enacarbil: A Randomized-Sequence, Double-Blind, Placebo-Controlled Crossover Study in Healthy Volunteers. Clinical Therapeutics, 2009. 31(8): p. 1776-1786.

851. Lal, R., et al., The effect of food with varying fat content on the clinical pharmacokinetics of gabapentin after oral administration of gabapentin enacarbil. International Journal of Clinical Pharmacology and Therapeutics, 2010. 48(2): p. 120-128.

852. Lamarre-Cliche, M., et al., Pharmacokinetic and Pharmacodynamic Effects of Midodrine on Blood Pressure, the Autonomic Nervous System, and Plasma Natriuretic Peptides: A Prospective, Randomized, Single-Blind, Two-Period, Crossover, Placebo-Controlled Study. Clinical Therapeutics, 2008. 30(9): p. 1629-1638.

853. Lane, S.C., et al., Effect of a carbohydrate mouth rinse on simulated cycling time-trial performance commenced in a fed or fasted state. Applied physiology, nutrition, and metabolism = Physiologie appliquee, nutrition et metabolisme, 2013. 38(2): p. 134-9.

854. Lane, S.C., et al., Single and combined effects of beetroot juice and caffeine supplementation on cycling time trial performance. Applied Physiology Nutrition and Metabolism, 2014. 39(9): p. 1050-1057.

855. Lange, D., et al., The effect of coadministration of a cola beverage on the bioavailability of itraconazole in patients with acquired immunodeficiency syndrome. Current Therapeutic Research-Clinical and Experimental, 1997. 58(3): p. 202-212.

856. Lange, F. and F. Eggert, Sweet delusion. Glucose drinks fail to counteract ego depletion. Appetite, 2014. 75: p. 54-63.

857. Langenbuch, T., et al., A phase II study of intensive-dose epirubicin/verapamil as induction therapy followed by intensive-dose ifosfamide for advanced breast cancer. Cancer chemotherapy and pharmacology, 1990. 26 Suppl: p. S93-6.

858. Langevin, S.M., et al., MicroRNA-137 promoter methylation in oral rinses from patients with squamous cell carcinoma of the head and neck is associated with gender and body mass index. Carcinogenesis, 2010. 31(5): p. 864-70.

859. Langford, R., et al., The WHO Health Promoting School framework for improving the health and well-being of students and their academic achievement. Cochrane Database of Systematic Reviews, 2014(4).

860. Lanigan, R.S., Final report on the safety assessment of PEG-7,-30,-40,-78, and-80 Glyceryl Cocoate. International Journal of Toxicology, 1999. 18: p. 33-42.

861. Lapis, T.J., M.H. Penner, and J. Lim, Evidence that Humans Can Taste Glucose Polymers. Chemical Senses, 2014. 39(9): p. 737-747.

862. Laster, L.L. and R.R. Lobene, New perspectives on Sanguinaria clinicals: individual toothpaste and oral rinse testing. Journal (Canadian Dental Association), 1990. 56(7 Suppl): p. 19-30.

863. Laws, H.L., R.H. Clements, and C.M. Swillie, A randomized, prospective comparison of the Nissen fundoplication versus the Toupet fundoplication for gastroesophageal reflux disease. Annals of surgery, 1997. 225(6): p. 647-654.

864. Lawton, H.M., J.M. Battagel, and B. Kotecha, A comparison of the Twin Block and Herbst mandibular advancement splints in the treatment of patients with obstructive sleep apnoea: a prospective study. European Journal of Orthodontics, 2005. 27(1): p. 82-90.

865. LeBlanc-Duchin, D. and H.K. Taukulis, Behavioral reactivity to a noradrenergic challenge after chronic oral methylphenidate (Ritalin((R))) in rats. Pharmacology Biochemistry and Behavior, 2004. 79(4): p. 641-649.

866. Lee, H.J., et al., Bioequivalence assessment of ambroxol tablet after a single oral dose administration to healthy male volunteers. Pharmacological Research, 2004. 49(1): p. 93-98.

867. Lee, H.J., et al., Bioequivalence of a prednisolone tablet administered as a single oral dose in healthy male volunteers. International Journal of Clinical Pharmacology and Therapeutics, 2004. 42(9): p. 519-525.

868. Lee, J.S., et al., Phase I clinical trial of the combination of eribulin and everolimus in patients with metastatic triple-negative breast cancer. Breast cancer research : BCR, 2019. 21(1): p. 119.

869. Lee, J.S., et al., Phase I clinical trial of the combination of eribulin and everolimus in patients with metastatic triple-negative breast cancer. Breast Cancer Research, 2019. 21(1).

870. Lee, Y., et al., Role of Prostaglandin Pathway and Alendronate-Based Carriers To Enhance Statin-Induced Bone. Molecular Pharmaceutics, 2011. 8(4): p. 1035-1042.

871. Lee, Y.J., S.J. Chung, and C.K. Shim, Decreased oral availability of cyclosporin A at second administration in humans. British Journal of Clinical Pharmacology, 1997. 44(4): p. 343-345.

872. Lehmann, A.S., et al., Collection of human genomic DNA from neonates: a comparison between umbilical cord blood and buccal swabs. American Journal of Obstetrics and Gynecology, 2011. 204(4).

873. Lemay, A., et al., Flaxseed dietary supplement versus hormone replacement therapy in hypercholesterolemic menopausal women. Obstetrics and Gynecology, 2002. 100(3): p. 495-504.

874. Lennon, A.M., et al., Effect of a casein/calcium phosphate-containing tooth cream and fluoride on enamel erosion in vitro. Caries Res, 2006. 40(2): p. 154-7.

875. Leppilahti, J.M., et al., Diagnosis of Newly Delivered Mothers for Periodontitis with a Novel Oral-Rinse aMMP-8 Point-of-Care Test in a Rural Malawian Population. Diagnostics, 2018. 8(3).

876. Lettieri, J., et al., Effect of food on the pharmacokinetics of a single oral dose of moxifloxacin 400mg in healthy male volunteers. Clinical Pharmacokinetics, 2001. 40: p. 19-25.

877. Levrini, L., et al., pH of tooth surface in healthy adolescents at rest and after a glucose rinse: effect of 72 hours of plaque accumulation. Eur J Paediatr Dent, 2012. 13(4): p. 293-6.

878. Li, L., et al., Determination of thiorphan, a racecadotril metabolite, in human plasma by LC-MS/MS and its application to a bioequivalence study in Chinese subjects. International Journal of Clinical Pharmacology and Therapeutics, 2020. 58(7): p. 408-414.

879. Li, S., Y. Wu, and Y. Cao, Comment on: "Effects of Carbohydrate Mouth Rinse on Cycling Time Trial Performance: A Systematic Review and Meta-Analysis". Sports medicine (Auckland, N.Z.), 2019. 49(5): p. 819-821.

880. Li, Y.J., et al., Pharmacokinetics and Bioequivalence of Rasagiline Tablets in Chinese Healthy Subjects Under Fasting and Fed Conditions: An Open, Randomized, Single-Dose, Double-Cycle, Two-Sequence, Crossover Trial. Frontiers in Pharmacology, 2020. 11.

881. Lim, Y., et al., The Performance of an Oral Microbiome Biomarker Panel in Predicting Oral Cavity and Oropharyngeal Cancers. Frontiers in cellular and infection microbiology, 2018. 8: p. 267.

882. Lim, Z.M., Q.T. Chie, and L.K. Teh, Influence of dopamine receptor gene on eating behaviour and obesity in Malaysia. Meta Gene, 2020. 25.

883. Limaye, S.A., et al., Phase 1b, multicenter, single blinded, placebo-controlled, sequential dose escalation study to assess the safety and tolerability of topically applied AG013 in subjects with locally advanced head and neck cancer receiving induction chemotherapy. Cancer, 2013. 119(24): p. 4268-76.

884. Limopasmanee, W., et al., Effects of the Chinese Herbal Formulation (Liu Wei Di Huang Wan) on the Pharmacokinetics of Isoflavones in Postmenopausal Women. Biomed Research International, 2015. 2015.

885. Lin, J.X., et al., A comparison of dioctahedral smectite and iodine glycerin cream with topical mouth rinse in treatment of chemotherapy induced oral mucositis: a pilot study. Eur J Oncol Nurs, 2015. 19(2): p. 136-41.

886. Lin, T.Y., M.H. Yang, and F.Y. Chang, A Randomized, Phase I, 3-way Crossover Study to Examine the Effects of Food on the Pharmacokinetics of Single Doses of 400 mg Posaconazole Oral Suspension in Healthy Male Taiwanese Subjects. Therapeutic Drug Monitoring, 2013. 35(2): p. 223-227.

887. Lin, Y.J., Buccal absorption of triclosan following topical mouthrinse application. Am J Dent, 2000. 13(4): p. 215-7.

888. Lindquist, B., et al., Influence of five neutralizing products on intra-oral pH after rinsing with simulated gastric acid. Eur J Oral Sci, 2011. 119(4): p. 301-4.

889. Lindsley, K., et al., Interventions for chronic blepharitis. Cochrane Database of Systematic Reviews, 2012(5).

890. Lingström, P., et al., The pH of dental plaque in its relation to early enamel caries and dental plaque flora in humans. J Dent Res, 2000. 79(2): p. 770-7.

891. Lipworth, B.J. and D.J. Clark, Early lung absorption profile of non-CFC salbutamol via small and large volume plastic spacer devices. British journal of clinical pharmacology, 1998. 46(1): p. 45-8.

892. Lipworth, B.J., et al., Effects of adding a leukotriene antagonist or a long-acting beta(2)-agonist in asthmatic patients with the glycine-16 beta(2)-adrenoceptor genotype. American Journal of Medicine, 2000. 109(2): p. 114-121.

893. Liu, R., et al., Effects of Sodium Bicarbonate and Ammonium Chloride Pre-treatments on PEPT2 (SLC15A2) Mediated Renal Clearance of Cephalexin in Healthy Subjects. Drug Metabolism and Pharmacokinetics, 2011. 26(1): p. 87-93.

894. Liu, Y.G., et al., Cilostazol and dipyridamole synergistically inhibit human platelet aggregation. Journal of Cardiovascular Pharmacology, 2004. 44(2): p. 266-273.

895. Liu, Z.W., T.N. Lin, and G.Z. He, [Research of compound cyclosporin A mouthwash in the treatment of oral lichen planus]. Hunan yi ke da xue xue bao = Hunan yike daxue xuebao = Bulletin of Hunan Medical University, 2000. 25(2): p. 183-4.

896. Lodi, G., et al., Interventions for treating oral lichen planus: corticosteroid therapies. Cochrane Database of Systematic Reviews, 2020(2).

897. Lohitnavy, M., et al., Average bioequivalence of clarithromycin immediate released tablet formulations in healthy male volunteers. Drug Development and Industrial Pharmacy, 2003. 29(6): p. 653-659.

898. Loimaranta, V., et al., Effects of bovine immune and non-immune whey preparations on the composition and pH response of human dental plaque. Eur J Oral Sci, 1999. 107(4): p. 244-50.

899. Loo, W.T.Y., et al., Rhodiola algida improves chemotherapy-induced oral mucositis in breast cancer patients. Expert opinion on investigational drugs, 2010. 19 Suppl 1: p. S91-100.

900. Lopez-Gil, J.A., Fluconazole-cyclosporine interaction: a dose-dependent effect? The Annals of pharmacotherapy, 1993. 27(4): p. 427-30.

901. Lore, B., et al., Oral lichen planus: therapy and phenotype. Giornale italiano di dermatologia e venereologia : organo ufficiale, Societa italiana di dermatologia e sifilografia, 2018. 153(4): p. 459-463.

902. Lorenzen, J., et al., MR-imaging of the breast at 0.5 tesla: Menstrual-cycle dependency of parenchymal contrast enhancement in healthy volunteers with oral contraceptive use? Rofo-Fortschritte Auf Dem Gebiet Der Rontgenstrahlen Und Der Bildgebenden Verfahren, 2003. 175(4): p. 502-506.

903. Lu, L.J.W., et al., Increased urinary excretion of 2-hydroxyestrone but not 16 alpha-hydroxyestrone in premenopausal women during a soya diet containing isoflavones. Cancer Research, 2000. 60(5): p. 1299-1305.

904. Lucas, B.D., et al., Effect of oral H-2-receptor antagonists on left ventricular systolic function and exercise capacity in patients with chronic stable heart failure. Pharmacotherapy, 1998. 18(4): p. 824-830.

905. Lucky, M.H. and S. Baig, Isolation of DNA from oral rinse in HPV positive patients. Journal of the College of Physicians and Surgeons--Pakistan : JCPSP, 2013. 23(7): p. 455-8.

906. Luden, N.D., et al., Carbohydrate Mouth Rinsing Enhances High Intensity Time Trial Performance Following Prolonged Cycling. Nutrients, 2016. 8(9).

907. Luebke, T.E. and J.A. Driskell, A group of Midwestern university students needs to improve their oral hygiene and sugar/pop consumption habits. Nutr Res, 2010. 30(1): p. 27-31.

908. Luke, G.A., et al., Human salivary sugar clearance after sugar rinses and intake of foodstuffs. Caries Res, 1999. 33(2): p. 123-9.

909. Luke, G.A., et al., Human salivary sugar clearance after sugar rinses and intake of foodstuffs. Caries Research, 1999. 33(2): p. 123-129.

910. Lundy, R.F., Jr. and R.J. Contreras, Taste prestimulation increases the chorda tympani nerve response to menthol. Physiol Behav, 1993. 54(1): p. 65-70.

911. Luoma, H., A.R. Luoma, and L. Seppä, Exchange of fluoride between bovine enamel and the surface-related cells of the oral bacterium Streptococcus mutans. Arch Oral Biol, 1984. 29(5): p. 343-8.

912. Luostarinen, V., K.K. Makinen, and P.L. Makinen, Effects on oral health of mouthrinses containing xylitol, sodium cyclamate and sucrose sweeteners in the absence of oral hygiene. V. Response of hamster cheek pouch microcirculation to dental plaque. Proceedings of the Finnish Dental Society. Suomen Hammaslaakariseuran toimituksia, 1984. 80(1): p. 35-9.

913. Lussi, A., et al., Effect of amine/sodium fluoride rinsing on toothbrush abrasion of softened enamel in situ. Caries research, 2004. 38(6): p. 567-71.

914. Luvizuto, E.R., et al., Functional aesthetic treatment of patient with phenytoin-induced gingival overgrowth. The Journal of craniofacial surgery, 2012. 23(3): p. e174-6.

915. Machado, A., et al., Anti-erosive effect of rinsing before or after toothbrushing with a Fluoride/Stannous Ions solution: an in situ investigation: Application order of Fluoride/Tin products for erosive tooth wear. Journal of dentistry, 2020. 101: p. 103450.

916. Madeswaran, S. and S. Jayachandran, Sodium bicarbonate: A review and its uses in dentistry. Indian J Dent Res, 2018. 29(5): p. 672-677.

917. Maesen, F.P.V., et al., EFFECT OF ZATEBRADINE, A NOVEL SINUS NODE INHIBITOR, ON PULMONARY-FUNCTION COMPARED TO PLACEBO. Pulmonary Pharmacology & Therapeutics, 1994. 7(6): p. 349-355.

918. Mahajan, T., An In Vivo Study to Evaluate the Efficacy of Aloe-Vera and 0.2% Chlorhexidine Digluconate as Preprocedural Mouth Rinses for Disinfecting Alginate Impression. Journal of Evolution of Medical and Dental Sciences-Jemds, 2020. 9(35): p. 2521-2525.

919. Maharaj, B., Y. Coovadia, and A.C. Vayej, A comparative study of amoxicillin, clindamycin and chlorhexidine in the prevention of post-extraction bacteraemia. Cardiovasc J Afr, 2012. 23(9): p. 491-4.

920. Mahatthanatrakul, W., et al., Bioequivalence study of a generic quetiapine in healthy male volunteers. International Journal of Clinical Pharmacology and Therapeutics, 2008. 46(9): p. 489-496.

921. Mair, L.H. and P. Padipatvuthikul, FATIGUE FRACTURE OF DENTAL RESIN BONDED CERAMIC DISKS, in Fractography of Glasses and Ceramics V, J.R. Varner, G.D. Quinn, and M. Wightman, Editors. 2007. p. 293-301.

922. Maiwald, H.J. and S. Fröhlich, A modified plaque pH telemetry method. J Clin Dent, 1992. 3(3): p. 79-82.

923. Makinen, K.K., et al., Effects on oral health of mouthrinses containing xylitol, sodium cyclamate and sucrose sweeteners in the absence of oral hygiene. II. Relative composition of free amino acids in human crevicular fluid. Proceedings of the Finnish Dental Society. Suomen Hammaslaakariseuran toimituksia, 1984. 80(1): p. 13-9.

924. Mäkinen, K.K., et al., Effect of xylitol-, sucrose-, and water-rinses on the composition of human palatine gland secretions. Scand J Dent Res, 1985. 93(3): p. 253-61.

925. Makkonen, T.A., et al., Sucralfate mouth washing in the prevention of radiation-induced mucositis: a placebo-controlled double-blind randomized study. Int J Radiat Oncol Biol Phys, 1994. 30(1): p. 177-82.

926. Mallery, S.R., et al., Effects of human oral mucosal tissue, saliva, and oral microflora on intraoral metabolism and bioactivation of black raspberry anthocyanins. Cancer Prev Res (Phila), 2011. 4(8): p. 1209-21.

927. Maloney, J.M., M.D. Chapman, and S.H. Sicherer, Peanut allergen exposure through saliva: assessment and interventions to reduce exposure. J Allergy Clin Immunol, 2006. 118(3): p. 719-24.

928. Manfredi, M., et al., The isolation, identification and molecular analysis of Candida spp. isolated from the oral cavities of patients with diabetes mellitus. Oral Microbiol Immunol, 2002. 17(3): p. 181-5.

929. Mann, E.A., et al., Effect of topical antibiotic therapy on recovery after tonsillectomy in adults. Otolaryngol Head Neck Surg, 1999. 121(3): p. 277-82.

930. Manorot, M., et al., Pharmacokinetics and bioequivalence testing of generic fluconazole preparations in healthy Thai volunteers. International Journal of Clinical Pharmacology and Therapeutics, 2000. 38(7): p. 355-359.

931. Mansouri, P., et al., Oral submucosal fibrosis in Iran: a case review. International journal of dermatology, 2010. 49(12): p. 1424-8.

932. Manton, D.J., et al., Effect of casein phosphopeptide-amorphous calcium phosphate added to acidic beverages on enamel erosion in vitro. Aust Dent J, 2010. 55(3): p. 275-9.

933. Manton, D.J., et al., Remineralization of enamel subsurface lesions in situ by the use of three commercially available sugar-free gums. International Journal of Paediatric Dentistry, 2008. 18(4): p. 284-290.

934. Margolis, H.C. and E.C. Moreno, Composition of pooled plaque fluid from caries-free and caries-positive individuals following sucrose exposure. J Dent Res, 1992. 71(11): p. 1776-84.

935. Margolis, H.C., et al., Cariogenic potential of pooled plaque fluid from exposed root surfaces in humans. Arch Oral Biol, 1993. 38(2): p. 131-8.

936. Marinho, A.H., et al., Caffeine mouth rinse has no effects on anaerobic energy yield during a Wingate Test. The Journal of sports medicine and physical fitness, 2020. 60(1): p. 69-74.

937. Martin, N.E., et al., Pharmacoscintigraphic assessment of the regional drug absorption of the dual angiotensin-converting enzyme/neutral endopeptidase inhibitor, M100240, in healthy volunteers. Journal of Clinical Pharmacology, 2003. 43(5): p. 529-538.

938. Maruyama, S., et al., Glucose retention on the surfaces of primary teeth in 3- and 4-yr-old children. Arch Oral Biol, 1995. 40(9): p. 783-7.

939. Marvin, U.B., Oral histories in meteoritics and planetary science: VIII. Friedrich Begemann. Meteoritics & Planetary Science, 2002. 37(12): p. B69-B77.

940. Mas, M., et al., Cardiovascular and neuroendocrine effects and pharmacokinetics of 3,4-methylenedioxymethamphetamine in humans. Journal of Pharmacology and Experimental Therapeutics, 1999. 290(1): p. 136-145.

941. Masa, K., et al., Effect of clarithromycin and other macrolides on the sulfoxidation and 5-hydroxylation of lansoprazole in dogs. Biological & Pharmaceutical Bulletin, 1999. 22(5): p. 504-509.

942. Masri, M.A., et al., The role of generics in transplantation: TM-MMF versus Cellcept in healthy volunteers. Transplantation Proceedings, 2004. 36(1): p. 84-85.

943. Masri, M.A., et al., Bioavailability of a new generic formulation of mycophenolate mofetil MMF 500 versus CellCept in healthy adult volunteers. Transplantation Proceedings, 2007. 39(4): p. 1233-1236.

944. Mata, F., et al., Carbohydrate Availability and Physical Performance: Physiological Overview and Practical Recommendations. Nutrients, 2019. 11(5).

945. Matar, K.M., A.I. Awad, and S.B. Elamin, Pharmacokinetics of Artesunate Alone and in Combination with Sulfadoxine/Pyrimethamine in Healthy Sudanese Volunteers. American Journal of Tropical Medicine and Hygiene, 2014. 90(6): p. 1087-1093.

946. Matsumoto, H., T. Hasegawa, and K. Ishihara, [Concerns about adverse effects of inhaled corticosteroids]. Nihon Rinsho, 1996. 54(11): p. 2998-3003.

947. Matsuoka, H., et al., [Clinical availability of the herbal medicine, SYOUSAIKOTOU, as a gargling agent for prevention and treatment of chemotherapy-induced stomatitis]. Gan to kagaku ryoho. Cancer & chemotherapy, 2004. 31(12): p. 2017-20.

948. Mauland, E.K., H.R. Preus, and A.M. Aass, Comparison of commercially available 0.2% chlorhexidine mouthwash with and without anti-discoloration system: A blinded, crossover clinical trial. Journal of clinical periodontology, 2020. 47(12): p. 1522-1527.

949. Mawardi, H., et al., Osteonecrosis of the jaw associated with ziv-aflibercept. Journal of Gastrointestinal Oncology, 2016. 7(6): p. E81-E87.

950. Maya, M.T., et al., Comparative bioavailability of two immediate release tablets of cisapride in healthy volunteers. European Journal of Drug Metabolism and Pharmacokinetics, 1998. 23(3): p. 377-381.

951. Maya, M.T., et al., Bioequivalence evaluation of three different oral formulations of ciprofloxacin in healthy volunteers. European Journal of Drug Metabolism and Pharmacokinetics, 2003. 28(2): p. 129-136.

952. Maya, M.T., et al., Comparative Bioavailability of two immediate release tablets of enalapril/hydrochlorothiazide in healthy volunteers. European Journal of Drug Metabolism and Pharmacokinetics, 2002. 27(2): p. 91-99.

953. Mazzi-Chaves, J.F., et al., Effect of a chitosan final rinse on the bond strength of root canal fillings. Gen Dent, 2019. 67(5): p. 54-57.

954. Mazzoni, A., et al., Adhesion to chondroitinase ABC treated dentin. J Biomed Mater Res B Appl Biomater, 2008. 86(1): p. 228-36.

955. McCord, J.L., J.M. Beasley, and J.R. Halliwill, H-2-receptor-mediated vasodilation contributes to postexercise hypotension. Journal of Applied Physiology, 2006. 100(1): p. 67-75.

956. McCoy, C.P., et al., Determination of the salivary retention of hexetidine in-vivo by high-performance liquid chromatography. The Journal of pharmacy and pharmacology, 2000. 52(11): p. 1355-9.

957. McCoy, R.A., et al., PHARMACODYNAMICS OF RACEMIC AND S(-)-ATENOLOL IN HUMANS. Journal of Clinical Pharmacology, 1994. 34(8): p. 816-822.

958. McDonagh, S.T.J., et al., The Effects of Chronic Nitrate Supplementation and the Use of Strong and Weak Antibacterial Agents on Plasma Nitrite Concentration and Exercise Blood Pressure. International journal of sports medicine, 2015. 36(14): p. 1177-85.

959. McGrath, L.T., et al., Production of 8-epi prostaglandin F-2 alpha in human platelets during administration of organic nitrates. Journal of the American College of Cardiology, 2002. 40(4): p. 820-825.

960. McNeal, C.J., et al., Safety of dietary supplementation with arginine in adult humans. Amino Acids, 2018. 50(9): p. 1215-1229.

961. Medvedovici, A., et al., A non-extracting procedure for the determination of meloxicam in plasma samples by HPLC-diode array detection. Arzneimittelforschung-Drug Research, 2005. 55(6): p. 326-331.

962. Meeusen, R., Exercise, Nutrition and the Brain. Sports Medicine, 2014. 44: p. 47-56.

963. Meeusen, R. and L. Decroix, Nutritional Supplements and the Brain. International Journal of Sport Nutrition and Exercise Metabolism, 2018. 28(2): p. 200-211.

964. Meijers, J.C.M., et al., Increased fibrinolytic activity during use of oral contraceptives is counteracted by an enhanced factor XI-independent down regulation of fibrinolysis - A randomized cross-over study of two low-dose oral contraceptives. Thrombosis and Haemostasis, 2000. 84(1): p. 9-14.

965. Mendes, G.D., et al., Cyclosporine bioequivalence study: quantification using fluorescence polarization immunoassay (FPIA) and radioimmunoassay (RIA). International Journal of Clinical Pharmacology and Therapeutics, 2004. 42(2): p. 125-132.

966. Mendieta, C., et al., Comparison of 2 chlorhexidine mouthwashes on plaque regrowth in vivo and dietary staining in vitro. Journal of clinical periodontology, 1994. 21(4): p. 296-300.

967. Mendieta, C., et al., COMPARISON OF 2 CHLORHEXIDINE MOUTHWASHES ON PLAQUE REGROWTH IN-VIVO AND DIETARY STAINING IN-VITRO. Journal of Clinical Periodontology, 1994. 21(4): p. 296-300.

968. Meningaud, J.P., et al., [Halitosis in 1999]. Rev Stomatol Chir Maxillofac, 1999. 100(5): p. 240-4.

969. Menon, S., et al., A randomized, crossover study to determine bioequivalence of two brands of dexibuprofen 400 mg tablets in healthy Asian adult male subjects of Indian origin. International Journal of Clinical Pharmacology and Therapeutics, 2008. 46(1): p. 48-54.

970. Merchant, A.T., et al., Oral care practices and A1c among youth with type 1 and type 2 diabetes. J Periodontol, 2012. 83(7): p. 856-63.

971. Mermelstein, F., et al., Single-Dose and Multiple-Dose Pharmacokinetics and Dose Proportionality of Intravenous and Intramuscular HP beta CD-Diclofenac (Dyloject) Compared with Other Diclofenac Formulations. Pharmacotherapy, 2013. 33(10): p. 1012-1021.

972. Messer, L.B. and H. Calache, Oral health attitudes and behaviours of final-year dental students. Eur J Dent Educ, 2012. 16(3): p. 144-55.

973. Meurman, J.H., et al., Effect of antiseptic mouthwashes on some clinical and microbiological findings in the mouths of lymphoma patients receiving cytostatic drugs. J Clin Periodontol, 1991. 18(8): p. 587-91.

974. Meyer, N.L. and J. Stevens, SPORTS DIETETICS USA RESEARCH DIGEST. (Abstract). Scan's Pulse, 2005. 24(3): p. 24-26.

975. Mickleborough, T., Carbohydrate Mouth Rinses May Improve Performance. Triathlete, 2009(301): p. 145-146.

976. Middeldorp, S., et al., Effects on coagulation of levonorgestrel- and desogestrel-containing low dose oral contraceptives: a cross-over study. Thrombosis and Haemostasis, 2000. 84(1): p. 4-8.

977. Miller, H.C., C. Bourrasseau, and J. Blampain, Can you enhance executive control without glucose? The effects of fructose on problem solving. J Psychopharmacol, 2013. 27(7): p. 645-50.

978. Min, D.I., et al., Effect of grapefruit juice on the pharmacokinetics and pharmacodynamics of quinidine in healthy volunteers. Journal of Clinical Pharmacology, 1996. 36(5): p. 469-476.

979. Min, D.I., et al., A urine metabolic ratio of dextromethorphan and 3-methoxymorphinan as a probe for CYP3A activity and prediction of cyclosporine clearance in healthy volunteers. Pharmacotherapy, 1999. 19(6): p. 753-759.

980. Minocha, M., et al., Pharmacokinetics of the B-Cell Lymphoma 2 (Bcl-2) Inhibitor Venetoclax in Female Subjects with Systemic Lupus Erythematosus. Clinical Pharmacokinetics, 2018. 57(9): p. 1185-1198.

981. Mirlohi, S., et al., Analysis of salivary fluid and chemosensory functions in patients treated for primary malignant brain tumors. Clinical oral investigations, 2015. 19(1): p. 127-37.

982. Mitsui, T. and T. Kondo, Effects of mouth cleansing on the levels of exhaled nitrous oxide in young and older adults. The Science of the total environment, 1998. 224(1-3): p. 177-80.

983. Miura, A., et al., The effect of oral creatine supplementation on the curvature constant parameter of the power-duration curve for cycle ergometry in humans. Japanese Journal of Physiology, 1999. 49(2): p. 169-174.

984. Moeremans, I., et al., Pharmacokinetics and absolute oral bioavailability of meloxicam in guinea pigs (Cavia porcellus). Veterinary Anaesthesia and Analgesia, 2019. 46(4): p. 548-555.

985. Moghadamnia, Y., et al., New formulation of ibuprofen on absorption-rate: A comparative bioavailability study in healthy volunteers. Caspian Journal of Internal Medicine, 2019. 10(2): p. 150-155.

986. Mohamed, N.S., et al., Impact of three different mouthwashes on the incidence of gingival overgrowth induced by cyclosporine-A: a randomized controlled experimental animal study. Oral surgery, oral medicine, oral pathology and oral radiology, 2015. 120(3): p. 346-56.

987. Moi, G.P., L.M.A. Tenuta, and J.A. Cury, Anticaries potential of a fluoride mouthrinse evaluated in vitro by validated protocols. Brazilian dental journal, 2008. 19(2): p. 91-6.

988. Molden, D.C., et al., Motivational versus metabolic effects of carbohydrates on self-control. Psychol Sci, 2012. 23(10): p. 1137-44.

989. Molina, P.L.G., et al., Bioequivalence study of three ascorbic acid tablet formulations in healthy male volunteers. Clinical Research and Regulatory Affairs, 1998. 15(2): p. 115-130.

990. Montoya-Botero, P., et al., The effect of type of oral contraceptive pill and duration of use on fresh and cumulative live birth rates in IVF/ICSI cycles. Human Reproduction, 2020. 35(4): p. 826-836.

991. Moore, J., et al., Laboratory screening evaluation of the safety of low pH oral care rinse products to dental enamel. American journal of dentistry, 2020. 33(3): p. 161-164.

992. Moore, J., et al., Laboratory screening evaluation of the safety of low pH oral care rinse products to dental enamel. American Journal of Dentistry, 2020. 33(3): p. 161-164.

993. Moore, L.E., F.D. Boudinot, and C.K. Chu, Preclinical pharmacokinetics of beta-L-dioxolane-cytidine, a novel anticancer agent, in rats. Cancer Chemotherapy and Pharmacology, 1997. 39(6): p. 532-536.

994. Morales-Bozo, I., et al., Evaluation of the efficacy of two mouthrinses formulated for the relief of xerostomia of diverse origin in adult subjects. Gerodontology, 2012. 29(2): p. e1103-12.

995. Moran, J., et al., A clinical study to assess the ability of a powered toothbrush to remove chlorhexidine/tea dental stain. Journal of clinical periodontology, 2004. 31(2): p. 95-8.

996. Morera, M., et al., Bioequivalence of two oral ciprofloxacin formulations. Clinical Drug Investigation, 2001. 21(2): p. 137-145.

997. Morris, N.B., et al., Evidence that transient changes in sudomotor output with cold and warm fluid ingestion are independently modulated by abdominal, but not oral thermoreceptors. Journal of applied physiology (Bethesda, Md. : 1985), 2014. 116(8): p. 1088-95.

998. Mortazavi, S., et al., Persica chewing gum effects on saliva fluoride concentration and flow rate: A triple-blind randomized clinical trial. Contemporary Clinical Dentistry, 2019. 10(1): p. 117-122.

999. Moton, A., et al., Effects of oral posaconazole on the pharmacokinetics of sirolimus. Current Medical Research and Opinion, 2009. 25(3): p. 701-707.

1000. Mouhyi, J., et al., Mouthwash solutions containing microencapsulated natural extracts: Clinical results on dental plaque and gingivitis. Revue De Stomatologie De Chirurgie Maxillo-Faciale Et De Chirurgie Orale, 2010. 111(3): p. 144-147.

1001. Moyad, M.A., et al., Vitamin C Metabolites, Independent of Smoking Status, Significantly Enhance Leukocyte, but not Plasma Ascorbate Concentrations. Advances in Therapy, 2008. 25(10): p. 995-1009.

1002. Moynihan, P.J., et al., Acid production from lactulose by dental plaque bacteria. Lett Appl Microbiol, 1998. 27(3): p. 173-7.

1003. Moynihan, P.J., et al., Effect of glucose polymers in water, milk and a milk substitute on plaque pH in vitro. Int J Paediatr Dent, 1996. 6(1): p. 19-24.

1004. Muana, H.L., et al., Effect of the Dentin Chelating Agents Phytic Acid and EDTA on Degree of Conversion, Microhardness, and Bond Strength of Chemical-curing Self-adhesive Cements. J Adhes Dent, 2019. 21(4): p. 299-306.

1005. Muddathir, A.M., E.A.M. Mohieldin, and T. Mitsunaga, In vitro activities of Acacia nilotica (L.) Delile bark fractions against Oral Bacteria, Glucosyltransferase and as antioxidant. Bmc Complementary Medicine and Therapies, 2020. 20(1).

1006. Mueller, M.E., et al., Gabapentin for relief of upper motor neuron symptoms in multiple sclerosis. Archives of Physical Medicine and Rehabilitation, 1997. 78(5): p. 521-524.

1007. Muguruma, T., et al., Effects of sodium fluoride mouth rinses on the torsional properties of miniscrew implants. American journal of orthodontics and dentofacial orthopedics : official publication of the American Association of Orthodontists, its constituent societies, and the American Board of Orthodontics, 2011. 139(5): p. 588-93.

1008. Muguruma, T., et al., Effects of sodium fluoride mouth rinses on the torsional properties of miniscrew implants. American Journal of Orthodontics and Dentofacial Orthopedics, 2011. 139(5): p. 588-593.

1009. Muhamad, A.S., N.F.R. Mohd Puad, and G. Kuan, Effects of Carbohydrate Mouth Rinsing on Salivary Lysozyme, Mood States and Running Performance Among Recreational Runners. Malaysian Journal of Medical Sciences, 2020. 27(1): p. 87-96.

1010. Muhamad, A.S., N. Puad, and G. Kuan, Effects of Carbohydrate Mouth Rinsing on Salivary Lysozyme, Mood States and Running Performance Among Recreational Runners. Malays J Med Sci, 2020. 27(1): p. 87-96.

1011. Muhamed, A.M.C., et al., Mouth rinsing improves cycling endurance performance during Ramadan fasting in a hot humid environment. Applied Physiology Nutrition and Metabolism, 2014. 39(4): p. 458-464.

1012. Mukherjee, P.K., et al., Metabolomic analysis identifies differentially produced oral metabolites, including the oncometabolite 2-hydroxyglutarate, in patients with head and neck squamous cell carcinoma. Bba Clinical, 2017. 7: p. 8-15.

1013. Mullins, K.B., et al., Effects of carprofen, meloxicam and deracoxib on platelet function in dogs. Veterinary Anaesthesia and Analgesia, 2012. 39(2): p. 206-217.

1014. Mulshine, J.L., et al., Randomized, double-blind, placebo-controlled phase IIb trial of the cyclooxygenase inhibitor ketorolac as an oral rinse in oropharyngeal leukoplakia. Clinical cancer research : an official journal of the American Association for Cancer Research, 2004. 10(5): p. 1565-73.

1015. Muniyappa, R., et al., Oral glucosamine for 6 weeks at standard doses does not cause or worsen insulin resistance or endothelial dysfunction in lean or obese subjects. Diabetes, 2006. 55(11): p. 3142-3150.

1016. Murphy, R.M., et al., Effects of creatine supplementation on housekeeping genes in human skeletal muscle using real-time RT-PCR. Physiological Genomics, 2003. 12(2): p. 163-174.

1017. Murray, B. and C. Rosenbloom, Fundamentals of glycogen metabolism for coaches and athletes. Nutrition Reviews, 2018. 76(4): p. 243-259.

1018. Murray, K.O., et al., Carbohydrate Mouth Rinse Improves Cycling Time-Trial Performance without Altering Plasma Insulin Concentration. Journal of Sports Science & Medicine, 2018. 17(1): p. 145-152.

1019. Myerson, S., et al., Human angiotensin I-converting enzyme gene and endurance performance. Journal of applied physiology (Bethesda, Md. : 1985), 1999. 87(4): p. 1313-6.

1020. Nagy, C.F., et al., Concurrent administration of donepezil HCl and sertraline HCl in healthy volunteers: assessment of pharmacokinetic changes and safety following single and multiple oral doses. British Journal of Clinical Pharmacology, 2004. 58: p. 25-33.

1021. Najib, N.M., et al., Comparison of two cyclosporine formulations in healthy Middle Eastern volunteers: bioequivalence of the new Sigmasporin Microoral and Sandimmun Neoral. European Journal of Pharmaceutics and Biopharmaceutics, 2003. 55(1): p. 67-70.

1022. Nakao, T., et al., Assessment of human exposure to PCDDs, PCDFs and Co-PCBs using hair as a human pollution indicator sample 1: development of analytical method for human hair and evaluation for exposure assessment. Chemosphere, 2002. 48(8): p. 885-896.

1023. Nakhostin-Roohi, B., et al., The Effect of Curcumin Supplementation on Selected Markers of Delayed Onset Muscle Soreness (DOMS). Annals of Applied Sport Science, 2016. 4(2): p. 25-31.

1024. Narahari, S.R., et al., Integrated management of filarial lymphedema for rural communities. Lymphology, 2007. 40(1): p. 3-13.

1025. Nassar, M., et al., Effect of phytic acid used as etchant on bond strength, smear layer, and pulpal cells. Eur J Oral Sci, 2013. 121(5): p. 482-7.

1026. Nassar, M., et al., Phytic acid: an alternative root canal chelating agent. J Endod, 2015. 41(2): p. 242-7.

1027. Nava, S., et al., EFFECT OF A BETA-2-AGONIST (BROXATEROL) ON RESPIRATORY MUSCLE STRENGTH AND ENDURANCE IN PATIENTS WITH COPD WITH IRREVERSIBLE AIRWAY-OBSTRUCTION. Chest, 1992. 101(1): p. 133-140.

1028. Nave, R., et al., Pharmacokinetics of C-14 ciclesonide after oral and intravenous administration to healthy subjects. Clinical Pharmacokinetics, 2004. 43(7): p. 479-486.

1029. Nawi, R.I.M., et al., Oral Cryotherapy Prevention of oral mucositis and pain among patients with colorectal cancer undergoing chemotherapy. Clinical Journal of Oncology Nursing, 2018. 22(5): p. 555-560.

1030. Nayak, S., et al., Comparative study of Candida by conventional and CHROMagar method in non-denture and denture wearers by oral rinse technique. Indian J Dent Res, 2012. 23(4): p. 490-7.

1031. Negoro, M., et al., Oral glucose retention, saliva viscosity and flow rate in 5-year-old children. Arch Oral Biol, 2000. 45(11): p. 1005-11.

1032. Neta, T., K. Takada, and M. Hirasawa, Low-cariogenicity of trehalose as a substrate. J Dent, 2000. 28(8): p. 571-6.

1033. Nettis, E., et al., Levocetirizine in the treatment of chronic idiopathic urticaria: a randomized, double-blind, placebo-controlled study. British Journal of Dermatology, 2006. 154(3): p. 533-538.

1034. Newcomer, J.W., et al., Decreased memory performance in healthy humans induced by stress-level cortisol treatment. Archives of General Psychiatry, 1999. 56(6): p. 527-533.

1035. Newhouse, P., et al., Tamoxifen Improves Cholinergically Modulated Cognitive Performance in Postmenopausal Women. Neuropsychopharmacology, 2013. 38(13): p. 2632-2643.

1036. Ng, M.G., et al., Exposure to Pesticides and Metal Contaminants of Fertilizer among Tree Planters. Annals of Occupational Hygiene, 2011. 55(7): p. 752-763.

1037. Nicol, L.M., et al., Curcumin supplementation likely attenuates delayed onset muscle soreness (DOMS). European Journal of Applied Physiology, 2015. 115(8): p. 1769-1777.

1038. Nilner, K., N. Vassilakos, and D. Birkhed, Effect of a buffering sugar-free lozenge on intraoral pH and electrochemical action. Acta Odontol Scand, 1991. 49(5): p. 267-72.

1039. Nilsson, U., et al., Cereal fructans: in vitro and in vivo studies on availability in rats and humans. J Nutr, 1988. 118(11): p. 1325-30.

1040. Ninomiya, Y., T. Imoto, and T. Sugimura, Sweet taste responses of mouse chorda tympani neurons: existence of gurmarin-sensitive and -insensitive receptor components. J Neurophysiol, 1999. 81(6): p. 3087-91.

1041. Nix, D.E., et al., Pharmacokinetics and relative bioavailability of clofazimine in relation to food, orange juice and antacid. Tuberculosis, 2004. 84(6): p. 365-373.

1042. Nix, D.E., et al., The effect of low-dose cimetidine (200 mg twice daily) on the pharmacokinetics of theophylline. Journal of Clinical Pharmacology, 1999. 39(8): p. 855-865.

1043. Nix, D.E., et al., EFFECT OF ORAL ANTACIDS ON DISPOSITION OF INTRAVENOUS ENOXACIN. Antimicrobial Agents and Chemotherapy, 1993. 37(4): p. 775-777.

1044. Nix, D.E., et al., Pharmacokinetics of Nikkomycin Z after Single Rising Oral Doses. Antimicrobial Agents and Chemotherapy, 2009. 53(6): p. 2517-2521.

1045. Noehr-Jensen, L., et al., The relative bioavailability of loratadine administered as a chewing gum formulation in healthy volunteers. European Journal of Clinical Pharmacology, 2006. 62(6): p. 437-445.

1046. Nokubi, T., et al., Fully automatic measuring system for assessing masticatory performance using beta-carotene-containing gummy jelly. Journal of Oral Rehabilitation, 2013. 40(2): p. 99-105.

1047. Noonan, P. and K. Flynn, Bounce Back. Men's Fitness, 2004. 20(4): p. 78-134.

1048. Noonan, P.K. and L.Z. Benet, Incomplete and delayed bioavailability of sublingual nitroglycerin. The American journal of cardiology, 1985. 55(1): p. 184-7.

1049. Norman, T.R., et al., The effect of single oral doses of zopiclone on nocturnal melatonin secretion in healthy male volunteers. Progress in Neuro-Psychopharmacology & Biological Psychiatry, 2001. 25(4): p. 825-833.

1050. Notarnicola, A., et al., Possible role of oral ibandronate administration in Osteonecrosis of the Jaw: a case report. International journal of immunopathology and pharmacology, 2012. 25(1): p. 311-6.

1051. Nottage, M., et al., Sucralfate mouthwash for prevention and treatment of 5-fluorouracil-induced mucositis: a randomized, placebo-controlled trial. Supportive care in cancer : official journal of the Multinational Association of Supportive Care in Cancer, 2003. 11(1): p. 41-7.

1052. Nuuja, M.C., et al., Effect of an experimental antiplaque preparation on salivary microbial counts in military academy cadets refraining from mechanical cleaning of the teeth. Mil Med, 1992. 157(3): p. 121-4.

1053. O'Donnell, R.L., et al., Physiological sex steroid replacement in premature ovarian failure: randomized crossover trial of effect on uterine volume, endometrial thickness and blood flow, compared with a standard regimen. Human Reproduction, 2012. 27(4): p. 1130-1138.

1054. O'Neal, E.K., et al., Post-prandial carbohydrate ingestion during 1-h of moderate-intensity, intermittent cycling does not improve mood, perceived exertion, or subsequent power output in recreationally-active exercisers. Journal of the International Society of Sports Nutrition, 2013. 10.

1055. Oberlin-Brown, K.T., et al., Oral Presence of Carbohydrate and Caffeine in Chewing Gum: Independent and Combined Effects on Endurance Cycling Performance. International Journal of Sports Physiology and Performance, 2016. 11(2): p. 164-171.

1056. Ogawa, A., et al., Effectiveness of a mouth rinsing function test for evaluating the oral function of children. Pediatric Dental Journal, 2017. 27(2): p. 85-93.

1057. Ogawa, H., M. Sato, and S. Yamashita, Gustatory impulse discharges in response to saccharin in rats and hamsters. J Physiol, 1969. 204(2): p. 311-29.

1058. Oginni, F.O., Dry Socket: A Prospective Study of Prevalent Risk Factors in a Nigerian Population. Journal of Oral and Maxillofacial Surgery, 2008. 66(11): p. 2290-2295.

1059. Ohara, S., et al., Comparison between a new 13C-urea breath test, using a film-coated tablet, and the conventional 13C-urea breath test for the detection of Helicobacter pylori infection. Journal of gastroenterology, 2004. 39(7): p. 621-8.

1060. Ohara, S., et al., Comparison between a new C-13-urea breath test, using a film-coated tablet, and the conventional C-13-urea breath test for the detection of Helicobacter pylori infection. Journal of Gastroenterology, 2004. 39(7): p. 621-628.

1061. Oliveira-Freitas, V.L., et al., Influence of Purple Grape Juice in Cyclosporine Bioavailability. Journal of Renal Nutrition, 2010. 20(5): p. 309-313.

1062. Oliver, E.T., et al., Effects of an Oral CRTh2 Antagonist (AZD1981) on Eosinophil Activity and Symptoms in Chronic Spontaneous Urticaria. International Archives of Allergy and Immunology, 2019. 179(1): p. 21-30.

1063. Oppermann, R.V., Effect of chlorhexidine on acidogenicity of dental plaque in vivo. Scand J Dent Res, 1979. 87(4): p. 302-8.

1064. Opstrup, M.S., et al., Chlorhexidine in cosmetic products - a market survey. Contact dermatitis, 2015. 72(1): p. 55-8.

1065. Oskoee, S.S., et al., Effect of composite resin contamination with powdered and unpowdered latex gloves on its shear bond strength to bovine dentin. Oper Dent, 2012. 37(5): p. 492-500.

1066. Osso, D. and N. Kanani, Antiseptic mouth rinses: an update on comparative effectiveness, risks and recommendations. J Dent Hyg, 2013. 87(1): p. 10-8.

1067. Ostman, J., et al., Comparison of effects of quinapril and metoprolol on glycaemic control, serum lipids, blood pressure, albuminuria and quality of life in non-insulin-dependent diabetes mellitus patients with hypertension. Journal of Internal Medicine, 1998. 244(2): p. 95-107.

1068. Ostojic, S.M., et al., Supplementation with Guanidinoacetic Acid in Women with Chronic Fatigue Syndrome. Nutrients, 2016. 8(2).

1069. Oxberry, S.G., et al., Short-term opioids for breathlessness in stable chronic heart failure: a randomized controlled trial. European Journal of Heart Failure, 2011. 13(9): p. 1006-1012.

1070. Ozan, G., H. Sar Sancakli, and T. Yucel, Effect of black tea and matrix metalloproteinase inhibitors on eroded dentin in situ. Microscopy research and technique, 2020. 83(7): p. 834-842.

1071. Ozdas, D.O. and M. Kazak, Colour preference between adults and children during a dental treatment session. Physiology & Behavior, 2017. 169: p. 165-168.

1072. Ozdemir, Z., et al., Investigation of pharmacokinetic interaction between ivermectin and praziquantel after oral administration in healthy dogs. Journal of Veterinary Pharmacology and Therapeutics, 2019. 42(5): p. 497-504.

1073. Padhi, D., M. Salfi, and M. Emery, Cinacalcet does not affect the activity of cytochrome P450 3A enzymes, a metabolic pathway for common immunosuppressive agents - A randomized, open-label, crossover, single-centre study in healthy volunteers. Drugs in R&D, 2008. 9(5): p. 335-343.

1074. Page, C.D., et al., COMPARATIVE PHARMACOKINETICS OF TRIMETHOPRIM-SULFAMETHOXAZOLE ADMINISTERED INTRAVENOUSLY AND ORALLY TO CAPTIVE ELEPHANTS. Journal of Zoo and Wildlife Medicine, 1991. 22(4): p. 409-416.

1075. Painelli, V.S., et al., The effect of carbohydrate mouth rinse on maximal strength and strength endurance. European journal of applied physiology, 2011. 111(9): p. 2381-6.

1076. Painelli, V.S., et al., The effect of carbohydrate mouth rinse on maximal strength and strength endurance. European Journal of Applied Physiology, 2011. 111(9): p. 2381-2386.

1077. Pak İ, E., et al., The effect of carbohydrate and caffeine mouth rinsing on kicking performance in competitive Taekwondo athletes during Ramadan. J Sports Sci, 2020. 38(7): p. 795-800.

1078. Pak, I.E., et al., The effect of carbohydrate and caffeine mouth rinsing on kicking performance in competitive Taekwondo athletes during Ramadan. Journal of sports sciences, 2020. 38(7): p. 795-800.

1079. Palma-Aguirre, J.A., et al., Bioavailability of Two Oral-Tablet and Two Oral-Suspension Formulations of Naproxen Sodium/Paracetamol (Acetaminophen): Single-Dose, Randomized, Open-Label, Two-Period Crossover Comparisons in Healthy Mexican Adult Subjects. Clinical Therapeutics, 2009. 31(2): p. 399-410.

1080. Palmer, M.S., et al., Ingesting A Sports Drink Enhances Simulated Ice Hockey Performance While Reducing Perceived Effort. International Journal of Sports Medicine, 2017. 38(14): p. 1061-1069.

1081. Palmer, W., License to Swill. Outside, 2010. 35(1): p. 60-60.

1082. Panzarini, S.R., et al., Use of vitamin C in delayed tooth replantation. Braz Dent J, 2005. 16(1): p. 17-22.

1083. Papas, A., et al., Caries clinical trial of a remineralising toothpaste in radiation patients. Gerodontology, 2008. 25(2): p. 76-88.

1084. Papp, J.R., et al., The use and performance of oral-throat rinses to detect pharyngeal Neisseria gonorrhoeae and Chlamydia trachomatis infections. Diagnostic microbiology and infectious disease, 2007. 59(3): p. 259-64.

1085. Papp, J.R., et al., The use and performance of oral-throat rinses to detect pharyngeal Neisseria gonorrhoeae and Chlamydia trachomatis infections. Diagnostic Microbiology and Infectious Disease, 2007. 59(3): p. 259-264.

1086. Paradisis, G., et al., Carbohydrate Mouth Rinse Does Not Affect 60-min Running Performance In Females. Medicine and Science in Sports and Exercise, 2017. 49(5): p. 581-581.

1087. Parikh-Das, A.M., et al., Superiority of essential oils versus 0.075% CPC-containing mouthrinse: a two-week randomized clinical trial. The Journal of clinical dentistry, 2013. 24(3): p. 94-9.

1088. Paris, H.L., et al., Effect of carbohydrate ingestion on central fatigue during prolonged running exercise in moderate hypoxia. Journal of Applied Physiology, 2019. 126(1): p. 141-151.

1089. Parise, C.V. and C. Spence, Assessing the associations between brand packaging and brand attributes using an indirect performance measure. Food Quality and Preference, 2012. 24(1): p. 17-23.

1090. Parisotto, R., et al., A novel method utilizing markers of altered erythropoiesis for the detection of recombinant human erythropoietin abuse in athletes. Haematologica, 2000. 85(6): p. 564-572.

1091. Park, J.S., et al., Preliminary bioequivalence of an oral integrating film formulation containing meloxicam with a suspension formulation in beagle dogs. Journal of Veterinary Pharmacology and Therapeutics, 2021. 44(3): p. 342-348.

1092. Park, K., et al., A randomized, open-label, two-period, crossover bioavailability study of two oral formulations of tacrolimus in healthy Korean adults. Clinical Therapeutics, 2007. 29(1): p. 154-162.

1093. Park, K.K., et al., The impact of chewing sugarless gum on the acidogenicity of fast-food meals. Am J Dent, 1990. 3(6): p. 231-5.

1094. Parker, R.B. and J.E. Soberman, Effects of Paroxetine on the Pharmacokinetics and Pharmacodynamics of Immediate-Release and Extended-Release Metoprolol. Pharmacotherapy, 2011. 31(7): p. 630-641.

1095. Parkinson, C., et al., A comparison of the effects of pegvisomant and octreotide on glucose, insulin, gastrin, cholecystokinin and pancreatic polypeptide responses to oral glucose and a standard mixed meal. Journal of Clinical Endocrinology & Metabolism, 2002. 87(4): p. 1797-1804.

1096. Pasquale, S.A., et al., Peripheral progesterone (P) levels and endometrial response to various dosages of vaginally administered P in estrogen-primed women. Fertility and Sterility, 1997. 68(5): p. 810-815.

1097. Pataky, M.W., et al., Caffeine and 3-km cycling performance: Effects of mouth rinsing, genotype, and time of day. Scandinavian Journal of Medicine & Science in Sports, 2016. 26(6): p. 613-619.

1098. Pataky, M.W., et al., Caffeine and 3-km cycling performance: Effects of mouth rinsing, genotype, and time of day. Scandinavian journal of medicine & science in sports, 2016. 26(6): p. 613-9.

1099. Patat, A., et al., EFFECTS OF ACUTE AND REPEATED DOSES OF 2 MUSCLE-RELAXANTS CHLORMEZANONE AND THIOCOLCHICOSIDE, ON VIGILANCE AND PSYCHOMOTOR PERFORMANCE OF HEALTHY-VOLUNTEERS. Human Psychopharmacology-Clinical and Experimental, 1991. 6(4): p. 285-292.

1100. Patat, A., et al., STUDY OF EFFECTS OF CLOBAZAM AND LORAZEPAM ON MEMORY AND COGNITIVE FUNCTIONS IN HEALTHY-SUBJECTS. Human Psychopharmacology-Clinical and Experimental, 1991. 6(3): p. 229-241.

1101. Patat, A., et al., LACK OF AMNESTIC, PSYCHOTOMIMETIC OR IMPAIRING EFFECT ON PSYCHOMOTOR PERFORMANCE OF ELIPRODIL, A NEW NMDA ANTAGONIST. International Clinical Psychopharmacology, 1994. 9(3): p. 155-162.

1102. Patat, A., et al., ASSESSMENT OF THE INTERACTION BETWEEN A PARTIAL AGONIST AND A FULL AGONIST OF BENZODIAZEPINE RECEPTORS, BASED ON PSYCHOMOTOR PERFORMANCE AND MEMORY, IN HEALTHY-VOLUNTEERS. Journal of Psychopharmacology, 1995. 9(2): p. 91-101.

1103. Patat, A., et al., LACK OF INTERACTION BETWEEN A NEW ANTIHISTAMINE, MIZOLASTINE, AND LORAZEPAM ON PSYCHOMOTOR PERFORMANCE AND MEMORY IN HEALTHY-VOLUNTEERS. British Journal of Clinical Pharmacology, 1995. 39(1): p. 31-38.

1104. Patat, A., et al., LACK OF INTERACTION BETWEEN 2 ANTIHISTAMINES, MIZOLASTINE AND CETIRIZINE, AND ETHANOL IN PSYCHOMOTOR AND DRIVING PERFORMANCE IN HEALTHY-SUBJECTS. European Journal of Clinical Pharmacology, 1995. 48(2): p. 143-150.

1105. Patel, D., et al., An atomic force microscopy investigation of bioadhesive polymer adsorption onto human buccal cells. Int J Pharm, 2000. 200(2): p. 271-7.

1106. Paterna, S., et al., Can losartan improve cardiac performance during the treadmill exercise test in hypertensive subjects? Drugs under Experimental and Clinical Research, 2002. 28(4): p. 155-159.

1107. Paunio, K., et al., Effects on oral health of mouthrinses containing xylitol, sodium cyclamate and sucrose sweeteners in the absence of oral hygiene. I. Clinical findings and analysis of gingival exudate. Proceedings of the Finnish Dental Society. Suomen Hammaslaakariseuran toimituksia, 1984. 80(1): p. 3-12.

1108. Pavone, C., et al., Prospective, randomized, crossover comparison of sublingual apomorphine (3 mg) with oral sildenafil (50 mg) for male erectile dysfunction. Journal of Urology, 2004. 172(6): p. 2347-2349.

1109. Pavone, C., et al., Prospective, randomized, crossover comparison of sublingual apomorphine (3 mg) with oral sildenafil (50 mg) for male erectile dysfunction (Reprinted from The Journal of Urology, vol 172, pg 2347-2349, 2007). Journal of Urology, 2008. 179(5): p. S92-S94.

1110. Pearce, E., Plaque minerals and dental caries. N Z Dent J, 1998. 94(415): p. 12-5.

1111. Pearce, E.I., H.C. Margolis, and R.L. Kent, Jr., Effect of in situ plaque mineral supplementation on the state of saturation of plaque fluid during sugar-induced acidogenesis. Eur J Oral Sci, 1999. 107(4): p. 251-9.

1112. Pearlstein, T.B., et al., Treatment of premenstrual dysphoric disorder with a new drospirenone-containing oral contraceptive formulation. Contraception, 2005. 72(6): p. 414-421.

1113. Peart, D.J., Quantifying the Effect of Carbohydrate Mouth Rinsing on Exercise Performance. Journal of strength and conditioning research, 2017. 31(6): p. 1737-1743.

1114. Peart, D.J., et al., The Effect of Carbohydrate Mouth Rinsing on Multiple Choice Reaction Time During Amateur Boxing. International journal of sports physiology and performance, 2020. 15(5): p. 720-723.

1115. Pedraz, J., et al., Sequential study on the treatment of moderate-to-severe chronic plaque psoriasis with mycophenolate mofetil and cyclosporin. Journal of the European Academy of Dermatology and Venereology, 2006. 20(6): p. 702-706.

1116. Perault, M.C., et al., Lack of interaction between amisulpride and lorazepam on psychomotor performance and memory in healthy volunteers. Human Psychopharmacology-Clinical and Experimental, 1998. 13(7): p. 493-500.

1117. Perdigao, J., et al., Effect of a hydrophobic bonding resin on the 36-month performance of a universal adhesive-a randomized clinical trial. Clinical Oral Investigations, 2020. 24(2): p. 765-776.

1118. Pereira, L.H., et al., Salivary markers and risk factor data: a multivariate modeling approach for head and neck squamous cell carcinoma detection. Cancer Biomark, 2011. 10(5): p. 241-9.

1119. Pereira, L.H., et al., Risk Stratification System for Oral Cancer Screening. Cancer Prev Res (Phila), 2016. 9(6): p. 445-55.

1120. Pereira, P.E.A., et al., Caffeine Supplementation or Carbohydrate Mouth Rinse Improves Performance. International journal of sports medicine, 2021. 42(2): p. 147-152.

1121. Perimenis, P., et al., Sildenafil combined with continuous positive airway pressure for treatment of erectile dysfunction in men with obstructive sleep apnea. International Urology and Nephrology, 2007. 39(2): p. 547-552.

1122. Pernu, H.E., U.H. Pajari, and M. Lanning, The importance of regular dental treatment in patients with cyclic neutropenia. Follow-up of 2 cases. Journal of periodontology, 1996. 67(4): p. 454-9.

1123. Perret, C., G. Mueller, and H. Knecht, Influence of creatine supplementation on 800m wheelchair performance: a pilot study. Spinal Cord, 2006. 44(5): p. 275-279.

1124. Person, B., et al., A Qualitative Evaluation of Hand Drying Practices among Kenyans. Plos One, 2013. 8(9).

1125. Persson, A., et al., Buffering effect of a prophylactic gel on dental plaque in institutionalised elderly. Gerodontology, 2007. 24(2): p. 98-104.

1126. Philip, G., et al., The Efficacy and Tolerability of Inhaled Montelukast Plus Inhaled Mometasone Compared with Mometasone Alone in Patients with Chronic Asthma. Journal of Asthma, 2011. 48(5): p. 495-502.

1127. Phillips, S.M., et al., The Influence of Serial Carbohydrate Mouth Rinsing on Power Output during a Cycle Sprint. Journal of Sports Science and Medicine, 2014. 13(2): p. 252-258.

1128. Phillips, S.M., J. Sproule, and A.P. Turner, Carbohydrate ingestion during team games exercise: current knowledge and areas for future investigation. Sports medicine (Auckland, N.Z.), 2011. 41(7): p. 559-85.

1129. Phillips, S.M., J. Sproule, and A.P. Turner, Carbohydrate Ingestion during Team Games Exercise. Sports Medicine, 2011. 41(7): p. 559-585.

1130. Pickering, C., Are caffeine's performance-enhancing effects partially driven by its bitter taste? Medical hypotheses, 2019. 131: p. 109301.

1131. Pietzsch, V. and H. Kuhl, CONTRACEPTION WITH CYCLIC TREATMENT OF BUSERELIN AND PROGESTOGENS - INCREASE IN SERUM CONCENTRATIONS OF TESTOSTERONE AND FREE TESTOSTERONE. Geburtshilfe Und Frauenheilkunde, 1991. 51(10): p. 819-823.

1132. Piltonen, T., et al., Oral, transdermal and vaginal combined contraceptives induce an increase in markers of chronic inflammation and impair insulin sensitivity in young healthy normal-weight women: a randomized study. Human Reproduction, 2012. 27(10): p. 3046-3056.

1133. Pineyro-Lopez, A., et al., Evaluation of the Bioequivalence of single 100-mg doses of two oral formulations of cyclosporin a microemulsion: A randomized, open-label, two-period crossover study in healthy adult male Mexican volunteers. Clinical Therapeutics, 2007. 29(9): p. 2049-2054.

1134. Pinheiro, I., et al., Effect of surface treatment and the use of mouthwashes on repaired composite bond strength. Revista Portuguesa De Estomatologia Medicina Dentaria E Cirurgia Maxilofacial, 2019. 60(3): p. 130-136.

1135. Pinheiro, L.C., et al., Oral nitrite circumvents antiseptic mouthwash-induced disruption of enterosalivary circuit of nitrate and promotes nitrosation and blood pressure lowering effect. Free radical biology & medicine, 2016. 101: p. 226-235.

1136. Pinto, G.S., et al., Effect of Yogurt Containing Bifidobacterium animalis subsp lactis DN-73010 Probiotic on Dental Plaque and Saliva in Orthodontic Patients. Caries Research, 2014. 48(1): p. 63-68.

1137. Piredda, M., et al., Propolis in the prevention of oral mucositis in breast cancer patients receiving adjuvant chemotherapy: A pilot randomised controlled trial. European journal of cancer care, 2017. 26(6).

1138. Pires, F.O., et al., Carbohydrate Mouth Rinse Fails to Improve Four-Kilometer Cycling Time Trial Performance. Nutrients, 2018. 10(3).

1139. Place, N., Go rinse your mouth: a novel way to improve endurance performance? J Physiol, 2009. 587(Pt 11): p. 2425-6.

1140. Plüss, E.M., P.R. Engelberger, and K.H. Rateitschak, Effect of chlorhexidine on dental plaque formation under periodontal pack. J Clin Periodontol, 1975. 2(3): p. 136-42.

1141. Pochmuller, M., et al., A systematic review and meta-analysis of carbohydrate benefits associated with randomized controlled competition-based performance trials. Journal of the International Society of Sports Nutrition, 2016. 13.

1142. Pokarier, M. and J.P. Gage, A laboratory study of the effect of thermocycling in saline and artificial saliva on two dentine bonding agents. Australian dental journal, 1989. 34(3): p. 266-71.

1143. Pomara, G., et al., Cardiovascular parameter changes in patients with erectile dysfunction using Pde-5 inhibitors: A study with sildenatil and vardenatil. Journal of Andrology, 2004. 25(4): p. 625-629.

1144. Pomara, N., et al., Increased anticholinergic challenge-induced memory impairment associated with the APOE-epsilon 4 allele in the elderly: A controlled pilot study. Neuropsychopharmacology, 2004. 29(2): p. 403-409.

1145. Pomportes, L., et al., Cognitive Performance Enhancement Induced by Caffeine, Carbohydrate and Guarana Mouth Rinsing during Submaximal Exercise. Nutrients, 2017. 9(6).

1146. Pomportes, L., et al., Effect of Carbohydrate Intake on Maximal Power Output and Cognitive Performances. Sports, 2016. 4(4).

1147. Ponce-Gonzalez, I.M., A.D. Cheadle, and M.L. Parchman, Correlation of Oral Health Education by Community Health Workers with Changes in Oral Health Practices in Migrant Populations in Washington State. Journal of Primary Care and Community Health, 2021. 12.

1148. Poo, J.L., et al., Bioavailability of two single-dose oral formulations of omeprazole 20 mg: An open-label, randomized sequence, two-period crossover comparison in healthy Mexican adult volunteers. Clinical Therapeutics, 2008. 30(4): p. 693-699.

1149. Poondru, S., et al., Chronopharmacokinetics of sumatriptan in healthy human subjects. Journal of Pharmacy and Pharmacology, 2000. 52(9): p. 1085-1090.

1150. Portela, A., et al., Spectral differences between epitopes in the cronomes of salivary CA130 and CA125. In Vivo, 1995. 9(4): p. 341-6.

1151. Potgieter, G.E., et al., Pharmacokinetics of pipamperone from three different tablet formulations. Arzneimittel-Forschung-Drug Research, 2002. 52(6): p. 430-434.

1152. Pottier, A., et al., Mouth rinse but not ingestion of a carbohydrate solution improves 1-h cycle time trial performance. Scandinavian journal of medicine & science in sports, 2010. 20(1): p. 105-11.

1153. Pozo, O.J., et al., Quantification of testosterone undecanoate in human hair by liquid chromatography-tandem mass spectrometry. Biomedical Chromatography, 2009. 23(8): p. 873-880.

1154. Preisser, J.S., et al., Marginalized zero-inflated negative binomial regression with application to dental caries. Statistics in medicine, 2016. 35(10): p. 1722-35.

1155. Prentice, A.G. and A. Glasmacher, Making sense of itraconazole pharmacokinetics. Journal of Antimicrobial Chemotherapy, 2005. 56: p. 17-22.

1156. Prescott, J., The generalizability of capsaicin sensitization and desensitization. Physiol Behav, 1999. 66(5): p. 741-9.

1157. Preshaw, P.M., et al., Effects of ketorolac tromethamine mouthrinse (0.1%) on crevicular fluid prostaglandin E-2, concentrations in untreated chronic periodontitis. Journal of Periodontology, 1998. 69(7): p. 777-783.

1158. Pribyslavska, V., et al., Influence of carbohydrate mouth rinsing on running and jumping performance during early morning soccer scrimmaging. European journal of sport science, 2016. 16(4): p. 441-7.

1159. Pribyslavska, V., et al., Influence of carbohydrate mouth rinsing on running and jumping performance during early morning soccer scrimmaging. European Journal of Sport Science, 2016. 16(4): p. 441-447.

1160. Prinz, J.F., L. Huntjens, and R.A. de Wijk, Instrumental and sensory quantification of oral coatings retained after swallowing semi-solid foods. Arch Oral Biol, 2006. 51(12): p. 1071-9.

1161. Procaccini, M., et al., [Flurbiprofen collutory in oral surgical pathology]. Flurbiprofen collutorio nella patologia chirurgica orale., 1996. 45(9): p. 421-5.

1162. Protoles, A., et al., Bioequivalence study of two formulations of Enalapril, at a single oral dose of 20 mg (Tablets): A randomized, two-way, open-label, crossover study in ealthy volunteers. Current Therapeutic Research-Clinical and Experimental, 2004. 65(1): p. 34-46.

1163. Puig-Silla, M., J.M. Montiel-Company, and J.M. Almerich-Silla, Comparison of the remineralizing effect of a sodium fluoride mouthrinse versus a sodium monofluorophosphate and calcium mouthrinse: an in vitro study. Med Oral Patol Oral Cir Bucal, 2009. 14(5): p. E257-62.

1164. Purkins, L., et al., Pharmacokinetics and safety of voriconazole following intravenous- to oral-dose escalation regimens. Antimicrobial Agents and Chemotherapy, 2002. 46(8): p. 2546-2553.

1165. Pyne, D.B. and W.G. Hopkins, Inadequate Sample Sizes in Studies of Athletic Performance at the 2012 ACSM Annual Meeting. Sportscience, 2012. 16: p. 1-11.

1166. Pyne, D.B., W.G. Hopkins, and D.T. Martin, Inadequate Sample Sizes in Studies of Athletic Performance at the 2012 ACSM Annual Meeting. Sportscience, 2010. 14: p. 1-11.

1167. Rabol, R., et al., Regional Anatomic Differences in Skeletal Muscle Mitochondrial Respiration in Type 2 Diabetes and Obesity. Journal of Clinical Endocrinology & Metabolism, 2010. 95(2): p. 857-863.

1168. Rafferty, A.P., et al., Diabetes Self-Care and Clinical Care Among Adults With Low Health Literacy. Journal of Public Health Management and Practice, 2021. 27(2): p. 144-153.

1169. Raitakari, O.T., et al., Coenzyme Q improves LDL resistance to ex vivo oxidation but does not enhance endothelial function in hypercholesterolemic young adults. Free Radical Biology and Medicine, 2000. 28(7): p. 1100-1105.

1170. Rajaie, S., et al., Moderate replacement of carbohydrates by dietary fats affects features of metabolic syndrome: A randomized crossover clinical trial. Nutrition, 2014. 30(1): p. 61-68.

1171. Raju, A.S., et al., The acidogenic potential of different milk formulas on dental plaque pH. Oral Health Prev Dent, 2012. 10(3): p. 225-30.

1172. Ramberg, P., et al., A model for studying the effects of mouthrinses on de novo plaque formation. Journal of clinical periodontology, 1992. 19(7): p. 509-20.

1173. Ramberg, P., et al., A MODEL FOR STUDYING THE EFFECTS OF MOUTHRINSES ON DENOVO PLAQUE-FORMATION. Journal of Clinical Periodontology, 1992. 19(7): p. 509-520.

1174. Ramirez, I., Intragastric feeding differentially affects apparent rate of gastric emptying of phenol red and carbohydrate. Physiol Behav, 1986. 36(5): p. 941-5.

1175. Randolph, C., et al., D-CYCLOSERINE TREATMENT OF ALZHEIMER-DISEASE. Alzheimer Disease & Associated Disorders, 1994. 8(3): p. 198-205.

1176. Rankine, C.A., et al., Biochemical comparison of plaque fluid on tooth and acrylic surfaces during a sucrose challenge. Arch Oral Biol, 1996. 41(7): p. 695-8.

1177. Rao, V.V.S., et al., Pharmacokinetics of a single dose of phenylpropanolamine following oral administration at two different times of the day. Arzneimittel-Forschung-Drug Research, 1998. 48(11): p. 1087-1090.

1178. Rautava, J., et al., An Oral Rinse Active Matrix Metalloproteinase-8 Point-of-Care Immunotest May Be Less Accurate in Patients with Crohn's Disease. Biomolecules, 2020. 10(3).

1179. Rawson, E.S., et al., Effects of repeated creatine supplementation on muscle, plasma, and urine creatine levels. Journal of Strength and Conditioning Research, 2004. 18(1): p. 162-167.

1180. Reale, R., G. Slater, and L.M. Burke, Individualised dietary strategies for Olympic combat sports: Acute weight loss, recovery and competition nutrition. European Journal of Sport Science, 2017. 17(6): p. 727-740.

1181. Rebello, S., et al., Effect of Cyclosporine on the Pharmacokinetics of Aliskiren in Healthy Subjects. Journal of Clinical Pharmacology, 2011. 51(11): p. 1549-1560.

1182. Reddy, R., et al., Evaluation of the Efficacy of Various Topical Fluorides on Enamel Demineralization Adjacent to Orthodontic Brackets: An In Vitro Study. The journal of contemporary dental practice, 2019. 20(1): p. 89-93.

1183. Rehage, M., et al., Oral astringent stimuli alter the enamel pellicle's ultrastructure as revealed by electron microscopy. J Dent, 2017. 63: p. 21-29.

1184. Rehman, S.U., K. Choe, and H.H. Yoo, Review on a Traditional Herbal Medicine, Eurycoma longifolia Jack (Tongkat Ali): Its Traditional Uses, Chemistry, Evidence-Based Pharmacology and Toxicology. Molecules, 2016. 21(3).

1185. Reiz, J.L., G.A.E. Donnelly, and K. Michalko, Comparative bioavailability of single-dose methylphenidate from a multilayer-release bead formulation and an osmotic system: A two-way crossover study in healthy young adults. Clinical Therapeutics, 2008. 30(1): p. 59-69.

1186. Rekola, M. and E. Söderling, Weekly variation in the acidogenic response of plaque. Acta Odontol Scand, 1990. 48(4): p. 229-32.

1187. Ren, Y.F., et al., Preventive effects of dentifrice containing 5000 ppm fluoride against dental erosion in situ. Journal of Dentistry, 2011. 39(10): p. 672-678.

1188. Revill, S.M. and M.D.L. Morgan, The cardiorespiratory response to submaximal exercise in subjects with asthma following pretreatment with controlled release oral salbutamol and high-dose inhaled salmeterol. Respiratory Medicine, 1998. 92(8): p. 1053-1058.

1189. Rezai, M.S., et al., Incidence, risk factors, and outcome of ventilator-associated Pneumonia in 18 hospitals of Iran. Running title: ventilator-associated pneumonia in Iran. International Journal of Advanced Biotechnology and Research, 2016. 7(3): p. 936-946.

1190. Rhim, S.Y., et al., Bioavailability and Bioequivalence of Two Oral Formulations of Alendronate Sodium 70 mg: An Open-Label, Randomized, Two-Period Crossover Comparison in Healthy Korean Adult Male Volunteers. Clinical Therapeutics, 2009. 31(5): p. 1037-1045.

1191. Rijkschroeff, P., et al., Oral polymorphonuclear neutrophil characteristics in relation to oral health: a cross-sectional, observational clinical study. International journal of oral science, 2016. 8(3): p. 191-8.

1192. Riley, S.P.D., J. Hadidian, and D.A. Manski, Population density, survival, and rabies in raccoons in an urban national park. Canadian Journal of Zoology, 1998. 76(6): p. 1153-1164.

1193. Rivollier, C., et al., Genital papules revealing a Churg-Strauss syndrome. Annales De Dermatologie Et De Venereologie, 2002. 129(8-9): p. 1049-1052.

1194. Rogers, S.N., et al., Health-related quality of life after maxillectomy: A comparison between prosthetic obturation and free flap. Journal of Oral and Maxillofacial Surgery, 2003. 61(2): p. 174-181.

1195. Rojas de Morales, T., et al., Oral-disease prevention in children with cancer: testing preventive protocol effectiveness. Medicina oral : organo oficial de la Sociedad Espanola de Medicina Oral y de la Academia Iberoamericana de Patologia y Medicina Bucal, 2001. 6(5): p. 326-34.

1196. Rojpibulstit, M., et al., Ambroxol lozenge bioavailability - An open-label, two-way crossover study of the comparative bioavailability of ambroxol lozenges and commercial tablets in healthy Thai volunteers. Clinical Drug Investigation, 2003. 23(4): p. 273-280.

1197. Rollo, I., et al., Influence of mouth rinsing a carbohydrate solution on 1-h running performance. Medicine and science in sports and exercise, 2010. 42(4): p. 798-804.

1198. Rollo, I., et al., Influence of mouth-rinsing a carbohydrate solution on 1-h running performance. Medicine & Science in Sports & Exercise, 2010. 42(4): p. 798-804.

1199. Rollo, I., et al., The Influence of Carbohydrate Mouth Rinse on Self-Selected Intermittent Running Performance. International journal of sport nutrition and exercise metabolism, 2015. 25(6): p. 550-8.

1200. Rollo, I. and C. Williams, Effect of mouth-rinsing carbohydrate solutions on endurance performance. Sports medicine (Auckland, N.Z.), 2011. 41(6): p. 449-61.

1201. Rollo, I., et al., The influence of carbohydrate mouth rinse on self-selected speeds during a 30-min treadmill run. International journal of sport nutrition and exercise metabolism, 2008. 18(6): p. 585-600.

1202. Rollo, I., et al., The Influence of Carbohydrate Mouth Rinse on Self-Selected Speeds During a 30-min Treadmill Run. International Journal of Sport Nutrition & Exercise Metabolism, 2008. 18(6): p. 585-600.

1203. Rollo, I., C. Williams, and M. Nevill, Influence of ingesting versus mouth rinsing a carbohydrate solution during a 1-h run. Medicine and science in sports and exercise, 2011. 43(3): p. 468-75.

1204. Romer, L.M., J.P. Barrington, and A.E. Jeukendrup, Effects of oral creatine supplementation on high intensity, intermittent exercise performance in competitive squash players. International Journal of Sports Medicine, 2001. 22(8): p. 546-552.

1205. Roque, L., et al., Mucoadhesive assessment of different antifungal nanoformulations. Bioinspir Biomim, 2018. 13(5): p. 055001.

1206. Rosenman, E.D., et al., A Simulation-based Approach to Measuring Team Situational Awareness in Emergency Medicine: A Multicenter, Observational Study. Academic Emergency Medicine, 2018. 25(2): p. 196-204.

1207. Rosenzweig, P., et al., ALPIDEM - LACK OF SEDATIVE EFFECT ON PSYCHOMOTOR PERFORMANCE IN THERAPEUTIC DOSES. Human Psychopharmacology-Clinical and Experimental, 1993. 8(6): p. 409-415.

1208. Rosin, M., et al., The effect of a dexibuprofen mouth rinse on experimental gingivitis in humans. Journal of clinical periodontology, 2005. 32(6): p. 617-21.

1209. Rosin, M., et al., Quantification of gingival edema using a new 3-D laser scanning method. Journal of Clinical Periodontology, 2002. 29(3): p. 240-246.

1210. Rossato, L.T., et al., No Improvement in Running Time to Exhaustion at 100% VO(2)max in Recreationally Active Male Runners With a Preexercise Single-Carbohydrate Mouth Rinse. International Journal of Sports Physiology and Performance, 2019. 14(9): p. 1178-1183.

1211. Rossman, E.I., et al., The gap junction modifier, GAP-134 [(2S,4R)-1-(2-aminoacetyl)-4-benzamido-pyrrolidine-2-carboxylic acid], improves conduction and reduces atrial fibrillation/flutter in the canine sterile pericarditis model. The Journal of pharmacology and experimental therapeutics, 2009. 329(3): p. 1127-33.

1212. Rouini, M.R., M. Dibaei, and E. Ghasemian, Pharmacokinetics and Bioequivalence Studies of Teriflunomide in Healthy Iranian Volunteers. Clinical Pharmacology in Drug Development, 2020. 9(3): p. 341-345.

1213. Rountree, J.A., et al., Efficacy of Carbohydrate Ingestion on CrossFit Exercise Performance. Sports, 2017. 5(3).

1214. Rowlatt, G., et al., The effect of carbohydrate mouth rinsing on fencing performance and cognitive function following fatigue-inducing fencing. European journal of sport science, 2017. 17(4): p. 433-440.

1215. Ruby, B.C., et al., Total energy expenditure during arduous wildfire suppression. Medicine and Science in Sports and Exercise, 2002. 34(6): p. 1048-1054.

1216. Rudin, C.M., et al., An attenuated adenovirus, ONYX-015, as mouthwash therapy for premalignant oral dysplasia. Journal of clinical oncology : official journal of the American Society of Clinical Oncology, 2003. 21(24): p. 4546-52.

1217. Rueda, J.R., et al., Myofunctional therapy (oropharyngeal exercises) for obstructive sleep apnoea. Cochrane Database of Systematic Reviews, 2020(11).

1218. Ruffino, J.S., et al., A comparison of the health benefits of reduced-exertion high-intensity interval training (REHIT) and moderate-intensity walking in type 2 diabetes patients. Applied Physiology Nutrition and Metabolism, 2017. 42(2): p. 202-208.

1219. Rugo, H.S., et al., Prevention of everolimus-related stomatitis in women with hormone receptor-positive, HER2-negative metastatic breast cancer using dexamethasone mouthwash (SWISH): a single-arm, phase 2 trial. The Lancet. Oncology, 2017. 18(5): p. 654-662.

1220. Ruiz, C., et al., Daily genital cares of female gynecologists: a descriptive study. Revista Da Associacao Medica Brasileira, 2019. 65(2): p. 171-176.

1221. Rumiantsev, V.A., et al., [Sugar of substitute stevioside in chewing gum: comparative double blind controllable study]. Stomatologiia (Mosk), 2011. 90(1): p. 18-21.

1222. Rundegren, J., et al., Effect of 4 days of mouth rinsing with delmopinol or chlorhexidine on the vitality of plaque bacteria. Journal of clinical periodontology, 1992. 19(5): p. 322-5.

1223. Ruschel, V.C., et al., Eighteen-month Clinical Study of Universal Adhesives in Noncarious Cervical Lesions. Operative Dentistry, 2018. 43(3): p. 241-249.

1224. Russell, M., et al., Half-Time Strategies to Enhance Second-Half Performance in Team-Sports Players: A Review and Recommendations. Sports Medicine, 2015. 45(3): p. 353-364.

1225. Saavedra, I., et al., Relative bioavailability study of two oral formulations of mycophenolate mofetil in healthy volunteers. Revista Medica De Chile, 2011. 139(7): p. 902-908.

1226. Safdi, M., et al., The effects of oral pancreatic enzymes (Creon 10 capsule) on steatorrhea - A multicenter, placebo-controlled, parallel group trial in subjects with chronic pancreatitis. Pancreas, 2006. 33(2): p. 156-162.

1227. Salehi, S., et al., Oral rehydration therapy products - a plaque pH study under normal and dry mouth conditions. Int Dent J, 2013. 63(5): p. 254-8.

1228. Salerno, S.M., J.L. Jackson, and E.P. Berbano, The impact of oral phenylpropanolamine on blood pressure: a meta-analysis and review of the literature. Journal of Human Hypertension, 2005. 19(8): p. 643-652.

1229. Saletu-Zyhlarz, G.M., et al., Double-Blind, Placebo-Controlled, Multiple-Ascending-Dose Study on the Pharmacodynamics of ABIO-08/01, a New CNS Drug with Potential Anxiolytic Activity 1. EEG Mapping, Psychometric and Tolerability Findings. Neuropsychobiology, 2009. 59(2): p. 100-109.

1230. Sallay, K., et al., [Etiology and prevention of recurring aphthous stomatitis]. Dtsch Zahnarztl Z, 1975. 30(9): p. 570-5.

1231. Salomone, A., et al., Occupational Exposure to Alcohol-Based Hand Sanitizers: The Diagnostic Role of Alcohol Biomarkers in Hair. Journal of analytical toxicology, 2018. 42(3): p. 157-162.

1232. Salyer, M., 'Let us wash the blood from your mouth': Revolutionary Horror and Lycanthropy in Frederick Marrvat's The Phantom Ship. Gothic Studies, 2018. 20(1-2): p. 95-110.

1233. Sampath, A., et al., A sensitive and a rapid multiplex polymerase chain reaction for the identification of Candida species in concentrated oral rinse specimens in patients with diabetes. Acta odontologica Scandinavica, 2017. 75(2): p. 113-122.

1234. Samuelson, S.T., et al., Simulation as a set-up for technical proficiency: can a virtual warm-up improve live fibre-optic intubation?(aEuro). British Journal of Anaesthesia, 2016. 116(3): p. 398-404.

1235. Sanders, M.A., et al., The gargle effect: rinsing the mouth with glucose enhances self-control. Psychol Sci, 2012. 23(12): p. 1470-2.

1236. Saneei, P., et al., The Dietary Approaches to Stop Hypertension (DASH) Diet Affects Inflammation in Childhood Metabolic Syndrome: A Randomized Cross-Over Clinical Trial. Annals of Nutrition and Metabolism, 2014. 64(1): p. 20-27.

1237. Sanematsu, K., et al., Molecular mechanisms for sweet-suppressing effect of gymnemic acids. J Biol Chem, 2014. 289(37): p. 25711-20.

1238. Sano, H., et al., Effect of rinsing with phosphorylated chitosan on four-day plaque regrowth. Bull Tokyo Dent Coll, 2001. 42(4): p. 251-6.

1239. Santhakumar, A.B., et al., The potential of anthocyanin-rich Queen Garnet plum juice supplementation in alleviating thrombotic risk under induced oxidative stress conditions. Journal of Functional Foods, 2015. 14: p. 747-757.

1240. Santi, E. and M. Bral, Effect of treatment on cyclosporine- and nifedipine-induced gingival enlargement: clinical and histologic results. The International journal of periodontics & restorative dentistry, 1998. 18(1): p. 80-5.

1241. Santiago, K.B., et al., Microbiological control and antibacterial action of a propolis-containing mouthwash and control of dental plaque in humans. Natural product research, 2018. 32(12): p. 1441-1445.

1242. Santos, V.R., et al., Full-mouth disinfection as a therapeutic protocol for type-2 diabetic subjects with chronic periodontitis: twelve-month clinical outcomes: a randomized controlled clinical trial. J Clin Periodontol, 2013. 40(2): p. 155-62.

1243. Saraceno, R., et al., Cyclosporine: a novel therapeutic approach for Burning Mouth Syndrome. Giornale italiano di dermatologia e venereologia : organo ufficiale, Societa italiana di dermatologia e sifilografia, 2016. 151(5): p. 480-4.

1244. Sari, I., et al., The effect of budesonide mouthwash on oral chronic graft versus host disease. American journal of hematology, 2007. 82(5): p. 349-56.

1245. Sartini, I., et al., Pharmacokinetic profiles of meloxicam after single IV and PO administration in Bilgorajska geese. Journal of Veterinary Pharmacology and Therapeutics, 2020. 43(1): p. 26-32.

1246. Sato, T. and L.M. Beidler, The response characteristics of rat taste cells to four basic taste stimuli. Comp Biochem Physiol A Comp Physiol, 1982. 73(1): p. 1-10.

1247. Sattar, A., et al., Pharmacokinetics and Metabolism of Cyadox and Its Main Metabolites in Beagle Dogs Following Oral, Intramuscular, and Intravenous Administration. Frontiers in Pharmacology, 2016. 7.

1248. Sauder, K.A., et al., Effects of pistachios on the lipid/lipoprotein profile, glycemic control, inflammation, and endothelial function in type 2 diabetes: A randomized trial. Metabolism-Clinical and Experimental, 2015. 64(11): p. 1521-1529.

1249. Saunders, D.P., et al., Systematic review of antimicrobials, mucosal coating agents, anesthetics, and analgesics for the management of oral mucositis in cancer patients. Support Care Cancer, 2013. 21(11): p. 3191-207.

1250. Saunders, M.J., et al., Caffeine Mouth Rinsing in the Fed State Does Not Enhance 3-km Cycling Performance: 2406 Board #242 June 1 11:00 AM - 12:30 PM. Medicine & Science in Sports & Exercise, 2018. 50: p. 596-596.

1251. Sawicki, C.M., et al., Phytochemical Pharmacokinetics and Bioactivity of Oat and Barley Flour: A Randomized Crossover Trial. Nutrients, 2016. 8(12).

1252. Sbarouni, E., et al., Estrogen replacement therapy and exercise performance in postmenopausal women with coronary artery disease. American Journal of Cardiology, 1997. 79(1): p. 87-&.

1253. Sbarouni, E., et al., Absence of effects of short-term estrogen replacement therapy on resting and exertional QT and QT(c) dispersion in postmenopausal women with coronary artery disease. Pace-Pacing and Clinical Electrophysiology, 1998. 21(11): p. 2392-2395.

1254. Schaffler, K., D. Hadler, and M. Stark, Dose-effect relationship of idebenone in an experimental cerebral deficit model - Pilot study in healthy young volunteers with piracetam as reference drug. Arzneimittel-Forschung-Drug Research, 1998. 48(7): p. 720-726.

1255. Schaffler, K., et al., Effect of the new H-1-antagonist ReN1869 on capsaicin-induced hyperalgesia in human skin - Human phase-I trial using somatosensory evoked potentials induced by a CO2 laser. Arzneimittelforschung-Drug Research, 2004. 54(3): p. 187-191.

1256. Scheie, A.A., et al., Use of palladium touch microelectrodes under field conditions for in vivo assessment of dental plaque pH in children. Caries Res, 1992. 26(1): p. 44-51.

1257. Schemehorn, B.R., et al., A new model for demonstrating enamel protection benefits relative to acid challenge. J Clin Dent, 2013. 24(2): p. 49-54.

1258. Schenk, J.F., et al., Antiplatelet and anticoagulant effects of "HN-11 500," a selective thromboxane receptor antagonist. Thrombosis Research, 2001. 103(2): p. 79-91.

1259. Schestakow, A., et al., Evaluation of Anti-Biofilm Activity of Mouthrinses Containing Tannic Acid or Chitosan on Dentin In Situ. Molecules, 2021. 26(5).

1260. Schiff, M., et al., Platelet inhibitory effects of OTC doses of naproxen sodium compared with prescription dose naproxen sodium and low-dose aspirin. Current Medical Research and Opinion, 2009. 25(10): p. 2471-2477.

1261. Schlueter, N., et al., Effect of pepsin on erosive tissue loss and the efficacy of fluoridation measures in dentine in vitro. Acta odontologica Scandinavica, 2007. 65(5): p. 298-305.

1262. Schlueter, N., J. Klimek, and C. Ganss, In vitro efficacy of experimental tin- and fluoride-containing mouth rinses as anti-erosive agents in enamel. Journal of dentistry, 2009. 37(12): p. 944-8.

1263. Schlueter, N., J. Klimek, and C. Ganss, Effect of stannous and fluoride concentration in a mouth rinse on erosive tissue loss in enamel in vitro. Archives of oral biology, 2009. 54(5): p. 432-6.

1264. Schlueter, N., et al., Tin and fluoride as anti-erosive agents in enamel and dentine in vitro. Acta odontologica Scandinavica, 2010. 68(3): p. 180-4.

1265. Schmitt, J.A., Epidemiological investigations or oral Candida albicans. Mycopathologia et mycologia applicata, 1971. 43(1): p. 65-87.

1266. Schmitz, N.S., et al., A Randomized Dose Escalation Study of Intravenous Baclofen in Healthy Volunteers: Clinical Tolerance and Pharmacokinetics. Pm&R, 2017. 9(8): p. 743-750.

1267. Schnitzler, C., L. Seifert, and D. Chollet, Arm Coordination and Performance Level in the 400-m Front Crawl. Research Quarterly for Exercise & Sport, 2011. 82(1): p. 1-8.

1268. Schou, A.J., C. Heuck, and O.D. Wolthers, Does vitamin D administered to children with asthma treated with inhaled glucocorticoids affect short-term growth or bone turnover? Pediatric Pulmonology, 2003. 36(5): p. 399-404.

1269. Schran, H.F., et al., The pharmacokinetics and bioavailability of clemastine and phenylpropanolamine in single-component and combination formulations. Journal of Clinical Pharmacology, 1996. 36(10): p. 911-922.

1270. Schrenzel, J., et al., INFLUENCE OF RIFAMPIN ON FLEROXACIN PHARMACOKINETICS. Antimicrobial Agents and Chemotherapy, 1993. 37(10): p. 2132-2138.

1271. Schussler, P., et al., Progesterone reduces wakefulness in sleep EEG and has no effect on cognition in healthy postmenopausal women. Psychoneuroendocrinology, 2008. 33(8): p. 1124-1131.

1272. Seaber, E.J., et al., Lack of interaction between pizotifen and the novel antimigraine compound zolmitriptan in healthy volunteers. Clinical Drug Investigation, 1997. 14(3): p. 221-225.

1273. Seidegard, J., M. Simonsson, and S. Edsbacker, Effect of an oral contraceptive on the plasma levels of budesonide and prednisolone and the influence on plasma cortisol. Clinical Pharmacology & Therapeutics, 2000. 67(4): p. 373-381.

1274. Seignemartin, C.P., et al., Understandability of Speech Predicts Quality of Life Among Maxillectomy Patients Restored With Obturator Prosthesis. Journal of Oral and Maxillofacial Surgery, 2015. 73(10): p. 2040-2048.

1275. Seino, Y., et al., Safety and efficacy of semaglutide once weekly vs sitagliptin once daily, both as monotherapy in Japanese people with type 2 diabetes. Diabetes Obesity & Metabolism, 2018. 20(2): p. 378-388.

1276. Sekine, I., et al., [Effectiveness of guaiazulene in orodental field--gargling or retaining in mouth]. Shikai tenbo = Dental outlook, 1981. 58(3): p. 572-81.

1277. Sen, N., et al., Nebivolol therapy improves endothelial function and increases exercise tolerance in patients with cardiac syndrome X. Anadolu Kardiyoloji Dergisi-the Anatolian Journal of Cardiology, 2009. 9(5): p. 371-379.

1278. Senghore, N. and M. Harris, The effect of tranexamic acid (cyclokapron) on blood loss after third molar extraction under a day case general anaesthetic. British Dental Journal, 1999. 186(12): p. 634-636.

1279. Sereda, G. and S. Saeedi, Pre-treatment of dentin with chondroitin sulfate or L-arginine modulates dentin tubule occlusion by toothpaste components. Am J Dent, 2019. 32(2): p. 81-88.

1280. Shaefer, M.S., et al., EVALUATION OF THE PHARMACOKINETIC INTERACTION BETWEEN CIMETIDINE OR FAMOTIDINE AND CYCLOSPORINE IN HEALTHY-MEN. Annals of Pharmacotherapy, 1995. 29(11): p. 1088-1091.

1281. Shaik, N., et al., Absolute Oral Bioavailability of Glasdegib (PF-04449913), a Smoothened Inhibitor, in Randomized Healthy Volunteers. Clinical Pharmacology in Drug Development, 2019. 8(7): p. 895-902.

1282. Shannon, I.L., Effect of whole saliva on performance of 0.1 per cent stannous fluoride mouthwash. SAM-TR-65-50. [Technical report] SAM-TR. USAF School of Aerospace Medicine, 1965: p. 1-5.

1283. Shanon, A., et al., COMPARISON OF CNS ADVERSE-EFFECTS BETWEEN ASTEMIZOLE AND CHLORPHENIRAMINE IN CHILDREN - A RANDOMIZED, DOUBLE-BLIND-STUDY. Developmental Pharmacology and Therapeutics, 1993. 20(3-4): p. 239-246.

1284. Sharma, M., et al., Optical pH measurement system using a single fluorescent dye for assessing susceptibility to dental caries. J Biomed Opt, 2019. 24(1): p. 1-8.

1285. Shaver, L.N., et al., No Performance or Affective Advantage of Drinking versus Rinsing with Water during a 15-km Running Session in Female Runners. International Journal of Exercise Science, 2018. 11(2): p. 910-920.

1286. Shechter, M., et al., Comparison of magnesium status using X-ray dispersion analysis following magnesium oxide and magnesium citrate treatment of healthy subjects. Magnesium Research, 2012. 25(1): p. 28-39.

1287. Shen, S., et al., Targeting Viral DNA and Promoter Hypermethylation in Salivary Rinses for Recurrent HPV-Positive Oropharyngeal Cancer. Otolaryngology--head and neck surgery : official journal of American Academy of Otolaryngology-Head and Neck Surgery, 2020. 162(4): p. 512-519.

1288. Shen, S., et al., Targeting Viral DNA and Promoter Hypermethylation in Salivary Rinses for Recurrent HPV-Positive Oropharyngeal Cancer. Otolaryngology-Head and Neck Surgery, 2020. 162(4): p. 512-519.

1289. Shern, R.J., B.B. Rundell, and C.J. Defever, Effects of an amine fluoride mouthrinse on the formation and microbial content of plaque. Helvetica odontologica acta, 1974. 18: p. Suppl-62.

1290. Shi, J., et al., Pharmacokinetics of sematilide in renal failure. Journal of Clinical Pharmacology, 1996. 36(2): p. 131-143.

1291. Shiba, F., et al., Anti-inflammatory effect of glycyrrhizin with Equisetum arvense extract. Odontology, 2021. 109(2): p. 464-473.

1292. Shim, E. and J.P. Babu, Glycated albumin produced in diabetic hyperglycemia promotes monocyte secretion of inflammatory cytokines and bacterial adherence to epithelial cells. J Periodontal Res, 2015. 50(2): p. 197-204.

1293. Shinada, K., et al., Hop bract polyphenols reduced three-day dental plaque regrowth. Journal of dental research, 2007. 86(9): p. 848-51.

1294. Ship, J.A., et al., Safety and effectiveness of topical dry mouth products containing olive oil, betaine, and xylitol in reducing xerostomia for polypharmacy-induced dry mouth. J Oral Rehabil, 2007. 34(10): p. 724-32.

1295. Shmagel, A., et al., Population-Wide Associations between Common Viral Pathogens and Self-Reported Arthritis: NHANES 2009-2012. International Journal of Rheumatology, 2018. 2018.

1296. Shohag, M.H., et al., Pharmacokinetic and bioequivalence study of etoricoxib tablet in healthy Bangladeshi volunteers. Arzneimittelforschung-Drug Research, 2011. 61(11): p. 617-621.

1297. Shon, J.H., et al., Effect of itraconazole on the pharmacokinetics and pharmacodynamics of fexofenadine in relation to the MDR1 genetic polymorphism. Clinical Pharmacology & Therapeutics, 2005. 78(2): p. 191-201.

1298. Shu, I., et al., Determination of methamphetamine enantiomer composition in human hair by non-chiral liquid chromatography-tandem mass spectrometry method. Journal of Chromatography B-Analytical Technologies in the Biomedical and Life Sciences, 2016. 1028: p. 145-152.

1299. Shumaker, R.C., et al., Effect of Rifampicin on the Pharmacokinetics of Lenvatinib in Healthy Adults. Clinical Drug Investigation, 2014. 34(9): p. 651-659.

1300. Shuster, D.L., et al., Maternal-Fetal Disposition of Glyburide in Pregnant Mice Is Dependent on Gestational Ages. Journal of Pharmacology and Experimental Therapeutics, 2014. 350(2): p. 425-434.

1301. Shuto, H., et al., [A case of the esophageal candidiasis supposedly caused by rhinenchysis steroid chronic administration before sleep]. Arerugi = [Allergy], 2007. 56(7): p. 714-20.

1302. Shyu, W.C., C.R. Gleason, and R.H. Barbhaiya, EFFECTS OF TIME OF ADMINISTRATION AND POSTURE ON THE PHARMACOKINETICS OF CEFPROZIL. Clinical Pharmacokinetics, 1993. 25(3): p. 237-242.

1303. Si, T.M., et al., Efficacy and safety of flexibly dosed paliperidone palmitate in Chinese patients with acute schizophrenia: an open-label, single-arm, prospective, interventional study. Neuropsychiatric Disease and Treatment, 2015. 11: p. 1483-1492.

1304. Siddeshappa, S.T., et al., Comparative evaluation of antiplaque and antigingivitis effects of an herbal and chlorine dioxide mouthwashes: A clinicomicrobiological study. Indian journal of dental research : official publication of Indian Society for Dental Research, 2018. 29(1): p. 34-40.

1305. Siegel, R. and P.B. Laursen, Keeping Your Cool Possible Mechanisms for Enhanced Exercise Performance in the Heat with Internal Cooling Methods. Sports Medicine, 2012. 42(2): p. 89-98.

1306. Siegel, R., et al., The influence of ice slurry ingestion on maximal voluntary contraction following exercise-induced hyperthermia. European Journal of Applied Physiology, 2011. 111(10): p. 2517-2524.

1307. Siegmund, W., et al., Variability of intestinal expression of P-glycoprotein in healthy volunteers as described by absorption of talinolol from four bioequivalent tablets. Journal of Pharmaceutical Sciences, 2003. 92(3): p. 604-610.

1308. Silva, A.M., et al., Electric toothbrush for biofilm control in individuals with Down syndrome: a crossover randomized clinical trial. Brazilian Oral Research, 2020. 34.

1309. Silveira, P., et al., Psychomotor effects of mexazolam vs placebo in healthy volunteers. Clinical Drug Investigation, 2002. 22(10): p. 677-684.

1310. Silvestre, R., et al., Effects of exercise at different times on postprandial lipemia and endothelial function. Medicine and Science in Sports and Exercise, 2008. 40(2): p. 264-274.

1311. Simon, S., et al., Bioavailability of Oral Carisoprodol 250 and 350 mg and Metabolism to Meprobamate: A Single-Dose Crossover Study. Current Therapeutic Research-Clinical and Experimental, 2010. 71(1): p. 50-59.

1312. Simonsen, L., L.H. Enevoldsen, and J. Bulow, Determination of adipose tissue blood flow with local Xe-133 clearance. Evaluation of a new labelling technique. Clinical Physiology and Functional Imaging, 2003. 23(6): p. 320-323.

1313. Simonsson, T., et al., Effect of delmopinol on in vitro dental plaque formation, bacterial acid production and the number of microorganisms in human saliva. Oral Microbiol Immunol, 1991. 6(5): p. 305-9.

1314. Simpson, G.W., et al., Carbohydrate Mouth Rinse Improves Relative Mean Power During Multiple Sprint Performance. Int J Exerc Sci, 2018. 11(6): p. 754-763.

1315. Simpson, G.W., et al., Carbohydrate Mouth Rinse Improves Relative Mean Power During Multiple Sprint Performance. International Journal of Exercise Science, 2018. 11(6): p. 754-763.

1316. Sinclair, J., et al., The effect of different durations of carbohydrate mouth rinse on cycling performance. European journal of sport science, 2014. 14(3): p. 259-64.

1317. Sindet-Pedersen, S., Distribution of tranexamic acid to plasma and saliva after oral administration and mouth rinsing: a pharmacokinetic study. Journal of clinical pharmacology, 1987. 27(12): p. 1005-8.

1318. Sindet-Pedersen, S., et al., Hemostatic effect of tranexamic acid mouthwash in anticoagulant-treated patients undergoing oral surgery. The New England journal of medicine, 1989. 320(13): p. 840-3.

1319. Siqueira, W.L., et al., Quantitative Proteomic Analysis of the Effect of Fluoride on the Acquired Enamel Pellicle. Plos One, 2012. 7(8).

1320. Sitruk-Ware, R., et al., Effects of oral and transvaginal ethinyl estradiol on hemostatic factors and hepatic proteins in a randomized, crossover study. Journal of Clinical Endocrinology & Metabolism, 2007. 92(6): p. 2074-2079.

1321. Skjørland, K.K. and T. Sønju, Effect of sucrose rinses on bacterial colonization on amalgam and composite. Acta Odontol Scand, 1982. 40(4): p. 193-6.

1322. Slattery, K.M., et al., Effect of N-acetylcysteine on Cycling Performance after Intensified Training. Medicine and Science in Sports and Exercise, 2014. 46(6): p. 1114-1123.

1323. Smart, J.D., et al., A quantitative evaluation of radiolabelled lectin retention on oral mucosa in vitro and in vivo. European Journal of Pharmaceutics and Biopharmaceutics, 2002. 53(3): p. 289-292.

1324. Smith, B.J., S.M. Kirschner, and L.V. Kendall, Pharmacokinetics of Sustained-release, Oral, and Subcutaneous Meloxicam over 72 Hours in Male Beagle Dogs. Journal of the American Association for Laboratory Animal Science, 2020. 59(6): p. 737-741.

1325. Smith, S.A., et al., Plasma pharmacokinetics of warfarin enantiomers in cats. Journal of Veterinary Pharmacology and Therapeutics, 2000. 23(6): p. 329-337.

1326. Smolders, R.G.V., et al., A randomized placebo-controlled study of the effect of transdermal vs. oral estradiol with or without gestodene on homocysteine levels. Fertility and Sterility, 2003. 79(2): p. 261-267.

1327. Smolensky, M.H., R.C. Hermida, and F. Portaluppi, Comparison of the efficacy of morning versus evening administration of olmesartan in uncomplicated essential hypertension. Chronobiology International, 2007. 24(1): p. 171-181.

1328. Smutzer, G., et al., Detection and modulation of capsaicin perception in the human oral cavity. Physiol Behav, 2018. 194: p. 120-131.

1329. Solis, C., et al., 0.2% chlorhexidine mouthwash with an antidiscoloration system versus 0.2% chlorhexidine mouthwash: a prospective clinical comparative study. Journal of periodontology, 2011. 82(1): p. 80-5.

1330. Sollars, S.I. and D.L. Hill, Taste responses in the greater superficial petrosal nerve: substantial sodium salt and amiloride sensitivities demonstrated in two rat strains. Behav Neurosci, 1998. 112(4): p. 991-1000.

1331. Sonet, B., et al., Randomised crossover studies of the bioequivalence of two fenofibrate formulations after administration of a single oral dose in healthy volunteers. Arzneimittel-Forschung-Drug Research, 2002. 52(3): p. 200-204.

1332. Sorensen, J.B., et al., Double-blind, placebo-controlled, randomized study of chlorhexidine prophylaxis for 5-fluorouracil-based chemotherapy-induced oral mucositis with nonblinded randomized comparison to oral cooling (cryotherapy) in gastrointestinal malignancies. Cancer, 2008. 112(7): p. 1600-6.

1333. Sorensen, M.B., et al., Measurement of aortic blood flow by magnetic resonance below and above the origin of the coronary arteries in postmenopausal hormone replacement therapy. Journal of Cardiovascular Magnetic Resonance, 2004. 6(3): p. 637-644.

1334. Sorvari, R., S. Spets-Happonen, and H. Luoma, Efficacy of chlorhexidine solution with fluoride varnishing in preventing enamel softening by Streptococcus mutans in an artificial mouth. Scand J Dent Res, 1994. 102(4): p. 206-9.

1335. Souza, J.G., et al., Calcium Prerinse before Fluoride Rinse Reduces Enamel Demineralization: An in situ Caries Study. Caries Res, 2016. 50(4): p. 372-7.

1336. Souza, M.J., et al., Pharmacokinetics after oral and intravenous administration of a single dose of tramadol hydrochloride to Hispaniolan Amazon parrots (Amazona ventralis). American Journal of Veterinary Research, 2012. 73(8): p. 1142-1147.

1337. Sowunmi, A. and L.A. Salako, Effect of dose size on the pharmacokinetics of orally administered quinine. European Journal of Clinical Pharmacology, 1996. 49(5): p. 383-386.

1338. Spijkervet, F.K., et al., Mucositis prevention by selective elimination of oral flora in irradiated head and neck cancer patients. J Oral Pathol Med, 1990. 19(10): p. 486-9.

1339. Spindle, T.R., et al., Acute Effects of Smoked and Vaporized Cannabis in Healthy Adults Who Infrequently Use Cannabis A Crossover Trial. Jama Network Open, 2018. 1(7).

1340. Spinelli, M.G., Postpartum Psychosis: Detection of Risk and Management. American Journal of Psychiatry, 2009. 166(4): p. 405-408.

1341. Sreebny, L.M., R. Chatterjee, and I. Kleinberg, Clearance of glucose and sucrose from the saliva of human subjects. Arch Oral Biol, 1985. 30(3): p. 269-74.

1342. Srivaths, L.V., et al., Oral Tranexamic Acid versus Combined Oral Contraceptives for Adolescent Heavy Menstrual Bleeding: A Pilot Study. Journal of Pediatric and Adolescent Gynecology, 2015. 28(4): p. 254-257.

1343. Stalker, D.J., I.R. Welshman, and S.R. Pollock, BIOAVAILABILITY OF MEDROXYPROGESTERONE ACETATE FROM 3 ORAL DOSAGE FORMULATIONS. Clinical Therapeutics, 1992. 14(4): p. 544-552.

1344. Stallard, R.E., et al., The effect of an antimicrobial mouth rinse on dental plaque, calculus and gingivitis. J Periodontal Res Suppl, 1969(4): p. 37-8.

1345. Stanelle, S.T., K.L. McLaughlin, and S.F. Crouse, One Week of L-Citrulline Supplementation Improves Performance in Trained Cyclists. Journal of Strength and Conditioning Research, 2020. 34(3): p. 647-652.

1346. Stanger, O., et al., Effects of folate treatment and homocysteine lowering on resistance vessel reactivity in atherosclerotic subjects. Journal of Pharmacology and Experimental Therapeutics, 2002. 303(1): p. 158-162.

1347. Stass, H. and R. Sachse, Effect of probenecid on the kinetics of a single oral 400mg dose of moxifloxacin in healthy male volunteers. Clinical Pharmacokinetics, 2001. 40: p. 71-76.

1348. Staudacher, H.M., et al., Short-term high-fat diet alters substrate utilization during exercise but not glucose tolerance in highly trained athletes. International Journal of Sport Nutrition and Exercise Metabolism, 2001. 11(3): p. 273-286.

1349. Stebler, T. and T.W. Guentert, BIOAVAILABILITY OF INTRAMUSCULARLY ADMINISTERED TENOXICAM. Biopharmaceutics & Drug Disposition, 1993. 14(6): p. 483-490.

1350. Steele, I.C., V.H. Patterson, and D.P. Nicholls, A double blind, placebo controlled, crossover trial of D-ribose in McArdle's disease. Journal of the Neurological Sciences, 1996. 136(1-2): p. 174-177.

1351. Stekler, J.D., et al., Performance of Determine Combo and other point-of-care HIV tests among Seattle MSM. Journal of Clinical Virology, 2016. 76: p. 8-13.

1352. Stellingwerff, T. and G.R. Cox, Systematic review: Carbohydrate supplementation on exercise performance or capacity of varying durations. Applied physiology, nutrition, and metabolism = Physiologie appliquee, nutrition et metabolisme, 2014. 39(9): p. 998-1011.

1353. Stellingwerff, T. and G.R. Cox, Systematic review: Carbohydrate supplementation on exercise performance or capacity of varying durations1. Applied Physiology, Nutrition & Metabolism, 2014. 39(9): p. 998-1011.

1354. Stephen, K.W., Dentifrices: recent clinical findings and implications for use. International dental journal, 1993. 43(6 Suppl 1): p. 549-53.

1355. Stevens, C. and R. Best, Menthol: A Fresh Ergogenic Aid for Athletic Performance. Sports Medicine, 2017. 47(6): p. 1035-1042.

1356. Stevens, C.D., Immediate shear bond strength of resin cements to sodium hypochlorite-treated dentin. J Endod, 2014. 40(9): p. 1459-62.

1357. Stevens, C.J., et al., A Comparison of Mixed-Method Cooling Interventions on Preloaded Running Performance in the Heat. J Strength Cond Res, 2017. 31(3): p. 620-629.

1358. Stevens, C.J., et al., A Comparison of Mixed-Method Cooling Interventions on Preloaded Running Performance in the Heat. Journal of strength and conditioning research, 2017. 31(3): p. 620-629.

1359. Stevens, C.J., et al., A COMPARISON OF MIXED-METHOD COOLING INTERVENTIONS ON PRELOADED RUNNING PERFORMANCE IN THE HEAT. Journal of Strength & Conditioning Research (Lippincott Williams & Wilkins), 2017. 31(3): p. 620-629.

1360. Stevens, C.J. and R. Best, Menthol: A Fresh Ergogenic Aid for Athletic Performance. Sports medicine (Auckland, N.Z.), 2017. 47(6): p. 1035-1042.

1361. Stevens, C.J., et al., Running performance and thermal sensation in the heat are improved with menthol mouth rinse but not ice slurry ingestion. Scandinavian journal of medicine & science in sports, 2016. 26(10): p. 1209-16.

1362. Stockis, A., S. Watanabe, and N. Fauchoux, Interaction between brivaracetam ( 100 mg/ day) and a combination oral contraceptive: A randomized, doubleblind, placebo-controlled study. Epilepsia, 2014. 55(3): p. E27-E31.

1363. Stokman, M.A., et al., Outcome of local application of amifostine (WR-1065) on epirubicin-induced oral mucositis. A phase II study. Anticancer Res, 2004. 24(5b): p. 3263-7.

1364. Stokman, M.A., et al., Outcome of local application of amifostine (WR-1065) on epirubicin-induced oral mucositis. A phase II study. Anticancer research, 2004. 24(5B): p. 3263-7.

1365. Stolen, M., CODESWITCHING FOR HUMOR AND ETHNIC-IDENTITY - WRITTEN DANISH AMERICAN OCCASIONAL SONGS. Journal of Multilingual and Multicultural Development, 1992. 13(1-2): p. 215-228.

1366. Stoppani, J., THE EDGE. Joe Weider's Muscle & Fitness, 2006. 67(7): p. 237-238.

1367. Strandgarden, K., et al., Dissolution rate-limited absorption and complete bioavailability of roquinimex in man. Biopharmaceutics & Drug Disposition, 1999. 20(7): p. 347-354.

1368. Stratford, M.R., et al., Nicotinamide pharmacokinetics in humans: effect of gastric acid inhibition, comparison of rectal vs oral administration and the use of saliva for drug monitoring. British journal of cancer, 1996. 74(1): p. 16-21.

1369. Stray, K., et al., Drug Resistance Assessment Following Administration of Respiratory Syncytial Virus (RSV) Fusion Inhibitor Presatovir to Participants Experimentally Infected With RSV. Journal of Infectious Diseases, 2020. 222(9): p. 1468-1477.

1370. Streich, D.D. and C.H. Swensen, Response to Two Presentations of the Sentence Completion Test of Ego Development. Journal of Personality Assessment, 1985. 49(3): p. 285.

1371. Stroski, M.L., et al., Clinical evaluation of three toothbrush models tested by schoolchildren. International Journal of Dental Hygiene, 2011. 9(2): p. 149-154.

1372. Subudhi, A.W., et al., Combined methazolamide and theophylline improves oxygen saturation but not exercise performance or altitude illness in acute hypobaric hypoxia. Experimental Physiology, 2021. 106(1): p. 117-125.

1373. Sullivan, R., et al., Clinical efficacy of a specifically targeted antimicrobial peptide mouth rinse: targeted elimination of Streptococcus mutans and prevention of demineralization. Caries Res, 2011. 45(5): p. 415-28.

1374. Sun, H.B., W. Zhang, and X.B. Zhou, Risk Factors associated with Early Childhood Caries. Chin J Dent Res, 2017. 20(2): p. 97-104.

1375. Sun, Y.H., et al., A cross-sectional study of health-related behaviors in rural eastern China. Biomedical and Environmental Sciences, 2002. 15(4): p. 347-354.

1376. Sundaram, G., et al., Effect of resin coating on dentine compared to repeated topical applications of fluoride mouthwash after an abrasion and erosion wear regime. Journal of dentistry, 2007. 35(10): p. 814-8.

1377. Sundqvist, M.L., J.O. Lundberg, and E. Weitzberg, Effects of antiseptic mouthwash on resting metabolic rate: A randomized, double-blind, crossover study. Nitric oxide : biology and chemistry, 2016. 61: p. 38-44.

1378. Sung, K.C., et al., Relative bioavailability study of cefuroxime axetil tablets. Journal of Food and Drug Analysis, 1999. 7(1): p. 45-51.

1379. Suter, M.K., et al., Association between work in deforested, compared to forested, areas and human heat strain: an experimental study in a rural tropical environment. Environmental Research Letters, 2019. 14(8).

1380. Suzuki, Y., et al., Influence of Trehalose Mouth Rinse on Anaerobic and Aerobic Exercise Performance. Natural Product Communications, 2020. 15(11).

1381. Suzuki, Y., et al., Pre-exercise Trehalose Ingestion Enhanced Exercise Performance in Male Collegiate Distance Runners. Natural Product Communications, 2020. 15(7): p. 1-6.

1382. Swain, R.A., et al., Do pseudoephedrine or phenylpropanolamine improve maximum oxygen uptake and time to exhaustion? Clinical Journal of Sport Medicine, 1997. 7(3): p. 168-173.

1383. Swaisland, H.C., et al., Exploring the relationship between expression of cytochrome P450 enzymes and gefitinib pharmacokinetics. Clinical Pharmacokinetics, 2006. 45(6): p. 633-644.

1384. Swillen, A., et al., Intelligence and psychosocial adjustment in velocardiofacial syndrome: a study of 37 children and adolescents with VCFS. Journal of medical genetics, 1997. 34(6): p. 453-8.

1385. Swillen, A., et al., Early motor development in young children with 22q.11 deletion syndrome and a conotruncal heart defect. Developmental medicine and child neurology, 2005. 47(12): p. 797-802.

1386. Swillen, A., et al., The Coffin-Siris syndrome: data on mental development, language, behavior and social skills in 12 children. Clinical genetics, 1995. 48(4): p. 177-82.

1387. Swillens, A., et al., Accuracy of carotid strain estimates from ultrasonic wall tracking: a study based on multiphysics simulations and in vivo data. IEEE transactions on medical imaging, 2012. 31(1): p. 131-9.

1388. Swillens, A., et al., Pitfalls of Doppler Measurements for Arterial Blood Flow Quantification in Small Animal Research: A Study Based on Virtual Ultrasound Imaging. Ultrasound in medicine & biology, 2016. 42(6): p. 1399-411.

1389. Swillens, A., et al., Comparison of non-invasive methods for measurement of local pulse wave velocity using FSI-simulations and in vivo data. Annals of biomedical engineering, 2013. 41(7): p. 1567-78.

1390. Szczeklik, A., et al., Safety of a specific COX-2 inhibitor in aspirin-induced asthma. Clinical and Experimental Allergy, 2001. 31(2): p. 219-225.

1391. Szeto, Y.T., et al., Optimized noninvasive procedures to measure DNA damage in comet assay. Human & experimental toxicology, 2012. 31(11): p. 1144-50.

1392. T Celik, A.C., E. Coban, and H.E. Ulker, Effects of mouthwashes on color stability and surface roughness of three different resin-based composites. Nigerian journal of clinical practice, 2021. 24(4): p. 555-560.

1393. t Eijnde, B.O., et al., No effects of oral ribose supplementation on repeated maximal exercise and de novo ATP resynthesis. Journal of Applied Physiology, 2001. 91(5): p. 2275-2281.

1394. Taekema-Roelvink, M.E.J., et al., Pharmacokinetic interaction of solifenacin with an oral contraceptive containing ethinyl estradiol and levonorgestrel in healthy women: A double-blind, placebo-controlled study. Clinical Therapeutics, 2005. 27(9): p. 1403-1410.

1395. Takagi, M., et al., [Evaluation for the training and behavior of dental treatment in our special patients center]. Shigaku = Odontology; journal of Nihon Dental College, 1989. 77(3): p. 910-8.

1396. Takahashi, H., A. Ishida-Yamamoto, and H. Iizuka, Effects of bepotastine, cetirizine, fexofenadine, and olopatadine on histamine-induced wheal-and flare-response, sedation, and psychomotor performance. Clinical and Experimental Dermatology, 2004. 29(5): p. 526-532.

1397. Takahashi, K., [Mycosis in kidney transplant patients]. Nihon Ishinkin Gakkai zasshi = Japanese journal of medical mycology, 2001. 42(4): p. 201-4.

1398. Takatsuka, T., R.A. Exterkate, and J.M. ten Cate, Effects of Isomalt on enamel de- and remineralization, a combined in vitro pH-cycling model and in situ study. Clin Oral Investig, 2008. 12(2): p. 173-7.

1399. Takatsuka, T., R.A.M. Exterkate, and J.M. ten Cate, Effects of Isomalt on enamel de- and remineralization, a combined in vitro pH-cycling model and in situ study. Clinical oral investigations, 2008. 12(2): p. 173-7.

1400. Talent, J.M. and R.W. Gracy, Pilot study of oral polymeric N-acetyl-D-glucosamine as a potential treatment for patients with osteoarthritis. Clinical Therapeutics, 1996. 18(6): p. 1184-1190.

1401. Tan, K.K.C., et al., EFFECT OF DIETARY-FAT ON THE PHARMACOKINETICS AND PHARMACODYNAMICS OF CYCLOSPORINE IN KIDNEY-TRANSPLANT RECIPIENTS. Clinical Pharmacology & Therapeutics, 1995. 57(4): p. 425-433.

1402. Tan, K.S., L.C. McFarlane, and B.J. Lipworth, Concomitant administration of low-dose prednisolone protects against in vivo beta(2)-adrenoceptor subsensitivity induced by regular formoterol. Chest, 1998. 113(1): p. 34-41.

1403. Tanaka, M. and H.C. Margolis, Release of mineral ions in dental plaque following acid production. Arch Oral Biol, 1999. 44(3): p. 253-8.

1404. Tang, K.D., et al., Oral HPV16 Prevalence in Oral Potentially Malignant Disorders and Oral Cavity Cancers. Biomolecules, 2020. 10(2).

1405. Tans, G., et al., A randomized cross-over study on the effects of levonorgestrel- and desogestrel-containing oral contraceptives on the anticoagulant pathways. Thrombosis and Haemostasis, 2000. 84(1): p. 15-21.

1406. Tapia, M., et al., Melatonin Relations With Respiratory Quotient Weaken on Acute Exposure t o High Altitude. Frontiers in Physiology, 2018. 9.

1407. Tarral, A., P. Francheteau, and M. Guerret, Effects of terbinafine on the pharmacokinetics of digoxin in healthy volunteers. Pharmacotherapy, 1997. 17(4): p. 791-795.

1408. Tateishi, T., et al., The different effects of itraconazole on the pharmacokinetics of fexofenadine enantiomers. British Journal of Clinical Pharmacology, 2008. 65(5): p. 693-700.

1409. Taukulis, H.K., et al., Object memory impairment at post-drug Day 15 but not at Day 1 after a regimen of repeated treatment with oral methylphenidate. Neuroscience Letters, 2014. 566: p. 252-256.

1410. Tay, F.R., et al., Potential iatrogenic tetracycline staining of endodontically treated teeth via NaOCl/MTAD irrigation: a preliminary report. J Endod, 2006. 32(4): p. 354-8.

1411. Tay, F.R., K.M. Moulding, and D.H. Pashley, Distribution of nanofillers from a simplified-step adhesive in acid-conditioned dentin. J Adhes Dent, 1999. 1(2): p. 103-17.

1412. Tedesco, T.K., et al., Starch tubing: an alternative method to build up microshear bond test specimens. J Adhes Dent, 2013. 15(4): p. 311-5.

1413. Tehrani, A., et al., Enamel demineralization by mouthrinses containing different concentrations of sucrose. J Dent Res, 1983. 62(12): p. 1216-7.

1414. Tehrani, A., et al., Enamel demineralization and the length of intra-oral exposure to different concentrations of glucose or sucrose. J Dent Res, 1986. 65(2): p. 139-45.

1415. Temyingyong, N., et al., Effect of Short-Course Oral Ciprofloxacin on Isoflavone Pharmacokinetics following Soy Milk Ingestion in Healthy Postmenopausal Women. Evidence-Based Complementary and Alternative Medicine, 2019. 2019.

1416. Tenuta, L.M., et al., Effect of a calcium glycerophosphate fluoride dentifrice formulation on enamel demineralization in situ. Am J Dent, 2009. 22(5): p. 278-82.

1417. Terao, K., et al., Enhancement of oral bioavailability of coenzyme Q(10) by complexation with gamma-cyclodextrin in healthy adults. Nutrition Research, 2006. 26(10): p. 503-508.

1418. Terry, A.V., et al., Protracted effects of chronic oral haloperidol and risperidone on nerve growth factor, cholinergic neurons, and spatial reference learning in rats. Neuroscience, 2007. 150(2): p. 413-424.

1419. Terry, A.V., et al., Time-dependent effects of haloperidol and ziprasidone on nerve growth factor, cholinergic neurons, and spatial learning in rats. Journal of Pharmacology and Experimental Therapeutics, 2006. 318(2): p. 709-724.

1420. Teter, C.J., et al., Relative rectal bioavailability of fluoxetine in normal volunteers. Journal of Clinical Psychopharmacology, 2005. 25(1): p. 74-78.

1421. Tezulas, E., et al., Decontamination of autogenous bone grafts collected during dental implant site preparation: a pilot study. Oral Surg Oral Med Oral Pathol Oral Radiol Endod, 2009. 107(5): p. 656-60.

1422. Thal, L.J., et al., A multicenter double-blind study of controlled-release physostigmine for the treatment of symptoms secondary to Alzheimer's disease. Neurology, 1996. 47(6): p. 1389-1395.

1423. Thebault, J.J., M. Guillaume, and R. Levy, Tolerability, safety, pharmacodynamics, and pharmacokinetics of rasagiline: A potent, selective, and irreversible monoamine oxidase type B inhibitor. Pharmacotherapy, 2004. 24(10): p. 1295-1305.

1424. Thepaut, E., et al., Per- and polyfluoroalkyl substances in serum and associations with food consumption and use of personal care products in the Norwegian biomonitoring study from the EU project EuroMix. Environmental Research, 2021. 195.

1425. Thepaut, E., et al., Per- and polyfluoroalkyl substances in serum and associations with food consumption and use of personal care products in the Norwegian biomonitoring study from the EU project EuroMix. Environmental research, 2021. 195: p. 110795.

1426. Thompson, D.G., et al., Extra intestinal influences on exhaled breath hydrogen measurements during the investigation of gastrointestinal disease. Gut, 1985. 26(12): p. 1349-52.

1427. Tillman, E.M. and M. Opilla, Considerations for Fueling an Endurance Athlete With Home Parenteral Nutrition. Nutrition in Clinical Practice, 2017. 32(6): p. 782-788.

1428. Timan, P., et al., Effect of synbiotic fermented milk on oral bioavailability of isoflavones in postmenopausal women. International Journal of Food Sciences and Nutrition, 2014. 65(6): p. 761-767.

1429. Timmons, B.W., M.J. Hamadeh, and M.A. Tarnopolsky, No effect of short-term 17 beta-estradiol supplementation in healthy men on systemic inflammatory responses to exercise. American Journal of Physiology-Regulatory Integrative and Comparative Physiology, 2006. 291(2): p. R285-R290.

1430. Tjandrawinata, R.R., et al., Single dose pharmacokinetic equivalence study of two gabapentin preparations in healthy subjects. Drug Design Development and Therapy, 2014. 8: p. 1249-1255.

1431. Tobias, G. and A.B. Spanier, Developing a Mobile App (iGAM) to Promote Gingival Health by Professional Monitoring of Dental Selfies: User-Centered Design Approach. JMIR mHealth and uHealth, 2020. 8(8): p. e19433.

1432. Tobin, L., L. Simonsen, and J. Bulow, Real-time contrast-enhanced ultrasound determination of microvascular blood volume in abdominal subcutaneous adipose tissue in man. Evidence for adipose tissue capillary recruitment. Clinical Physiology and Functional Imaging, 2010. 30(6): p. 447-452.

1433. Tomko, P.M., et al., Mouth Rinsing Cabohydrates Serially does not Improve Repeated Sprint Time. Journal of Human Kinetics, 2019. 67(1): p. 133-142.

1434. Tonacchera, M., et al., Functional characteristics of three new germline mutations of the thyrotropin receptor gene causing autosomal dominant toxic thyroid hyperplasia. The Journal of clinical endocrinology and metabolism, 1996. 81(2): p. 547-54.

1435. Tonzetich, J., Production and origin of oral malodor: a review of mechanisms and methods of analysis. J Periodontol, 1977. 48(1): p. 13-20.

1436. Toothaker, R.D., et al., Absence of pharmacokinetic interaction between orally co-administered naproxen sodium and diphenhydramine hydrochloride. Biopharmaceutics & Drug Disposition, 2000. 21(6): p. 229-233.

1437. Topcuoglu, N., et al., Antibacterial effect of Kenger gum on mutans streptococci and its cytotoxic effect on the 3T3 fibroblast cell line. Oral Health Prev Dent, 2015. 13(2): p. 157-62.

1438. Topuz, E., et al., Effect of oral administration of kefir on serum proinflammatory cytokines on 5-FU induced oral mucositis in patients with colorectal cancer. Investigational new drugs, 2008. 26(6): p. 567-72.

1439. Tournaye, H., et al., EFFECT OF PENTOXIFYLLINE ON IMPLANTATION AND POSTIMPLANTATION DEVELOPMENT OF MOUSE EMBRYOS IN-VITRO. Human Reproduction, 1993. 8(11): p. 1948-1954.

1440. Trachet, B., et al., Performance comparison of ultrasound-based methods to assess aortic diameter and stiffness in normal and aneurysmal mice. PloS one, 2015. 10(5): p. e0129007.

1441. Trindade, T.F., et al., Bonding Effectiveness of Universal Adhesive to Intracoronal Bleached Dentin Treated with Sodium Ascorbate. Braz Dent J, 2016. 27(3): p. 303-8.

1442. Trommelen, J., et al., A Sucrose Mouth Rinse Does Not Improve 1-hr Cycle Time Trial Performance When Performed in the Fasted or Fed State. Int J Sport Nutr Exerc Metab, 2015. 25(6): p. 576-83.

1443. Trommelen, J., et al., A Sucrose Mouth Rinse Does Not Improve 1-hr Cycle Time Trial Performance When Performed in the Fasted or Fed State. International journal of sport nutrition and exercise metabolism, 2015. 25(6): p. 576-83.

1444. Tsai, T.W., et al., Effect of green tea extract supplementation on glycogen replenishment in exercised human skeletal muscle. British Journal of Nutrition, 2017. 117(10): p. 1343-1350.

1445. Tsang, C.S.P., et al., Phospholipase, proteinase and haemolytic activities of Candida albicans isolated from oral cavities of patients with type 2 diabetes mellitus. J Med Microbiol, 2007. 56(Pt 10): p. 1393-1398.

1446. Tsao, P.S., et al., L-ARGININE ATTENUATES PLATELET REACTIVITY IN HYPERCHOLESTEROLEMIC RABBITS. Arteriosclerosis and Thrombosis, 1994. 14(10): p. 1529-1533.

1447. Tsuchiya, H., T. Miyazaki, and S. Ohmoto, High-performance liquid chromatographic analysis of chlorhexidine in saliva after mouthrinsing. Caries research, 1999. 33(2): p. 156-63.

1448. Tsuruta, S., et al., Inhibition of nifedipine metabolism in dogs by erythromycin: Difference between the gut wall and the liver. Journal of Pharmacy and Pharmacology, 1997. 49(12): p. 1205-1210.

1449. Tuck, C.J., et al., Increasing Symptoms in Irritable Bowel Symptoms With Ingestion of Galacto-Oligosaccharides Are Mitigated by alpha-Galactosidase Treatment. American Journal of Gastroenterology, 2018. 113(1): p. 124-134.

1450. Turner, C.E., et al., Carbohydrate in the mouth enhances activation of brain circuitry involved in motor performance and sensory perception. Appetite, 2014. 80: p. 212-9.

1451. Turner, C.E., B.R. Russell, and N. Gant, Comparative quantification of dietary supplemented neural creatine concentrations with H-1-MRS peak fitting and basis spectrum methods. Magnetic Resonance Imaging, 2015. 33(9): p. 1163-1167.

1452. Turssi, C.P., et al., Counteractive effect of antacid suspensions on intrinsic dental erosion. European Journal of Oral Sciences, 2012. 120(4): p. 349-352.

1453. Turssi, C.P., et al., Counteractive effect of antacid suspensions on intrinsic dental erosion. European journal of oral sciences, 2012. 120(4): p. 349-52.

1454. Tüzüner, T., et al., Direct and transdentinal (indirect) antibacterial activity of commercially available dental gel formulations against Streptococcus mutans. Med Princ Pract, 2013. 22(4): p. 397-401.

1455. Tworoger, S.S., et al., Effect of a nighttime magnetic field exposure on sleep patterns in young women. American Journal of Epidemiology, 2004. 160(3): p. 224-229.

1456. Uhler, M.L., et al., The effect of nonsteroidal antiinflammatory drugs on ovulation: a prospective, randomized clinical trial. Fertility and Sterility, 2001. 76(5): p. 957-961.

1457. Ullah, A., et al., Bioequivalence Evaluation of Two Capsule Formulations of Amoxicillin in Healthy Adult Male Bangladeshi Volunteers: A Single-Dose, Randomized, Open-Label, Two-Period Crossover Study. Current Therapeutic Research-Clinical and Experimental, 2008. 69(6): p. 504-513.

1458. Umetsu, H., et al., Association between glucan synthesis by streptococcus mutans and caries incidence in schoolchildren receiving a fluoride mouth rinse. Oral Health Prev Dent, 2012. 10(2): p. 161-6.

1459. Uslu, A., et al., FASTING AND FED BIOEQUIVALENCE STUDIES OF ETODOLAC SUSTAINED RELEASE TABLETS MANUFACTURED IN TURKEY IN HEALTHY VOLUNTEERS. Nobel Medicus, 2016. 12(2): p. 53-59.

1460. Utreja, D., A. Tewari, and H.S. Chawla, A study of influence of sugars on the modulations of dental plaque pH in children with rampant caries, moderate caries and no caries. J Indian Soc Pedod Prev Dent, 2010. 28(4): p. 278-81.

1461. Vaahtoniemi, L.H., Surface ultrastructure of intact and in situ chlorhexidine-treated human buccal cells. A method for scanning electron microscopy. Acta odontologica Scandinavica, 1997. 55(5): p. 277-81.

1462. Vaahtoniemi, L.H., Surface ultrastructure of intact and in situ chlorhexidine-treated human buccal cells - A method for scanning electron microscopy. Acta Odontologica Scandinavica, 1997. 55(5): p. 277-281.

1463. Vacca Smith, A.M. and W.H. Bowen, In situ studies of pellicle formation on hydroxyapatite discs. Arch Oral Biol, 2000. 45(4): p. 277-91.

1464. Vacharotayangul, P., et al., Higher prevalence of oral human papillomavirus infection in HIV-positive than HIV-negative Thai men and women. Cancer epidemiology, 2015. 39(6): p. 917-22.

1465. Vachharajani, N.N., W.C. Shyu, and R.H. Barbhaiya, Pharmacokinetic interaction between butorphanol nasal spray and oral metoclopramide in healthy women. Journal of Clinical Pharmacology, 1997. 37(10): p. 979-985.

1466. Vachharajani, N.N., et al., Lack of effect of food on the oral bioavailability of irbesartan in healthy male volunteers. Journal of Clinical Pharmacology, 1998. 38(5): p. 433-436.

1467. Van Aken, K., et al., The motor profile of primary school-age children with a 22q11.2 deletion syndrome (22q11.2DS) and an age- and IQ-matched control group. Child neuropsychology : a journal on normal and abnormal development in childhood and adolescence, 2009. 15(6): p. 532-42.

1468. Van Aken, K., et al., Motor development in school-aged children with 22q11 deletion (velocardiofacial/DiGeorge syndrome). Developmental medicine and child neurology, 2007. 49(3): p. 210-3.

1469. Van Campenhout, S., et al., Microduplication 22q11.2: a description of the clinical, developmental and behavioral characteristics during childhood. Genetic counseling (Geneva, Switzerland), 2012. 23(2): p. 135-48.

1470. Van Cutsem, J., et al., A caffeine-maltodextrin mouth rinse counters mental fatigue. Psychopharmacology, 2018. 235(4): p. 947-958.

1471. van den Besselaar, A., et al., Preparation of Control Blood for External Quality Assessment of Point-of-Care International Normalized Ratio Testing in the Netherlands. American Journal of Clinical Pathology, 2014. 141(6): p. 878-883.

1472. Van Den Heuvel, E., et al., Developmental trajectories of structural and pragmatic language skills in school-aged children with Williams syndrome. Journal of intellectual disability research : JIDR, 2016. 60(10): p. 903-19.

1473. Van Den Heuvel, E., et al., Atypical language characteristics and trajectories in children with 22q11.2 deletion syndrome. Journal of communication disorders, 2018. 75: p. 37-56.

1474. Van Den Heuvel, E., et al., Referential communication abilities in children with 22q11.2 deletion syndrome. International journal of speech-language pathology, 2017. 19(5): p. 490-502.

1475. van der Mei, H.C., et al., Bacterial detachment from salivary conditioning films by dentifrice supernates. J Clin Dent, 2002. 13(1): p. 44-9.

1476. Van der Sluijs, E., et al., Prebrushing rinse with water on plaque removal: a split-mouth design. International Journal of Dental Hygiene, 2017. 15(4): p. 345-351.

1477. Van der Weijden, G.A., et al., Comparison of different approaches to assess bleeding on probing as indicators of gingivitis. Journal of clinical periodontology, 1994. 21(9): p. 589-94.

1478. van der Weijden, G.A., et al., A clinical comparison of three powered toothbrushes. Journal of clinical periodontology, 2002. 29(11): p. 1042-7.

1479. Van Dyke, T., et al., Clinical and microbial evaluation of a histatin-containing mouthrinse in humans with experimental gingivitis: a phase-2 multi-center study. J Clin Periodontol, 2002. 29(2): p. 168-76.

1480. Van Houte, J., J. Russo, and K.S. Prostak, Increased pH-lowering ability of Streptococcus mutans cell masses associated with extracellular glucan-rich matrix material and the mechanisms involved. J Dent Res, 1989. 68(3): p. 451-9.

1481. Van Leeuwen, M., et al., Effectiveness of various interventions on maintenance of gingival health during 1 year - a randomized clinical trial. Int J Dent Hyg, 2017. 15(4): p. e16-e27.

1482. van Maanen, R. and D. Bentley, Bioequivalence of Zonisamide Orally Dispersible Tablet and Immediate-Release Capsule Formulations: Results From Two Open-Label, Randomized-Sequence, Single-Dose, Two-Period, Two-Treatment Crossover Studies in Healthy Male Volunteers. Clinical Therapeutics, 2009. 31(6): p. 1244-1255.

1483. van Strijp, A.J., et al., Chlorhexidine efficacy in preventing lesion formation in enamel and dentine: an in situ study. Caries Res, 2008. 42(6): p. 460-5.

1484. Van Strydonck, D.A.C., et al., The anti-plaque efficacy of a chlorhexidine mouthrinse used in combination with toothbrushing with dentifrice. Journal of clinical periodontology, 2004. 31(8): p. 691-5.

1485. Vandermolen, D.T., et al., Metformin increases the ovulatory rate and pregnancy rate from clomiphene citrate in patients with polycystic ovary syndrome who are resistant to clomiphene citrate alone. Fertility and Sterility, 2001. 75(2): p. 310-315.

1486. Verdonck-de Leeuw, I.M., et al., Advancing interdisciplinary research in head and neck cancer through a multicenter longitudinal prospective cohort study: the NETherlands QUality of life and BIomedical Cohort (NET-QUBIC) data warehouse and biobank. BMC cancer, 2019. 19(1): p. 765.

1487. Veronese, M.L., et al., Exposure-dependent inhibition of intestinal and hepatic CYP3A4 in vivo by grapefruit juice. Journal of Clinical Pharmacology, 2003. 43(8): p. 831-839.

1488. Victor J, B.-S., J. Prestes, and A.A.R. Geraldes, EFFECT OF CARBOHYDRATE MOUTH RINSE ON TRAINING LOAD VOLUME IN RESISTANCE EXERCISES. Journal of Strength & Conditioning Research, 2019. 33(6): p. 1653-1657.

1489. Vilasan, A., et al., Comparative Evaluation of Chitosan Chlorhexidine Mouthwash in Plaque Control: A Preliminary Randomized Controlled Clinical Trial. J Int Acad Periodontol, 2020. 22(3): p. 166-173.

1490. Vissink, A., A. Visser, and F.K. Spijkervet, [Oral medicine 2. Treatment of dry mouth]. Ned Tijdschr Tandheelkd, 2012. 119(11): p. 555-60.

1491. Vitale, K. and A. Getzin, Nutrition and Supplement Update for the Endurance Athlete: Review and Recommendations. Nutrients, 2019. 11(6).

1492. Vivas, D., et al., Impact of Intravenous Lysine Acetylsalicylate Versus Oral Aspirin on Prasugrel-Inhibited Platelets Results of a Prospective, Randomized, Crossover Study (the ECCLIPSE Trial). Circulation-Cardiovascular Interventions, 2015. 8(5).

1493. Vivek, H.P., et al., Effect of Mouthrinses containing Olive Oil, Fluoride, and Their Combination on Enamel Erosion: An in vitro Study. The journal of contemporary dental practice, 2018. 19(2): p. 130-136.

1494. Vladareanu, R., et al., New evidence on oral L. plantarum P17630 product in women with history of recurrent vulvovaginal candidiasis (RVVC): a randomized double-blind placebo-controlled study. European Review for Medical and Pharmacological Sciences, 2018. 22(1): p. 262-267.

1495. Vojdani, A., et al., New evidence for antioxidant properties of vitamin C. Cancer Detection and Prevention, 2000. 24(6): p. 508-523.

1496. Vokurka, S., et al., The comparative effects of povidone-iodine and normal saline mouthwashes on oral mucositis in patients after high-dose chemotherapy and APBSCT--results of a randomized multicentre study. Support Care Cancer, 2005. 13(7): p. 554-8.

1497. Vuletić, L., et al., The rise in glucose concentration in saliva samples mixed with test foods monitored using a glucometer: An observational pilot study. J Oral Biosci, 2019. 61(4): p. 201-206.

1498. Wadazumi, T., et al., Glucose Mouth Rinse And Mouth Spray Enhance Prolonged Exercise Performance In Healthy Male College Students. Medicine and Science in Sports and Exercise, 2020. 52(17): p. 592-592.

1499. Wagner, H., et al., Biomechanical muscle properties and angiotensin-converting enzyme gene polymorphism: a model-based study. European journal of applied physiology, 2006. 98(5): p. 507-15.

1500. Waldock, K.A.M., et al., The elderly's physiological and perceptual responses to cooling during simulated activities of daily living in UK summer climatic conditions. Public health, 2021. 193: p. 1-9.

1501. Walsh, T., et al., Clinical assessment to screen for the detection of oral cavity cancer and potentially malignant disorders in apparently healthy adults. Cochrane Database of Systematic Reviews, 2013(11).

1502. Walsh, T.F., et al., The effect of irrigation with chlorhexidine or saline on plaque vitality. Journal of clinical periodontology, 1995. 22(3): p. 262-4.

1503. Wang, J.Q., et al., Bioavailability of Edaravone Sublingual Tablet Versus Intravenous Infusion in Healthy Male Volunteers. Clinical Therapeutics, 2018. 40(10): p. 1683-1691.

1504. Wang, L., et al., Pharmacokinetics and Bioequivalence Evaluation of Erlotinib Hydrochloride Tablets: Randomized, Open-Label, 2-Period Crossover Study in Healthy Chinese Subjects. Clinical Pharmacology in Drug Development, 2021. 10(2): p. 166-172.

1505. Wang, P., et al., Association between Polymorphisms of Vitamin D Receptor Gene ApaI, BsmI and TaqI and Muscular Strength in Young Chinese Women. International Journal of Sports Medicine, 2006. 27(3): p. 182-186.

1506. Wang, X.T., et al., Resource forecasting: Differential effects of glucose taste and ingestion on delay discounting and self-control. Appetite, 2018. 121: p. 101-110.

1507. Wara, A., et al., Short-Term Estrogen Replacement Effects on Insulin Sensitivity and Glucose Tolerance in At-Risk Cats for Feline Diabetes Mellitus. Plos One, 2015. 10(6).

1508. Washif, J.-A. and C.M. Beaven, Effects Of A Caffeine-carbohydrate Mouth Rinsing On Sprinting Kinetics And Kinematics In Fasted Athletes: 3211 Board #80 June 2 8:00 AM - 9:30 AM. Medicine & Science in Sports & Exercise, 2018. 50: p. 791-791.

1509. Washif, J.A. and C.M. Beaven, Effects Of A Caffeine-carbohydrate Mouth Rinsing On Sprinting Kinetics And Kinematics In Fasted Athletes. Medicine and Science in Sports and Exercise, 2018. 50(5): p. 791-791.

1510. Waśko-Grabowska, A., et al., Efficiency of supersaturated calcium phosphate mouth rinse treatment in patients receiving high-dose melphalan or BEAM prior to autologous blood stem cell transplantation: a single-center experience. Transplant Proc, 2011. 43(8): p. 3111-3.

1511. Watson, P., D. Nichols, and P. Cordery, Mouth rinsing with a carbohydrate solution does not influence cycle time trial performance in the heat. Applied physiology, nutrition, and metabolism = Physiologie appliquee, nutrition et metabolisme, 2014. 39(9): p. 1064-9.

1512. Watson, P., D. Nichols, and P. Cordery, Mouth rinsing with a carbohydrate solution does not influence cycle time trial performance in the heat1. Applied Physiology, Nutrition & Metabolism, 2014. 39(9): p. 1064-1069.

1513. Weatherell, J.A., et al., Site-specific differences in human dental plaque pH after sucrose rinsing. Arch Oral Biol, 1988. 33(12): p. 871-3.

1514. Webb, J., et al., Levels of 1-hydroxypyrene in urine of people living in an oil producing region of the Andean Amazon (Ecuador and Peru). International Archives of Occupational and Environmental Health, 2018. 91(1): p. 105-115.

1515. Webster, C.C., et al., A Carbohydrate Ingestion Intervention in an Elite Athlete Who Follows a Low-Carbohydrate High-Fat Diet. International Journal of Sports Physiology and Performance, 2018. 13(7): p. 957-960.

1516. Wegehaupt, F.J., et al., Influence of extra- and intra-oral application of CPP-ACP and fluoride on re-hardening of eroded enamel. Acta odontologica Scandinavica, 2012. 70(3): p. 177-83.

1517. Wegienka, G. and D.D. Baird, Potential bias due to excluding oral contraceptive users when estimating menstrual cycle characteristics. American Journal of Epidemiology, 2003. 158(10): p. 947-950.

1518. Wei, C.Y., et al., Deep Ocean Mineral Supplementation Enhances the Cerebral Hemodynamic Response during Exercise and Decreases Inflammation Postexercisein Men at Two Age Levels. Frontiers in Physiology, 2017. 8.

1519. Wennerholm, K. and C.G. Emilson, Sucrose retention and colonization by mutans streptococci at different sites of the dentition. Caries Res, 1995. 29(5): p. 396-401.

1520. Westhoff, C.L., et al., Clotting factor changes during the first cycle of oral contraceptive use. Contraception, 2016. 93(1): p. 70-76.

1521. Westhoff, C.L., et al., Estimating systemic exposure to ethinyl estradiol from an oral contraceptive. American Journal of Obstetrics and Gynecology, 2015. 212(5).

1522. Westphal, K., et al., Oral bioavailability of digoxin is enhanced by talinolol: Evidence for involvement of intestinal P-glycoprotein. Clinical Pharmacology & Therapeutics, 2000. 68(1): p. 6-12.

1523. Weyl, K.G. and R.M. Dougherty, Contact transmission of avian leukosis virus. Journal of the National Cancer Institute, 1977. 58(4): p. 1019-25.

1524. Whelan, G.J., et al., Effect of montelukast on time-course of exhaled nitric oxide in asthma: Influence of LTC4 synthase A(-444)C polymorphism. Pediatric Pulmonology, 2003. 36(5): p. 413-420.

1525. Whinton, A.K., et al., Repeated Application of a Novel Creatine Cream Improves Muscular Peak and Average Power in Male Subjects. Journal of Strength and Conditioning Research, 2020. 34(9): p. 2482-2491.

1526. Whitham, M. and J. McKinney, Effect of a carbohydrate mouthwash on running time-trial performance. Journal of sports sciences, 2007. 25(12): p. 1385-92.

1527. Wickham, K.A. and L.L. Spriet, Administration of Caffeine in Alternate Forms. Sports medicine (Auckland, N.Z.), 2018. 48(Suppl 1): p. 79-91.

1528. Wiesinger, H., et al., Bioequivalence Evaluation of a Folate-Supplemented Oral Contraceptive Containing Ethinylestradioli Drospirenone/Levomefolate Calcium versus Ethinylestradiol/Drospirenone and Levomefolate Calcium Alone. Clinical Drug Investigation, 2012. 32(10): p. 673-684.

1529. Willaert, A., et al., Vestibular dysfunction is a manifestation of 22q11.2 deletion syndrome. American journal of medical genetics. Part A, 2019. 179(3): p. 448-454.

1530. Willard, S., et al., The effects of GnRH administration postinsemination on serum concentrations of progesterone and pregnancy rates in dairy cattle exposed to mild summer heat stress. Theriogenology, 2003. 59(8): p. 1799-810.

1531. Willard, S.T., et al., Administration of 6-methoxybenzoxazolinone (MBOA) does not augment ovulatory responses in St. Croix White ewes superovulated with PMSG. Animal reproduction science, 2006. 93(3-4): p. 280-91.

1532. Williams, P.D., et al., Symptom monitoring and dependent care during cancer treatment in children: pilot study. Cancer nursing, 2006. 29(3): p. 188-97.

1533. Williams, S.A., et al., Plasma protein patterns as comprehensive indicators of health. Nature medicine, 2019. 25(12): p. 1851-1857.

1534. Williams, S.C., et al., Modafinil effects during acute continuous positive airway pressure withdrawal: a randomized crossover double-blind placebo-controlled trial. American journal of respiratory and critical care medicine, 2010. 181(8): p. 825-31.

1535. Williams, S.C., et al., The effect of modafinil following acute CPAP withdrawal: a preliminary study. Sleep & breathing = Schlaf & Atmung, 2008. 12(4): p. 359-64.

1536. Williams, S.N. and A.S. Undieh, Dopamine-sensitive signaling mediators modulate psychostimulant-induced ultrasonic vocalization behavior in rats. Behavioural brain research, 2016. 296: p. 1-6.

1537. Williamson, S., et al., The patient needs assessment in cancer care: identifying barriers and facilitators to implementation in the UK and Canada. Supportive care in cancer : official journal of the Multinational Association of Supportive Care in Cancer, 2021. 29(2): p. 805-812.

1538. Williamson, S.K., et al., A phase I study of intraperitoneal nanoparticulate paclitaxel (Nanotax R) in patients with peritoneal malignancies. Cancer chemotherapy and pharmacology, 2015. 75(5): p. 1075-87.

1539. Williamson, S.K., et al., Phase II trial of gemcitabine plus irinotecan in patients with esophageal cancer: a Southwest Oncology Group (SWOG) trial. American journal of clinical oncology, 2006. 29(2): p. 116-22.

1540. Williamson, S.K., et al., Phase II evaluation of sorafenib in advanced and metastatic squamous cell carcinoma of the head and neck: Southwest Oncology Group Study S0420. Journal of clinical oncology : official journal of the American Society of Clinical Oncology, 2010. 28(20): p. 3330-5.

1541. Willmott, A.P. and J. Dapena, The planarity of the stickface motion in the field hockey hit. Journal of sports sciences, 2012. 30(4): p. 369-77.

1542. Wilson, N., et al., Licence to swill: James Bond's drinking over six decades. The Medical journal of Australia, 2018. 209(11): p. 495-500.

1543. Wilson, P.B., Does Carbohydrate Intake During Endurance Running Improve Performance? A Critical Review. Journal of strength and conditioning research, 2016. 30(12): p. 3539-3559.

1544. Wilson, S., Running with rugby. No longer just for bloodied, beer-swilling louts, rugby is finding new popularity among American students. In response, several schools are turning their club teams into varsity sports. Athletic Management, 2002. 14(5): p. 45-46;48;50-51;53.

1545. Wilson, S.D., et al., A Correlational Study on Taste Alterations and Quality of Life Among Cancer Patients. Journal of Health and Allied Sciences Nu.

1546. Winkel, E.G., et al., Clinical effects of a new mouthrinse containing chlorhexidine, cetylpyridinium chloride and zinc-lactate on oral halitosis. A dual-center, double-blind placebo-controlled study. Journal of clinical periodontology, 2003. 30(4): p. 300-6.

1547. Winkelmann, B.R., et al., DISCORDANCE OF ANTIISCHEMIC AND HEMODYNAMIC-EFFECTS OF CAPTOPRIL IN STABLE CORONARY-ARTERY DISEASE. Coronary Artery Disease, 1994. 5(10): p. 829-844.

1548. Wise, P.M., P.A. Breslin, and P. Dalton, Sweet taste and menthol increase cough reflex thresholds. Pulm Pharmacol Ther, 2012. 25(3): p. 236-41.

1549. Woessner, M., et al., A stepwise reduction in plasma and salivary nitrite with increasing strengths of mouthwash following a dietary nitrate load. Nitric oxide : biology and chemistry, 2016. 54: p. 1-7.

1550. Woessner, M.N., et al., Effect of inorganic nitrate on exercise capacity, mitochondria respiration, and vascular function in heart failure with reduced ejection fraction. Journal of Applied Physiology, 2020. 128(5): p. 1355-1364.

1551. Wolf, O.T., et al., Effects of a two-week physiological dehydroepiandrosterone substitution on cognitive performance and well-being in healthy elderly women and men. Journal of Clinical Endocrinology & Metabolism, 1997. 82(7): p. 2363-2367.

1552. Wood, C.E., et al., Effects of estradiol with micronized progesterone or medroxyprogesterone acetate on risk markers for breast cancer in postmenopausal monkeys. Breast Cancer Research and Treatment, 2007. 101(2): p. 125-134.

1553. Wright, B.F. and G. Davison, Carbohydrate Mouth Rinse Improves 1.5 h Run Performance: Is There A Dose-Effect? International Journal of Exercise Science, 2013. 6(4): p. 328-340.

1554. Wright, D.G., A.I. Meierovics, and J.M. Foxley, Assessing the delivery of neutrophils to tissues in neutropenia. Blood, 1986. 67(4): p. 1023-30.

1555. Wring, S., et al., Clinical Pharmacokinetics and Drug-Drug Interaction Potential for Coadministered SCY-078, an Oral Fungicidal Glucan Synthase Inhibitor, and Tacrolimus. Clinical Pharmacology in Drug Development, 2019. 8(1): p. 60-69.

1556. Wu, G.L., et al., Pharmacokinetics and safety of sitafloxacin after single oral doses in healthy volunteers. International Journal of Clinical Pharmacology and Therapeutics, 2014. 52(12): p. 1037-1044.

1557. Wu, G.L., et al., Safety and pharmacokinetics of dicloxacillin in healthy Chinese volunteers following single and multiple oral doses. Drug Design Development and Therapy, 2015. 9: p. 5687-5695.

1558. Wyganowska-Swiatkowska, M., et al., Effects of chlorhexidine, essential oils and herbal medicines (Salvia, Chamomile, Calendula) on human fibroblast in vitro. Cent Eur J Immunol, 2016. 41(2): p. 125-31.

1559. Wymenga, A.N., et al., Phase I study of transforming growth factor-beta3 mouthwashes for prevention of chemotherapy-induced mucositis. Clinical cancer research : an official journal of the American Association for Cancer Research, 1999. 5(6): p. 1363-8.

1560. Wymenga, A.N., et al., A new in vitro assay for quantitation of chemotherapy-induced mucositis. British journal of cancer, 1997. 76(8): p. 1062-6.

1561. Wymenga, A.N.M., et al., Phase I study of transforming growth factor-beta 3 mouthwashes for prevention of chemotherapy-induced mucositis. Clinical Cancer Research, 1999. 5(6): p. 1363-1368.

1562. Xiao, J.L., et al., Oral Prevalence of Candida Species in Patients Undergoing Systemic Glucocorticoid Therapy and the Antifungal Sensitivity of the Isolates. Infection and Drug Resistance, 2020. 13: p. 2601-2607.

1563. Xu, F.G., et al., Pharmacokinetics and Bioequivalence Study of Two Cetirizine Hydrochloride Formulations in Healthy Chinese Male Volunteers. Arzneimittelforschung-Drug Research, 2009. 59(9): p. 440-444.

1564. Xu, F.G., et al., Bioequivalence assessment of two formulations of spironolactone in Chinese healthy male volunteers. Arzneimittel-Forschung-Drug Research, 2008. 58(3): p. 117-121.

1565. Yaegaki, K., et al., Standardization of clinical protocols in oral malodor research. Journal of Breath Research, 2012. 6(1).

1566. Yaegaki, K., et al., Tongue brushing and mouth rinsing as basic treatment measures for halitosis. Int Dent J, 2002. 52 Suppl 3: p. 192-6.

1567. Yamamoto, T. and Y. Kawamura, [Studies on a water "rinse effect" after sucrose application to the tongue of the rat]. Nihon Seirigaku Zasshi, 1971. 33(5): p. 294-302.

1568. Yan, J.H., et al., Absolute bioavailability and stereoselective pharmacokinetics of doxepin. Xenobiotica, 2002. 32(7): p. 615-623.

1569. Yan, K.Y., et al., Bioequivalence Analysis of 2 Dapoxetine Hydrochloride Formulations in Healthy Chinese Male Volunteers Under Fed and Fasting Conditions: A Randomized, Open-Label, 2-Sequence, 2-Period, 2-Way Crossover Study. Clinical Pharmacology in Drug Development, 2021. 10(4): p. 384-392.

1570. Yan, Y.J., et al., Band-Selection of a Portal LED-Induced Autofluorescence Multispectral Imager to Improve Oral Cancer Detection. Sensors, 2021. 21(9).

1571. Yang, M.S., et al., In vivo evaluation of two novel controlled-release nitrendipine formulations. Drug Development and Industrial Pharmacy, 2005. 31(7): p. 589-595.

1572. Yap, A.U.J., et al., Effect of mouthrinses on microhardness and wear of composite and compomer restoratives. Operative dentistry, 2003. 28(6): p. 740-6.

1573. Yerino, G.A., et al., Bioequivalence study of two oral tablet formulations containing saquinavir mesylate boosted with ritonavir in healthy male subjects. Arzneimittel-Forschung-Drug Research, 2011. 61(8): p. 481-487.

1574. Yong, C.L., V.C. Dias, and J. Stangier, Multiple-dose pharmacokinetics of telmisartan and of hydrochlorothiazide following concurrent administration in healthy subjects. Journal of Clinical Pharmacology, 2000. 40(12): p. 1323-1330.

1575. Yovel, G., K. Shakhar, and S. Ben-Eliyahu, The effects of sex, menstrual cycle, and oral contraceptives on the number and activity of natural killer cells. Gynecologic Oncology, 2001. 81(2): p. 254-262.

1576. Yu, D., et al., Effect of fluoride/essential oils-containing mouthrinse on the microhardness of demineralized bovine enamel. Am J Dent, 2004. 17(3): p. 216-8.

1577. Yu, J., et al., Bioequivalence and Comparison of Pharmacokinetic Properties of 4-mg Tablet Formulations of Rosiglitazone Hydrochloride and Rosiglitazone Maleate: A Single-Dose, Randomized, Open-Label, Two-Period Crossover Study in Healthy Adult Male Chinese Volunteers. Clinical Therapeutics, 2008. 30(12): p. 2272-2279.

1578. Yurtseven, N. and S. Gökalp, Oral sugar clearance and other caries-related factors of stimulated whole saliva in patients with secondary Sjögren syndrome. Quintessence Int, 2007. 38(3): p. e151-7.

1579. Zaid, A.N., et al., Investigation of the bioequivalence of montelukast chewable tablets after a single oral administration using a validated LC-MS/MS method. Drug Design Development and Therapy, 2015. 9: p. 5315-5321.

1580. Zanconato, S., et al., CO2-C-13 WASHOUT DYNAMICS DURING INTERMITTENT EXERCISE IN CHILDREN AND ADULTS. Journal of Applied Physiology, 1992. 73(6): p. 2476-2482.

1581. Zaura, E. and S. Twetman, Critical Appraisal of Oral Pre- and Probiotics for Caries Prevention and Care. Caries Res, 2019. 53(5): p. 514-526.

1582. Zaura, E., C. van Loveren, and J.M. ten Cate, Efficacy of fluoride toothpaste in preventing demineralization of smooth dentin surfaces and narrow grooves in situ under frequent exposures to sucrose or bananas. Caries Res, 2005. 39(2): p. 116-22.

1583. Zaura, E., C. van Loveren, and J.M. ten Cate, [Dentin demineralization in situ during frequent exposures to bananas or sucrose]. Ned Tijdschr Tandheelkd, 2006. 113(9): p. 351-5.

1584. Zee, K., J. Rundegren, and R. Attstrom, Effect of delmopinol hydrochloride mouthrinse on plaque formation and gingivitis in "rapid" and "slow" plaque formers. Journal of clinical periodontology, 1997. 24(7): p. 486-91.

1585. Zeng, Q.H., et al., Effect of alcohol stimulation on salivary pellicle formation on human tooth enamel surface and its lubricating performance. Journal of the Mechanical Behavior of Biomedical Materials, 2017. 75: p. 567-573.

1586. Zero, D.T., J. van Houte, and J. Russo, The intra-oral effect on enamel demineralization of extracellular matrix material synthesized from sucrose by Streptococcus mutans. J Dent Res, 1986. 65(6): p. 918-23.

1587. Zettl, F., et al., Age-dependent increase of treatment-related mortality in older patients with aggressive B cell lymphoma: analysis of outcome, treatment feasibility, and toxicity in 1171 elderly patients with aggressive B cell lymphoma-data from phase II and III trials of the DSHNHL (German High-Grade Non-Hodgkin's Lymphoma Study Group). Annals of hematology, 2021. 100(4): p. 1031-1038.

1588. Zhang, J. and S. Kashket, Inhibition of salivary amylase by black and green teas and their effects on the intraoral hydrolysis of starch. Caries Res, 1998. 32(3): p. 233-8.

1589. Zhao, Y., et al., Variance of IQ is partially dependent on deletion type among 1,427 22q11.2 deletion syndrome subjects. American journal of medical genetics. Part A, 2018. 176(10): p. 2172-2181.

1590. Zheng, L., et al., Bioequivalence study of two mirtazapine oral tablet formulations in healthy Chinese male volunteers. International Journal of Clinical Pharmacology and Therapeutics, 2012. 50(5): p. 368-374.

1591. Zhou, H.L., et al., Pharmacokinetic Properties and Tolerability of Cycloserine Following Oral Administration in Healthy Chinese Volunteers: A Randomized, Open-Label, Single- and Multiple-Dose 3-Way Crossover Study. Clinical Therapeutics, 2015. 37(6): p. 1292-1300.

1592. Zhou, S., et al., Muscle and plasma coenzyme Q(10) concentration, aerobic power and exercise economy of healthy men in response to four weeks of supplementation. Journal of Sports Medicine and Physical Fitness, 2005. 45(3): p. 337-346.

1593. Zhu, Y., P. Statkevich, and D.L. Cutler, Effect of food on the pharmacokinetics of lonafarnib (SCH 66336) following single and multiple doses. International Journal of Clinical Pharmacology and Therapeutics, 2007. 45(10): p. 539-547.

1594. Zhu, Y.B., et al., Relative Bioavailability of Two Formulations of Nevirapine 200-mg Tablets in Healthy Chinese Male Volunteers: A Single-Dose, Randomized-Sequence, Open-Label, Two-Way Crossover Study. Clinical Therapeutics, 2010. 32(13): p. 2258-2264.

1595. Zhu, Y.L., et al., Effect Of Chinese Herb Danzhi Xiaoyao Pills On Pharmacokinetics Of Venlafaxine In Beagles. Drug Design Development and Therapy, 2019. 13: p. 3343-3355.

1596. Zimmerman, Y., W. Wouters, and H. Bennink, The bioequivalence of the contraceptive steroids ethinylestradiol and drospirenone is not affected by co-administration of dehydroepiandrosterone. European Journal of Contraception and Reproductive Health Care, 2013. 18(3): p. 206-214.

1597. Zimmermann, T., K.H. Wisser, and H. Dietrich, The effects of Valette (R) on skin and hair: A post-marketing surveillance study. International Journal of Clinical Practice, 2000. 54(2): p. 85-+.

1598. Zou, J.J., et al., Pharmacokinetic and bioequivalence comparison of a single 100-mg dose of cefteram pivoxil powder suspension and tablet formulations: a randomized-sequence, open-label, two-period crossover study in healthy chinese adult male volunteers. Clinical Therapeutics, 2008. 30(4): p. 654-660.

1599. Zuo, C.Z., et al., Effect of Fluconazole on the Pharmacokinetic Properties of Imrecoxib, a Novel NSAID: A Single-center, Open-label, Self-controlled Study in Healthy Chinese Male Volunteers. Clinical Therapeutics, 2018. 40(8): p. 1347-1356.

1600. Zuo, X.C., et al., Effect of CYP3A5*3 Polymorphism on Pharmacokinetic Drug Interaction between Tacrolimus and Amlodipine. Drug Metabolism and Pharmacokinetics, 2013. 28(5): p. 398-405.
